# Using artificial intelligence to read chest radiographs for tuberculosis detection: A multi-site evaluation of the diagnostic accuracy of three deep learning systems

**DOI:** 10.1038/s41598-019-51503-3

**Published:** 2019-10-18

**Authors:** Zhi Zhen Qin, Melissa S. Sander, Bishwa Rai, Collins N. Titahong, Santat Sudrungrot, Sylvain N. Laah, Lal Mani Adhikari, E. Jane Carter, Lekha Puri, Andrew J. Codlin, Jacob Creswell

**Affiliations:** 1 Stop TB Partnership, Chemin du Pommier 40, 1218 Le Grand-Saconnex, Geneva, Switzerland; 2 Tuberculosis Reference Laboratory Bamenda, PO Box 586, Bamenda, Cameroon; 3 International Organization for Migration, Migration Health Department, Kathmandu, Nepal; 4 Present Address: Bamenda Regional Hospital, PO Box 818 Bamenda, Cameroon; 5 Department of Medicine, Division of Pulmonary, Critical Care and Sleep, Warren Alpert Medical School, Brown University, Rhode Island, USA

**Keywords:** Tuberculosis, Radiography, Population screening

## Abstract

Deep learning (DL) neural networks have only recently been employed to interpret chest radiography (CXR) to screen and triage people for pulmonary tuberculosis (TB). No published studies have compared multiple DL systems and populations. We conducted a retrospective evaluation of three DL systems (CAD4TB, Lunit INSIGHT, and qXR) for detecting TB-associated abnormalities in chest radiographs from outpatients in Nepal and Cameroon. All 1196 individuals received a Xpert MTB/RIF assay and a CXR read by two groups of radiologists and the DL systems. Xpert was used as the reference standard. The area under the curve of the three systems was similar: Lunit (0.94, 95% CI: 0.93–0.96), qXR (0.94, 95% CI: 0.92–0.97) and CAD4TB (0.92, 95% CI: 0.90–0.95). When matching the sensitivity of the radiologists, the specificities of the DL systems were significantly higher except for one. Using DL systems to read CXRs could reduce the number of Xpert MTB/RIF tests needed by 66% while maintaining sensitivity at 95% or better. Using a universal cutoff score resulted different performance in each site, highlighting the need to select scores based on the population screened. These DL systems should be considered by TB programs where human resources are constrained, and automated technology is available.

## Introduction

It is almost impossible to talk about the future of medicine without stumbling upon two letters that bring many hopes, fears, and confusions to the topic. Artificial intelligence (AI) is not new but has gained traction in healthcare in the last decade, due in part to advances in deep learning neural networks. Neural networks are a set of algorithms organized in nodes and layers that mimic human cognitive functions, designed to automatically infer rules to recognize patterns^1,2^. Neural networks help us cluster and classify images, sound, text and time series after being trained on labeled datasets^1^. Deep-learning networks are distinguished from earlier versions of neural networks by having more than one hidden layer^1^, so that each layer learns a distinct set of characters and aggregates and combines inputs from the previous layers to understand and perform more complex features and functions, such as reading medical images and autonomous driving^1,3^.

Deep neural networks provide opportunities for new solutions to tackle tuberculosis (TB), which kills more people world-wide than any single infectious disease^4^. A major reason for this high mortality is the persistent gap in detection; more than one third of the estimated 10 million incident TB cases are not diagnosed and reported^4^. Chest x-ray (CXR) has historically been used in TB detection; for mass screenings^5^, and more recently for prevalence surveys and active case finding interventions^6,7^. It is recommended by the World Health Organization (WHO) as a triage test prior to the use of Xpert MTB/Rif^8^. However, CXR is of only limited use for TB diagnosis due to its modest specificity, since many diseases present with similar radiologic patterns^9,10^, high inter- and intra-reader variability and reproducibility^11,12^, and the paucity of skilled radiologists in many high TB burden countries^12^.

Several deep-learning (DL) systems have been developed in recent years to analyze digital chest radiographs for TB-related abnormalities that could potentially address current shortcomings, including reducing human inter-reader variability and reproducibility and supplying radiologic services where radiologists are not available. However, current evidence is limited to only one product, CAD4TB (Delft Imaging Systems, Netherlands)^6,13,14^ which has been evaluated only with non-DL versions of the software, as DL is new in the current version 6. No peer-reviewed evaluations of the performance of any DL system for detecting TB abnormalities exist, nor do any compare multiple DL systems with human readers. WHO has not made a recommendation on the use of automated reading systems for TB due to the current lack of evidence^8^. To fill the evidence gap, we compared the performance of three different DL applications in detecting bacteriologically-confirmed TB with that of radiologists experienced in detecting TB, using datasets from two countries.

## Methods

### Summary of DL systems

Through a literature review and the database of innovators developed under the Accelerator for Impact project at Stop TB Partnership, we identified and contacted eight DL system vendors regarding their interest in participating in the evaluation. Three DL systems with stable version control were included in this study: CAD4TB (version 6), qXR (version 2) developed by Qure.ai (India), and Lunit INSIGHT (Lunit) for Chest Radiography (Version 4.7.2) developed by Lunit (South Korea). We used the latest versions available of the three DL systems in this evaluation. CAD4TB version 6^15^ differs from previous versions by using DL. Both CAD4TB and Lunit read DICOM (Digital Imaging and Communications in Medicine) format only, while qXR can parse digital radiographs stored in PNG and JPEG. CAD4TB detects TB-specific abnormalities and outputs continuous abnormality scores ranging from 0 to 100. The greater the abnormality score, the higher probability of having TB. Current versions of qXR^16^ and Lunit^17^ detect several discrete pulmonary abnormalities, such as calcification, cavitation, opacities etc. Both systems present the final results for TB and the specific clinical abnormalities in binary (“Yes” / “No”) using a pre-defined threshold abnormality score. The abnormality scores for Lunit and qXR range from 0 to 100%. The default threshold abnormality score can be tuned based on screening requirements. All three DL systems can generate heat maps showing abnormalities.

### Study population and study setting

We conducted a retrospective evaluation of the three DL systems following the Standards for Reporting of Diagnostic Accuracy (STARD) Initiative on design and conduct of diagnostic accuracy evaluation^18^ using CXR images collected from Nepal and Cameroon as part of different studies^19,20^. Adults (aged 15 years or older) with symptoms suggestive of TB (cough more than 2 weeks, fever, night sweats, weight loss) were consecutively enrolled in the pulmonary outpatient department (OP) at B.P. Koirala Institute of Health Sciences (BPKIHS) in Eastern Nepal between 28 June to 24 December 2015 and in the general OP at the Tuberculosis Reference Laboratory Bamenda and the Bamenda Regional Hospital in Cameroon between 9 September 2015 and 15 April 2016. Each study participant received a posterior-anterior CXR using digital X-ray machines (Phillips DigitalDiagnost in Nepal and Carestream Direct View Classic CR in Cameroon).

In both sites, each CXR was classified as “abnormal” if any pulmonary abnormality was detected by human readers, regardless of the abnormality being TB-specific, active or old. In Nepal every radiograph was read twice by two groups of radiologists independently. The first read was done by a professor of radiology with a MD in radiology at BPKIHS with 21 years of experience; and the second read was done by a group of residents and junior radiologists on rotation at BPKIHS, all with MBBS and were students of MD in radiology with 3–5 years’ experience. In Cameroon, each radiograph was first read by a field radiologist with 9 years’ experience in radiology. Regardless of the results of the field radiologist, all CXR were then sent anonymously to a remote teleradiology company, called Teleradiology Solutions^21^, which was accredited in 2005 by Joint Commission.

All participants provided two sputum samples (one spot sputum sample collected during the outpatient visit and a next day morning sample). Smear and Xpert were performed for all individuals. If the initial Xpert test failed (no result, invalid, error) testing was repeated utilizing the same sample with this result recorded as final. Demographic, symptom and medical history data were collected.

While human reading was done prospectively, the three DL systems scored the images retrospectively. The images were transferred to the Lunit and qXR for their reading through Secured File Transfer Protocol (SFTP) from the Stop TB repository, and to Delft through cloud transfer. All machine reading was performed independently with the developers blinded to all testing, clinical and demographic data.

### Data analysis

We evaluated the overall performance of the three DL systems using continuous abnormality scores and the specific performances at certain threshold scores that meet different performance goals.

The abnormality scores of the three DL systems were disaggregated into Xpert-positive (RIF sensitive, RIF resistant, RIF indeterminate) and Xpert-negative groups. We also examined the systems’ performance among individuals with negative smear microscopy results as CXR is often used after a negative smear test. Receiver operating characteristic (ROC) curves were plotted, using Xpert as the reference, to show the trade-off between sensitivity and specificity. The areas under the curve (AUCs) were calculated as the primary index of accuracy of DL systems^22^. Head-to-head comparisons of the three DL systems were performed comparing the AUCs (the larger the AUC, the better overall performance of the comparator test to correctly identify diseases and non-diseased subjects). Equivalent AUCs do not imply that the sensitivity and specificity of different tests are identical at each point on the ROC curves; the curves may have the same overall area but different shapes. Because a high sensitivity is desired for a triage test, we further examined a restricted part of the ROCs of the three DL systems at a sensitivity level >90%.

Since there are no generally recommended threshold scores to use, we selected several indicators to evaluate different performance goals. First, to compare the performance of the three DL systems and experienced human readers to correctly identify images from people with and without bacteriologically confirmed TB, we calculated the threshold scores of DL systems corresponding to the observed sensitivity of each of the human readers and compared the corresponding specificity as well as accuracy, defined as the proportion of true positives and true negatives among the entire population. Second, since the coordinate (0,1) on the ROC plot, i.e. a perfect classification, represents 100% sensitivity and 100% specificity, we calculated the point on the ROCs closest to the coordinate (0,1)^23,24^ and reported the corresponding sensitivity, specificity and accuracy. Third, because FIND’s Target Product Profile (TPP) for a community-based triage or referral test for TB requires a sensitivity ≥95% and a specificity ≥80% when compared with the confirmatory test^25^, we identified the threshold scores to reach 95% sensitivity and calculated the corresponding specificity. Fourth, we calculated the sensitivity and specificity if the goal was to reduce by half (50%), two thirds (67%), and three quarters (75%) the number of Xpert tests needed for follow-on testing after a positive CXR triage test. Finally, we calculated the threshold score, sensitivity and specificity while achieving maximum accuracy.

All analyses were performed for each site separately as well as combined. Often, multiple threshold score results could satisfy a particular indicator. In these cases, the one which yielded the maximum sensitivity/specificity was selected. The sensitivity, specificity and accuracy of every point on the ROC curves is reported in the Supplementary Information.

All statistical analyses and graphs were produced using R version 3.5.1 (R Foundation for Statistical Computing, Vienna, Austria). Data were presented as median with interquartile ranges (IQR).

### Ethical approval and informed consent

The study protocols were reviewed and approved by the Institutional Reviewing Board at the B.P. Koirala Institute of Health Sciences and the National Ethics Committee of Cameroon. Verbal informed consent was obtained from each participant before they were enrolled in the study. The study was carried out in accordance with the relevant guidelines and regulations. Patient data, anonymized and coded with unique patient identifiers, were transferred and stored on a SFTP server.

### Role of the DL system developers

The CAR developers had no role in study design, data collection, analysis plan, or writing of the study. The developers only had access to the anonymized CXR images, and did not receive any of the demographic, symptom, medical, or testing data of the participants.

## Results

### Pooled study sites

A total of 1,196 individuals (515 from Nepal and 681 from Cameroon) were included in the study with a median age of 46 (IQR: 30–61). The prevalence of Xpert-positive TB was 9.1% (n = 109), 6.6% (n = 78) were smear positive, and 41.1% (n = 491) had an abnormal CXR according to the radiologists (Table 1).

**Table 1:** Demographic and Clinical Characteristics of People Screened with Chest X-ray and Xpert MTB/RIF.

	Overall			Nepal			Cameroon		
	Xpert Positive	Xpert Negative	Total	Xpert Positive	Xpert Negative	Total	Xpert Positive	Xpert Negative	Total
N	109	1087	1196	94	421	515	15	666	681
Age (median [IQR])	36 [25, 47]	48 [31, 61]	46 [30, 61]	38 [25, 48.75]	47 [29, 62]	45 [28, 61]	33 [25.50, 38]	48 [32, 61]	47 [32, 61]
Sex = F/M (%)	32/77 (29.4/70.6)	614/473 (56.5/43.5)	646/550 (54.0/46.0)	27/67 (28.7/71.3)	200/221 (47.5/52.5)	227/288 (44.1/55.9)	5/10 (33.3/66.7)	414/252 (62.2/37.8)	419/262 (61.5/38.5)
Cough (%)	105 (96.3)	632 (58.1)	737 (61.6)	93 (98.9)	368 (87.4)	461 (89.5)	12 (80)	264 (39.6)	276 (40.5)
Fever (%)	76 (69.7)	444 (40.8)	520 (43.5)	68 (72.3)	218 (51.8)	286 (55.5)	8 (53.3)	226 (33.9)	234 (34.4)
Weight loss (%)	74 (67.9)	550 (50.6)	624 (52.2)	62 (66)	150 (35.6)	212 (41.2)	12 (80)	400 (60.1)	412 (60.5)
Night Sweats (%)	37 (33.9)	269 (24.7)	306 (25.6)	29 (30.9)	83 (19.7)	112 (21.7)	8 (53.3)	186 (27.9)	194 (28.5)
**TB treatment history (%)**									
Yes	89 (81.7)	982 (90.3)	1071 (89.5)	20 (21.3)	76 (18.1)	96 (18.6)	0 (0)	22 (3.3)	22 (3.2)
No	0 (0.0)	7 (0.6)	7 (0.6)	74 (78.7)	345 (81.9)	419 (81.4)	15 (100)	637 (95.6)	652 (95.7)
Unknown	20 (18.3)	98 (9.0)	118 (9.9)				0 (0)	7 (1.1)	7 (1)
**HIV status (%)**									
Negative	17 (15.6)	254 (23.4)	271 (22.7)	9 (9.6)	50 (11.9)	59 (11.5)	8 (53.3)	204 (30.6)	212 (31.1)
Positive	10 (9.2)	28 (2.6)	38 (3.2)	4 (4.3)	3 (0.7)	7 (1.4)	6 (40)	25 (3.8)	31 (4.6)
Unknown	82 (75.2)	805 (74.1)	887 (74.2)	81 (86.2)	368 (87.4)	449 (87.2)	1 (6.7)	437 (65.6)	438 (64.3)
AFB positive (%)	76 (70.4)	2 (0.2)	78 (6.6)	67 (71.3)	1 (0.2)	68 (13.2)	9 (64.3)	1 (0.2)	10 (1.5)
Xpert positive (%)	109 (100.0)	0 (0.0)	109 (9.1)	94 (100)	0 (0)	94 (18.3)	15 (100)	0 (0)	15 (2.2)
CAD4TB (median [IQR])	86 [75, 99]	45 [25, 54]	46 [28, 59]	86 [77, 99]	48 [25, 69]	54 [40.5, 79.5]	77 [58, 99]	44 [25, 49]	44 [25, 50]
Lunit (median [IQR])	0.99 [0.97, 0.99]	0.08 [0.01, 0.51]	0.10 [0.01, 0.80]	0.99 [0.97, 0.99]	0.37 [0.05, 0.90]	0.69 [0.09, 0.96]	0.97 [0.90, 0.98]	0.03 [0.01, 0.15]	0.03 [0.01, 0.18]
qXR (median [IQR])	0.90 [0.82, 0.94]	0.17 [0.10, 0.36]	0.19 [0.11, 0.50]	0.92 [0.84, 0.94]	0.30 [0.12, 0.65]	0.44 [0.14, 0.82]	0.78 [0.64, 0.86]	0.14 [0.09, 0.23]	0.15 [0.10, 0.24]
Senior Radiologist (Nepal) = Normal CXR (%)				4 (4.3)	203 (48.2)	207 (40.2)			
Junior Radiologist & Residents (Nepal) = Normal CXR (%)				12 (12.8)	290 (68.9)	302 (58.6)			
Radiologist (Cameroon) = Normal CXR (%)							3 (20)	495 (74.3)	498 (73.1)
Teleradiology Company (Cameroon) = Normal CXR (%)							3 (20)	494 (74.2)	497 (73.0)

**Figure 1:**
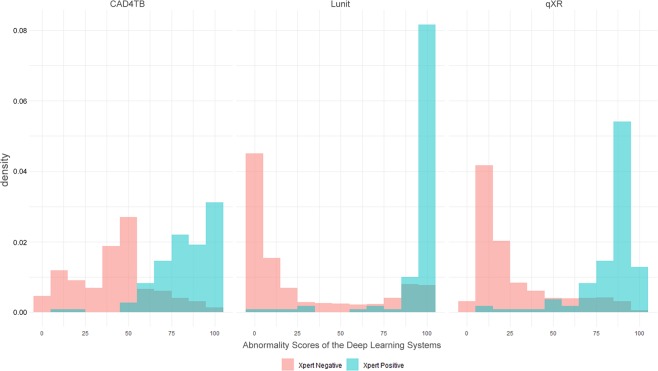
Frequency distribution of the abnormality scores of CAD4TB, Lunit, and qXR.

The different distributions of the abnormality scores of the three DL systems disaggregated by Xpert result are shown in Fig. 1. Although the distribution of the abnormality scores from Xpert positive patients overlaps with that from Xpert negative patients, the distributions of the abnormality scores were heavily left-skewed in the Xpert positive group, and right-skewed in the Xpert negative group. The degree of skewness was more profound in Lunit’s scoring than in CAD4TB’s and qXR’s scoring in both Xpert positive and Xpert negative groups. The skewness of scores of Lunit, qXR, and CAD4TB was −3.77, −2.45 and −1.64 in Xpert positive group, and 1.03, 1.32, and 0.16 in Xpert negative group, respectively, representing a good separation of individuals with and without TB.

**Figure 2:**
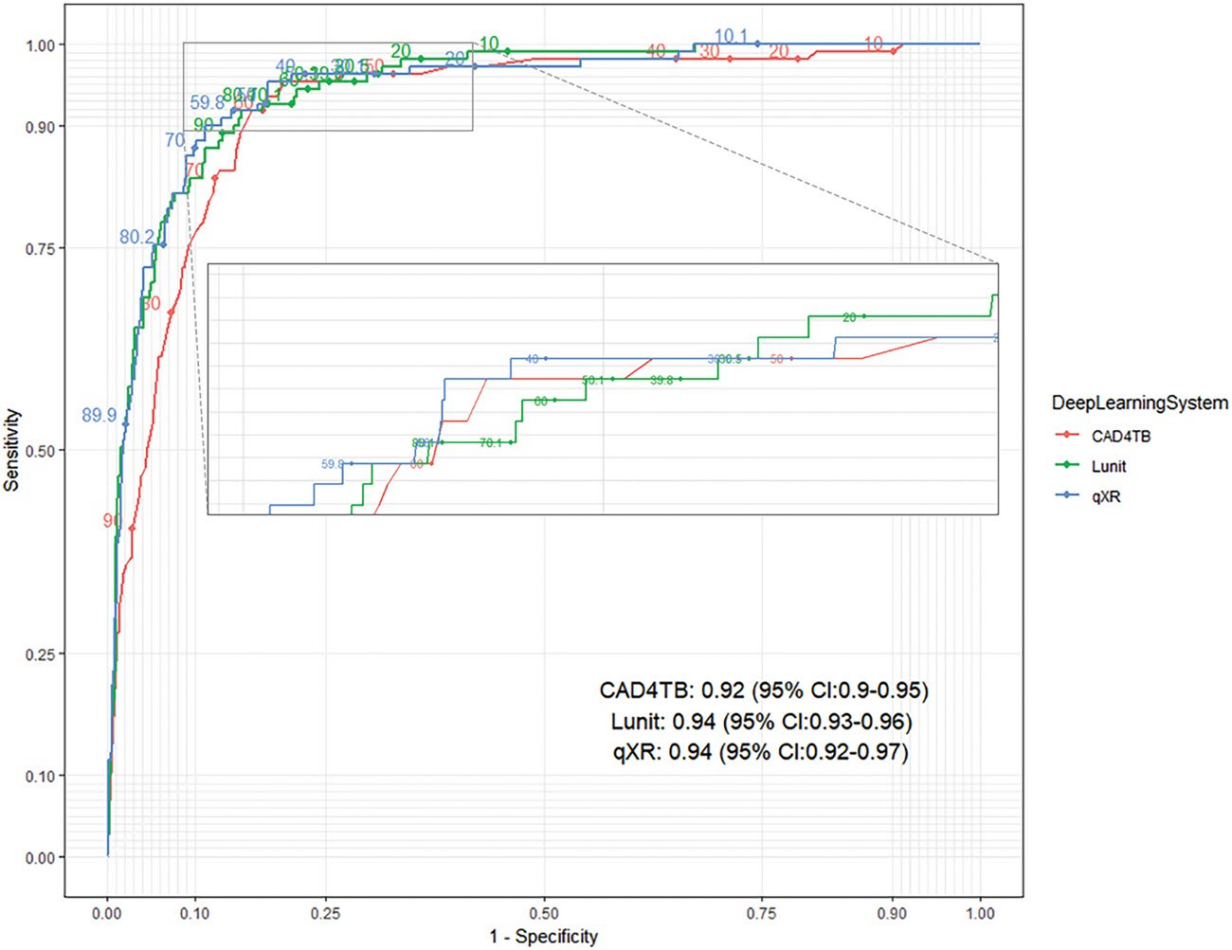
The ROC curves of CAD4TB (v6), Lunit (v4.7.2) and qXR (v2) using Xpert results as the reference (n = 1196).

**Figure 3:**
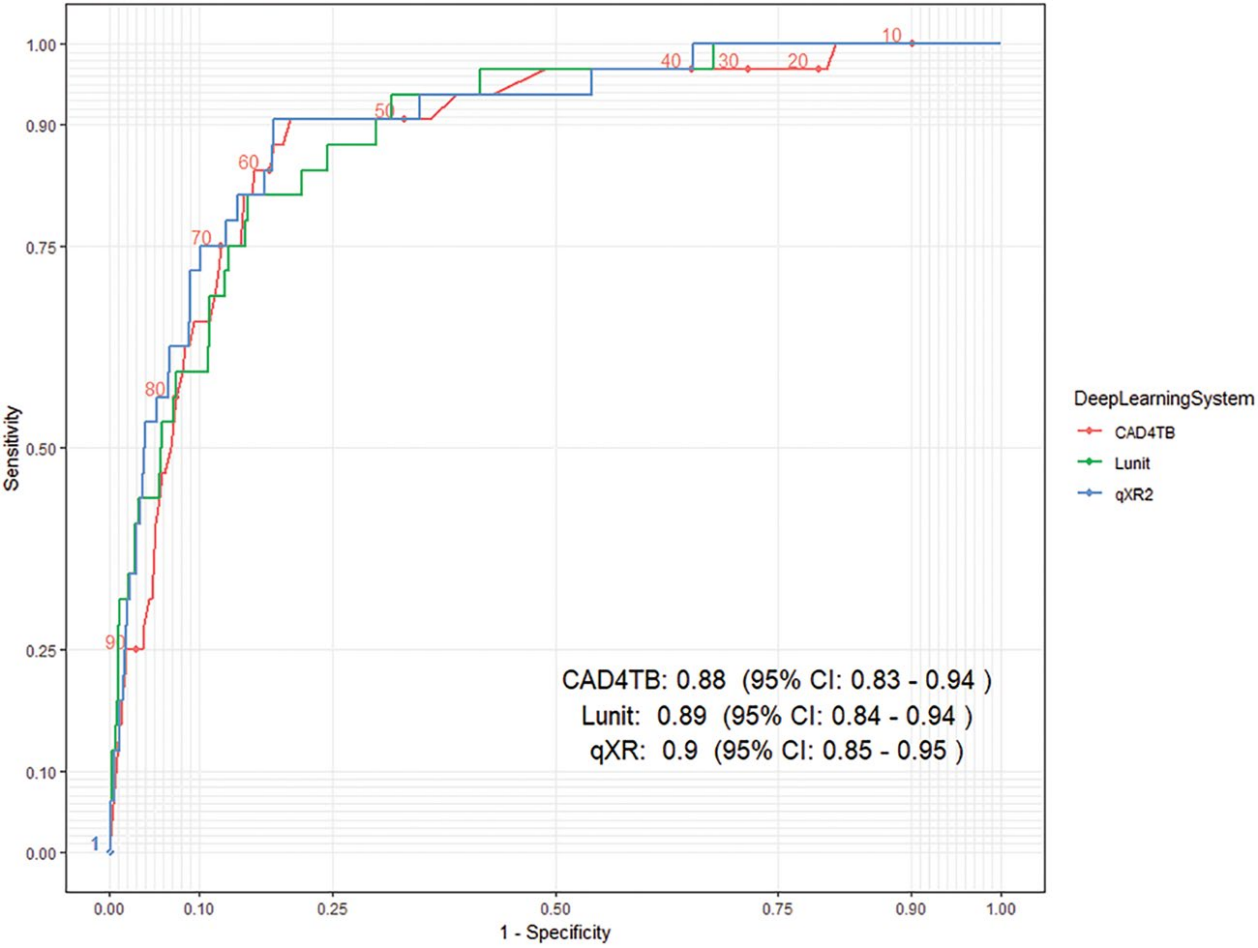
The ROC curves of CAD4TB (v6), Lunit (v4.7.2) and qXR (v2) among individuals with negative smear (n = 1102)

The ROC curves of the three DL systems were all well above the diagonal line (or “the line of no-discrimination”, representing random guessing) (Fig. 2). Both Lunit (0.94, 95% CI: 0.93–0.96) and qXR (0.94, 95% CI: 0.92–0.97) had higher AUC point estimates than CAD4TB (0.92, 95% CI: 0.90–0.95), but the differences were not statistically significant. qXR performed better when the sensitivity was between 90% and 96%; however, the confidence intervals of all three DL systems above 90% overlap (Fig. 2 and Supplementary Information). When we restricted the analysis to the 1102 people with negative smear results, the performance of the DL systems was similar to when all 1,196 individuals were considered (Fig. 3).

We calculated the accuracy, sensitivity and specificity of all four groups of radiologists (two from each site) and compared them with the three DL systems. When matching the sensitivity level of the human readers, the specificities of Lunit, qXR were significantly higher than the specificity of the four groups of human readers, yet they did not have significant differences among themselves. (Table 2). While the specificity of CAD4TB was significantly higher than that of the senior Nepali radiologist, the Cameroonian field radiologist and the teleradiology company, the difference was not significant when compared with the specificity of the group of residents and junior radiologists (Table 2). The accuracy of the CAD4TB and Lunit were greater than the four groups of human readers when matched by sensitivity, while the accuracy of qXR was higher than all human readers except the group of residents and junior radiologists (Table 2).

**Table 2:** Radiologist and Deep Learning System Performance for Chest Radiographs and Tuberculosis.

	Human Readers			Deep Learning Systems								
				CAD4TB (v6)			Lunit (v4.7.2)			qXR (v2)		
	Accuracy	Sensitivity (95%CI)	Specificity (95% CI)	Accuracy	Sensitivity (95% CI)	Specificity (95% CI)	Accuracy	Sensitivity (95% CI)	Specificity (95%CI)	Accuracy	Sensitivity (95% CI)	Specificity (95% CI)
**Nepal**												
Senior Radiologist	0.57	0.96	0.48	0.74	0.96	0.69	0.67	0.96	0.6	0.7	0.97*	0.65
		(0.89–0.99)	(0.43–0.53)		(0.89–0.99)	(0.64–0.73)		(0.89–0.99)	(0.55–0.65)		(0.91–0.99)	(0.6–0.69))
Junior Radiologist & Residents	0.72	0.87	0.69	0.77	0.87	0.75	0.85	0.87	0.78	0.69	0.87	0.81
		(0.79–0.93)	(0.64–0.73)		(0.79–0.93)	(0.71–0.79)		(0.79–0.93)	(0.73–0.82)		(0.79–0.93)	(0.76–0.84)
**Cameroon**												
Radiologist	0.74	0.8	0.74	0.9	0.8	0.9	0.94	0.8	0.94	0.94	0.8	0.95
		(0.52–0.96)	(0.71–0.78)		(0.52–0.96)	(0.87–0.92)		(0.52–0.96)	(0.92–0.96)		(0.52–0.96)	(0.93–0.96)
Teleradiology Company	0.74	0.8	0.74	0.9	0.8	0.9	0.94	0.8	0.94	0.94	0.8	0.95
		(0.52–0.96)	(0.71–0.77)		(0.52–0.96)	(0.87–0.92)		(0.52–0.96)	(0.92–0.96)		(0.52–0.96)	(0.93–0.96)

*The sensitivity of qXR version 2 closest to that of the senior radiologist is 97%, instead of 96%.

At the threshold scores that were closest to the coordinate (0,1), the sensitivities of the three DL systems fell between 87%-91% and the specificities between 84–89% (Table 3). The maximum specificity while keeping the sensitivity above 95% was 80% (77–82%) for CAD4TB, 76% (73–78%) for Lunit, and 72% (69–75%) for qXR (Table 3). At 95% sensitivity, eight TB patients were missed by at least one of the DL systems, of whom five were considered by 2 the initial readers as normal and two as non-TB abnormal by at least one initial reader. An experienced pulmonologist (EJC) with 31-year experience reviewed and annotated these images (Table 4). There were two patients that were missed by all DL systems, of which one was graded by the 2 radiologists and senior pulmonologists as “normal”, while the other was considered abnormal only by the teleradiology company and the senior pulmonologist (EJC).

**Table 3:** Performance of CAD4TB (v6), Lunit (v4.7.2), and qXR (v2) at Selected Thresholds.

Thresholds	CAD4TB (v6)				Lunit (v4.7.2)				qXR (v2)			
	Threshold	Accuracy	Sensitivity (95% CI)	Specificity (95% CI)	Threshold	Accuracy	Sensitivity (95% CI)	Specificity (95% CI)	Threshold	Accuracy	Sensitivity (95% CI)	Specificity (95% CI)
ROC01#	63	0.85	0.91 (0.84–0.96)	0.84 (0.82–0.86)	0.92	0.89	0.87 (0.79–0.93)	0.89 (0.87–0.91)	0.67	0.86	0.88 (0.8–0.93)	0.89 (0.87–0.91)
Sensitivity ≥95%	57	0.81	0.95 (0.9–0.98)	0.8 (0.77–0.82)	0.55	0.77	0.95 (0.9–0.98)	0.76 (0.73–0.78)	0.49	0.83	0.95 (0.90–0.98)	0.82 (0.79–0.84)
Reduce Xpert tests by 1/2	47	0.61	0.97 (0.92–0.99)	0.57 (0.54–0.6)	0.11	0.6	0.99 (0.95–1)	0.56 (0.53–0.59)	0.18	0.57	0.97 (0.92–0.99)	0.53 (0.5–0.56)
Reduce Xpert tests by 2/3	53	0.75	0.96 (0.91–0.99)	0.73 (0.7–0.76)	0.39	0.74	0.95 (0.9–0.98)	0.72 (0.69–0.74)	0.34	0.75	0.96 (0.91–0.99)	0.73 (0.7–0.76)
Reduce Xpert tests by 3/4	59	0.82	0.94 (0.87–0.97)	0.82 (0.79–0.84)	0.79	0.82	0.93 (0.86–0.97)	0.81 (0.79–0.84)	0.5	0.83	0.93 (0.86–0.97)	0.82 (0.8–0.84)
Max Accuracy	88	0.92	0.47 (0.37–0.57)	0.96 (0.95–0.97)	0.98	0.94	0.58 (0.48–0.67)	0.97 (0.96–0.98)	0.84	0.94	0.71 (0.61–0.79)	0.96 (0.94–0.97)

^#^ROC01: the point on the ROC that was closest to the coordinates (0,1), the perfect classification.

**Table 4:** Individuals with Bacteriologically Confirmed TB, Missed by Deep Learning Systems at 95% Sensitivity.

Individual Missed	Senior Radiologist (Nepal)	Junior Radiologist & Residents (Nepal)	Field Radiologist (Cameroon)	Teleradiology Company (Cameroon)	*DL reading*	CAD4TB score	qXR score	Lunit score	Annotation by a senior pulmonologist
Nepal 1	Normal	Normal	*NA*	*NA*	*Missed by all 3 DL systems*	9	0.1205	0.2330	Normal
Nepal 2	Abnormal	Abnormal	*NA*	*NA*	*Missed by qXR*	65	0.4225	0.9853	Abnormal: may be an azygous lobe (normal variant) but also could be apical TB
Nepal 3	Abnormal	Abnormal	*NA*	*NA*	*Missed by Lunit*	71	0.5223	0.3458	Abnormal maybe old scar: minimal tenting of the right diaphragm
Nepal 4	Normal	Normal	*NA*	*NA*	*Missed by Lunit and qXR*	63	0.1517	0.1318	Non-TB abnormality: elevated right hemidiaphragm
Nepal 5	Normal	Normal	*NA*	*NA*	*Missed by CAD4TB and Lunit*	46	0.4992	0.2728	Normal
Cameroon 1	*NA*	*NA*	Normal	Abnormal	*Missed by all 3 DL systems*	48	0.1259	0.0176	Abnormal in the left mid lung field by the heart- could be TB but not classic
Cameroon 2	*NA*	*NA*	Normal	Normal	*Missed by CAD4TB*	53	0.6695	0.8744	Abnormal: ill defined infiltrate in the right upper lung field
Cameroon 3	*NA*	*NA*	Normal	Normal	*Missed by CAD4TB and qXR*	18	0.2504	0.5518	Normal lung field but cardiomegaly

When the goal was to reduce the number of follow-on Xpert tests by half, the sensitivities for all three DL systems remained high, between 97–99% with no statistical difference among the DL systems. Similarly reducing the follow-on Xpert tests by two thirds and three quarters, the sensitivities of DL systems reduced to between 95–96% and 93–94% respectively without significant differences between the three DL systems (Table 3). The highest accuracy of the three DL systems were between 0.92 and 0.94; however, the corresponding sensitivity was between 47–71% limiting its usefulness as an indicator. The sensitivity, specificity and accuracy of every point on the three ROC curves are reported in the Supplementary Information.

### Individual study sites

**Figure 4:**
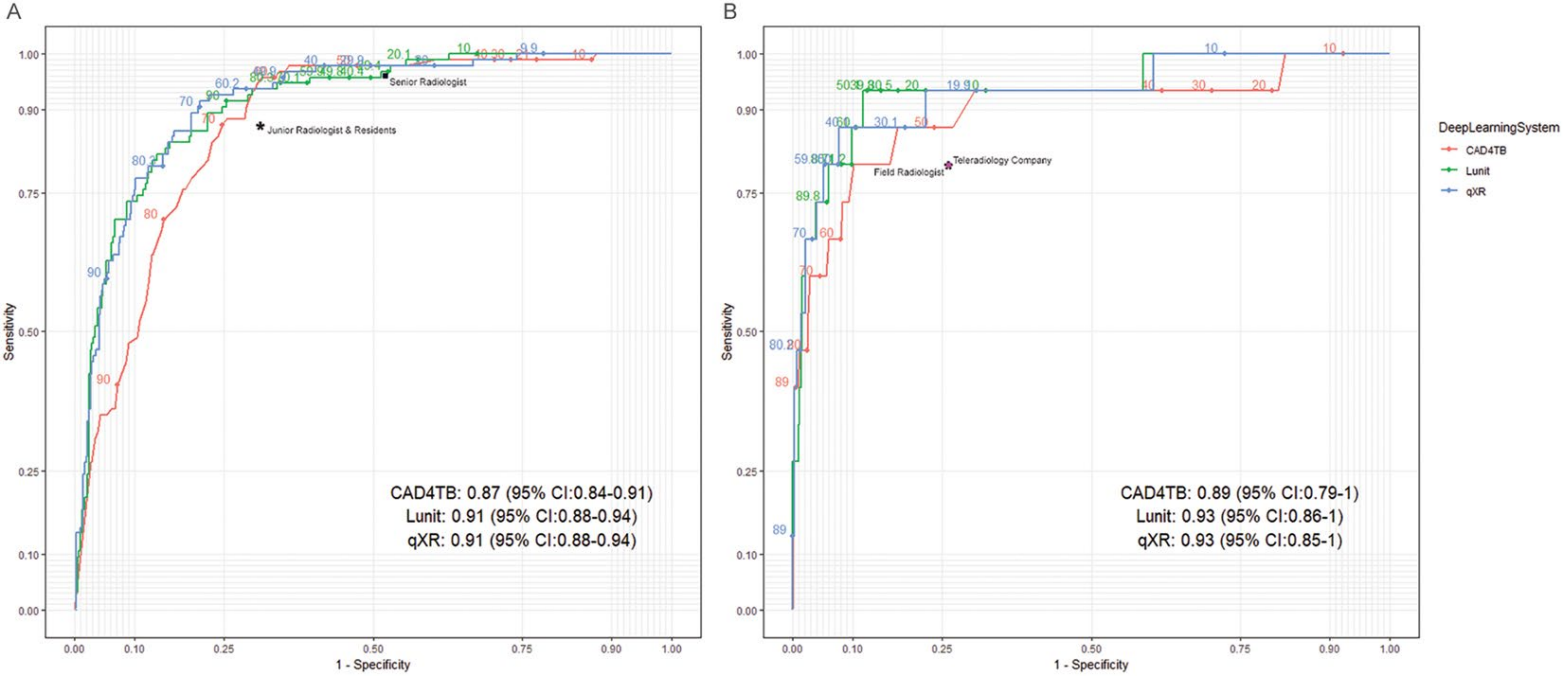
(**a**) The ROC curves of CAD4TB (v6), Lunit (v4.7.2) and qXR (v2) in Nepal (n = 515). (**b**) The ROC curves of CAD4TB (v6), Lunit (v4.7.2) and qXR (v2) in Cameroon (n = 681).

The three DL systems also performed similarly when stratified by study site despite demographic difference between the sites. In Nepal, 94 (18.3%) were Xpert positive, and 68 (13.2%) were smear positive. The AUCs of CAD4TB, Lunit, and qXR were 0.87 (95% CI: 0.84–0.91), 0.91 (95% CI: 0.88–0.94), and 0.91 (95% CI: 0.88–0.94), respectively (Fig. 4a).

In Cameroon, 15 (2.2%) were Xpert positive, and 10 (1.5%) were smear positive. The point estimates of the AUCs were higher than in Nepal. The Cameroon AUCs of CAD4TB, Lunit, and qXR were 0.90 (95% CI: 0.79–1), 0.93 (95% CI: 0.86–1), and 0.93 (95% CI: 0.85–1), respectively (Fig. 4b).

The different cutoff thresholds were higher in Nepal than Cameroon. For example, the cutoff threshold to maximize specificity while keeping sensitivity above 95% for CAD4TB was 63 in Nepal but 48 in Cameroon (Table 5). If the thresholds were kept nominally the same, the corresponding sensitivities and specificities changed. For instance, CAD4TB at the threshold score of 63 had a sensitivity of 95% (95%CI: 88–98%) and specificity of 69% (95%CI: 65–74%); in Nepal while using the same 63 threshold produced a sensitivity of 67% (95%CI: 38–88%) and a specificity of 93% (95%CI: 91–95%) (data shown in Supplementary Information).

**Table 5:** Performance of CAD4TB (v6), Lunit (v4.7.2), and qXR (v2) at Selected Thresholds Stratified by Study Sites.

	CAD4TB (v6)				Lunit (v4.7.2)				qXR (v2)			
	Thresholds	Accuracy	Sensitivity (95%CI)	Specificity (95%CI)	Thresholds	Accuracy	Sensitivity (95%CI)	Specificity (95%CI)	Thresholds	Accuracy	Sensitivity (95%CI)	Specificity (95%CI)
**Nepal**												
Sensitivity ≥95%	63	0.74	0.95 (0.88–0.98)	0.69 (0.65–0.74)	0.8	0.71	0.95 (0.88–0.98)	0.66 (0.61–0.7)	0.52	0.72	0.95 (0.88–0.98)	0.67 (0.62–0.71)
Reduce Xpert tests by 1/2	55	0.68	0.98 (0.93–1)	0.61 (0.56–0.66)	0.69	0.67	0.96 (0.89–0.99)	0.6 (0.55–0.65)	0.44	0.67	0.97 (0.91–0.99)	0.61 (0.56–0.65)
Reduce Xpert tests by 2/3	73	0.78	0.81 (0.71–0.88)	0.78 (0.73–0.82)	0.93	0.79	0.87 (0.79–0.93)	0.78 (0.73–0.82)	0.7	0.81	0.9 (0.83–0.96)	0.79 (0.75–0.83)
Reduce Xpert tests by 3/4	80	0.82	0.7 (0.6–0.79)	0.85 (0.81–0.88)	0.96	0.85	0.81 (0.71–0.88)	0.86 (0.83–0.9)	0.82	0.86	0.79 (0.69–0.86)	0.88 (0.84–0.91)
Max Accuracy	94	0.85	0.35 (0.26–0.46)	0.96 (0.93–0.97)	0.98	0.89	0.63 (0.52–0.73)	0.94 (0.92–0.96)	0.88	0.88	0.67 (0.57–0.76)	0.92 (0.89–0.95)
**Cameroon**												
Sensitivity ≥95%	48	0.7	0.93* (0.68–1)	0.7 (0.66–0.73)	0.55	0.88	0.93* (0.68–1)	0.88 (0.86–0.91)	0.25	0.78	0.93* (0.68–1)	0.78 (0.74–0.81)
Reduce Xpert tests by 1/2	45	0.54	0.93 (0.68–1)	0.53 (0.5–0.57)	0.03	0.51	0.93 (0.68–1)	0.5 (0.46–0.53)	0.14	0.48	0.93 (0.68–1)	0.47 (0.44–0.51)
Reduce Xpert tests by 2/3	47	0.66	0.93 (0.68–1)	0.65 (0.61–0.69)	0.1	0.68	0.93 (0.68–1)	0.68 (0.64–0.71)	0.2	0.7	0.93 (0.68–1)	0.69 (0.66–0.73)
Reduce Xpert tests by 3/4	50	0.77	0.87 (0.6–0.98)	0.76 (0.73–0.79)	0.17	0.77	0.93 (0.68–1)	0.76 (0.73–0.79)	0.24	0.77	0.93 (0.68–1)	0.77 (0.74–0.8)
Max Accuracy	90	0.99	0.4 (0.16–0.68)	1 (0.99–1)	0.97	0.98	0.4 (0.16–0.68)	0.99 (0.98–0.99)	0.77	0.98	0.53 (0.27–0.79)	0.99 (0.97–0.99)

^*^Due to the limited number of Xpert-positive patients in the site in Cameroon, the sensitivity closest to 95% is 93%.

## Discussion

This is the first evaluation of multiple DL systems for detecting TB abnormalities in CXR. We observed that all three systems performed significantly better than human radiologists and had higher AUCs than most of the current published literature on previous versions of CAD4TB^6^. Our results also document the first published evaluation of qXR and Lunit for detecting TB. There was no statistical difference among the AUCs of CAD4TB, Lunit, and qXR across the study sites, in pooled analysis, and when only smear negative individuals were considered. The point estimate for qXR and CAD4TB met the TPP target for a community-based triage test. However, there was no statistical difference between the specificity of CAD4TB and Lunit at the sensitivity level of 95% and a marginal difference with qXR. The overall performance of the three DL systems was similar in multiple analyses and stratifications. Implementers considering using DL systems for CXR reading should take into account other factors including service, ease of use, maintenance and price - all important considerations in any new technology implementation^26^.

This study demonstrates that these DL systems have the potential to increase capacity and aid TB diagnosis, especially in settings with a shortage of trained human readers which have been noted as shortcomings in CXR use^8^. When we compared the performance of DL systems and radiologists, all three systems were better than human readers in detecting bacteriologically confirmed TB. Although past publications on previous versions of CAD4TB had contradictory findings^13,27–30^, our study evaluated the latest version of CAD4TB, showing improved performance. There are a number of other DL systems that are at different stages of development and commercialization^31^. As new CAR products and new versions enter the market at a pace quicker than other types of TB diagnostics, it will be critical to monitor the performance of these successive digital products and versions.

Heads of State in the United Nations High Level Meeting (UNHLM) on TB in 2018 committed to urgent global responses to end TB, including diagnosing and treating a cumulative 40 million people by 2022^32^. Significant financial investments in diagnosis are needed to achieve UNHLM commitments. This study demonstrates that DL applications can be used to triage patients in order to reduce the number of expensive follow-on tests, while still maintaining high sensitivity. When the threshold score was set to reduce by half the number of Xpert tests, the sensitivities of the three DL applications were still between 97%-99% (Table 3). Even a two thirds or three quarters reduction in follow-on Xpert testing using the DL systems only reduced the sensitivity to 95–96% and 93–94%, respectively (Table 3). We observed that as Xpert tests were reduced, the sensitivity only slightly decreased while the specificity and accuracy increased greatly, which matches the observation from the ROC curves of the three DL systems. The ROC curves were relative flat with small slopes at a high sensitivity level (above 90%), which means a large gain in specificity (moving leftward on the x-axis) only come at a small decrease in sensitivity (moving downward on the y- axis). This is a hallmark of a good diagnostic test.

This study included two datasets with different prevalence of TB. The high prevalence of TB among the study population in Nepal is similar to targeted facility-based case finding or routine passive case finding, where the yield has been 10–20% in many countries^33^. The lower TB prevalence among the study population in Cameroon is similar to the expectations of active case finding^34^. The results stratified by sites were similar to the findings from the general analysis with no statistical difference among the DL systems.

The observation that DL system performance at similar thresholds could vary greatly is of critical importance to implementers. For example, in the high TB prevalence case finding study in Nepal, CAD4TB had a sensitivity of 95% at the threshold score of 63, but the threshold score would need to be lowered to 48 in the lower TB prevalence case finding study in Cameroon to reach the same sensitivity. While the current WHO guidance for these computer-aided detection software for TB emphasizes the need of predefined threshold scores^8^, our results clearly indicate the need for implementers to conduct their own pilot on the specific population being tested. A previous study found differences in performance by age and by referral site^13^. With large datasets, it may be possible to tailor specific thresholds depending on the characteristics of individuals screened. In some published literature, specific threshold scores for different versions of CAD4TB have been used and following these scores or manufactures default settings may produce different results across settings.

There are a number of limitations in our study. Due to logistic and budgetary constraints, we did not use culture as the reference standard. Using Xpert as the reference standard has the potential to bias the diagnostic accuracy assessment due to limited sensitivity for smear-negative TB compared to culture: however, WHO recommends Xpert use as a reference in evaluations of the computer-aided detection software^8^. We were not able to obtain the HIV status of some of the participants in both sites, limiting our ability to analyze the products in this population. Lastly, since we retrospectively collected and analyzed the radiographs in the sites where CAD4TB was implemented, the radiographs had been read by earlier versions of CAD4TB. However, neither qXR nor Lunit had seen the images prior to this study.

## Conclusion

Lunit and qXR performed as well as CAD4TB across different analysis metrics and all three DL automated reading systems outperformed experienced human readers in differentiating people with bacteriologically confirmed TB and those without. While only qXR and CAD4TB technically met FIND’s TPP for a triage test of ≥95% sensitivity and ≥80% specificity in this analysis, all three products had similar performance and can be used to reduce the number of follow-on tests while keeping sensitivity high, providing a cost savings that could be applied toward proposed equipment and introduction costs of an DL system. The principle of AI is that performance will improve with exposure to additional training examples. These new technologies therefore have the potential to augment the capacity of, and improve overall TB diagnosis and care, especially in settings with a shortage of trained radiologists.

## Supplementary information

Supplementary File

## Data Availability

Data and materials used in this study are available upon reasonable request to the corresponding author and under a collaboration agreement.
