# Supplementary material for: Using artificial intelligence to read chest radiographs for tuberculosis detection: A multi-site evaluation of the diagnostic accuracy of three deep learning systems
Source: Sci Rep. 2019 Oct 18;9:15000. doi: 10.1038/s41598-019-51503-3 (PMC6802077; doi:10.1038/s41598-019-51503-3)
Supplement: Supplementary file 1 — Supplementary File [file 41598_2019_51503_MOESM1_ESM.pdf]

Supplementary Information

Using artificial intelligence to read chest radiographs for tuberculosis detection:

A multi-site evaluation of the diagnostic accuracy of three deep learning systems

Authors:

Zhi Zhen Qin, MPH[1], Melissa S. Sander PhD[2], Bishwa Rai MPH[3], Collins N. Titahong, MS[2], Santat Sudrungrot, MS[3], Sylvain N. Laah, MD[2], Lal Mani Adhikari, PhD[3], E. Jane Carter, MD[4], Lekha Puri, MScPH[1], Andrew J. Codlin MPH[1], Jacob Creswell, PhD*[1]

Affiliations:

1 Stop TB Partnership, Chemin du Pommier 40, 1218 Le Grand-Saconnex, Geneva, Switzerland

2 Tuberculosis Reference Laboratory Bamenda, PO Box 586, Bamenda, Cameroon

3 International Organization for Migration, Migration Health Department, Kathmandu, Nepal

4 Department of Medicine, Division of Pulmonary, Critical Care and Sleep, Warren Alpert Medical School, Brown University, Rhode Island, USA

*Corresponding Author:

Jacob Creswell PhD

Stop TB Partnership, Chemin du Pommier 40, 1218 Le Grand-Saconnex

Geneva, Switzerland

Email jacobc@stoptb.org

Phone +41 79 729 1520

Site	Deep Learning System	Abnormality Score	Sensitivity	Specificity	Positive Predictive Value	Negative Predictive Value	Xpert Saving	Accuracy
Pooled	CAD4TB	1	1 (0.97-1)	0 (0-0)	0.09 (0.08-0.11)	NA	0	0.09
Pooled	CAD4TB	2	1 (0.97-1)	0 (0-0.01)	0.09 (0.08-0.11)	1 (0.4-1)	0	0.09
Pooled	CAD4TB	3	1 (0.97-1)	0.01 (0-0.02)	0.09 (0.08-0.11)	1 (0.69-1)	0.01	0.1
Pooled	CAD4TB	4	1 (0.97-1)	0.02 (0.01-0.03)	0.09 (0.08-0.11)	1 (0.85-1)	0.02	0.11
Pooled	CAD4TB	5	1 (0.97-1)	0.03 (0.02-0.05)	0.09 (0.08-0.11)	1 (0.91-1)	0.03	0.12
Pooled	CAD4TB	6	1 (0.97-1)	0.05 (0.03-0.06)	0.1 (0.08-0.11)	1 (0.93-1)	0.04	0.13
Pooled	CAD4TB	7	1 (0.97-1)	0.06 (0.05-0.07)	0.1 (0.08-0.11)	1 (0.94-1)	0.05	0.14
Pooled	CAD4TB	8	1 (0.97-1)	0.08 (0.06-0.1)	0.1 (0.08-0.12)	1 (0.96-1)	0.07	0.16
Pooled	CAD4TB	9	1 (0.97-1)	0.09 (0.07-0.11)	0.1 (0.08-0.12)	1 (0.96-1)	0.08	0.17
Pooled	CAD4TB	10	0.99 (0.95-1)	0.1 (0.08-0.12)	0.1 (0.08-0.12)	0.99 (0.95-1)	0.09	0.18
Pooled	CAD4TB	11	0.99 (0.95-1)	0.11 (0.09-0.13)	0.1 (0.08-0.12)	0.99 (0.96-1)	0.1	0.19
Pooled	CAD4TB	12	0.99 (0.95-1)	0.12 (0.11-0.15)	0.1 (0.08-0.12)	0.99 (0.96-1)	0.11	0.2
Pooled	CAD4TB	13	0.99 (0.95-1)	0.13 (0.11-0.15)	0.1 (0.09-0.12)	0.99 (0.96-1)	0.12	0.21
Pooled	CAD4TB	14	0.99 (0.95-1)	0.14 (0.12-0.16)	0.1 (0.09-0.12)	0.99 (0.96-1)	0.13	0.22
Pooled	CAD4TB	15	0.99 (0.95-1)	0.15 (0.13-0.18)	0.11 (0.09-0.13)	0.99 (0.97-1)	0.14	0.23
Pooled	CAD4TB	16	0.99 (0.95-1)	0.17 (0.14-0.19)	0.11 (0.09-0.13)	0.99 (0.97-1)	0.15	0.24
Pooled	CAD4TB	17	0.99 (0.95-1)	0.17 (0.15-0.2)	0.11 (0.09-0.13)	0.99 (0.97-1)	0.16	0.25
Pooled	CAD4TB	18	0.99 (0.95-1)	0.19 (0.16-0.21)	0.11 (0.09-0.13)	1 (0.97-1)	0.17	0.26
Pooled	CAD4TB	19	0.98 (0.94-1)	0.2 (0.17-0.22)	0.11 (0.09-0.13)	0.99 (0.97-1)	0.18	0.27
Pooled	CAD4TB	20	0.98 (0.94-1)	0.21 (0.18-0.23)	0.11 (0.09-0.13)	0.99 (0.97-1)	0.19	0.28
Pooled	CAD4TB	21	0.98 (0.94-1)	0.22 (0.19-0.24)	0.11 (0.09-0.13)	0.99 (0.97-1)	0.2	0.29
Pooled	CAD4TB	22	0.98 (0.94-1)	0.22 (0.2-0.25)	0.11 (0.09-0.13)	0.99 (0.97-1)	0.2	0.29
Pooled	CAD4TB	23	0.98 (0.94-1)	0.23 (0.21-0.26)	0.11 (0.09-0.14)	0.99 (0.97-1)	0.21	0.3
Pooled	CAD4TB	24	0.98 (0.94-1)	0.24 (0.22-0.27)	0.12 (0.1-0.14)	0.99 (0.97-1)	0.22	0.31
Pooled	CAD4TB	25	0.98 (0.94-1)	0.25 (0.22-0.28)	0.12 (0.1-0.14)	0.99 (0.97-1)	0.23	0.32
Pooled	CAD4TB	26	0.98 (0.94-1)	0.26 (0.23-0.28)	0.12 (0.1-0.14)	0.99 (0.97-1)	0.23	0.32
Pooled	CAD4TB	27	0.98 (0.94-1)	0.26 (0.24-0.29)	0.12 (0.1-0.14)	0.99 (0.98-1)	0.24	0.33
Pooled	CAD4TB	28	0.98 (0.94-1)	0.27 (0.24-0.3)	0.12 (0.1-0.14)	0.99 (0.98-1)	0.25	0.33
Pooled	CAD4TB	29	0.98 (0.94-1)	0.28 (0.25-0.31)	0.12 (0.1-0.14)	0.99 (0.98-1)	0.26	0.34
Pooled	CAD4TB	30	0.98 (0.94-1)	0.29 (0.26-0.31)	0.12 (0.1-0.14)	0.99 (0.98-1)	0.26	0.35
Pooled	CAD4TB	31	0.98 (0.94-1)	0.29 (0.26-0.32)	0.12 (0.1-0.15)	0.99 (0.98-1)	0.27	0.35
Pooled	CAD4TB	32	0.98 (0.94-1)	0.3 (0.27-0.33)	0.12 (0.1-0.15)	0.99 (0.98-1)	0.28	0.36

Site	Deep Learning System	Abnormality Score	Sensitivity	Specificity	Positive Predictive Value	Negative Predictive Value	Xpert Saving	Accuracy
Pooled	CAD4TB	33	0.98 (0.94-1)	0.31 (0.28-0.33)	0.12 (0.1-0.15)	0.99 (0.98-1)	0.28	0.37
Pooled	CAD4TB	34	0.98 (0.94-1)	0.31 (0.28-0.34)	0.12 (0.1-0.15)	0.99 (0.98-1)	0.28	0.37
Pooled	CAD4TB	35	0.98 (0.94-1)	0.32 (0.29-0.35)	0.13 (0.11-0.15)	0.99 (0.98-1)	0.29	0.38
Pooled	CAD4TB	36	0.98 (0.94-1)	0.33 (0.3-0.35)	0.13 (0.11-0.15)	0.99 (0.98-1)	0.3	0.39
Pooled	CAD4TB	37	0.98 (0.94-1)	0.33 (0.3-0.36)	0.13 (0.11-0.15)	0.99 (0.98-1)	0.3	0.39
Pooled	CAD4TB	38	0.98 (0.94-1)	0.33 (0.31-0.36)	0.13 (0.11-0.15)	0.99 (0.98-1)	0.31	0.39
Pooled	CAD4TB	39	0.98 (0.94-1)	0.34 (0.31-0.37)	0.13 (0.11-0.15)	0.99 (0.98-1)	0.31	0.4
Pooled	CAD4TB	40	0.98 (0.94-1)	0.35 (0.32-0.38)	0.13 (0.11-0.16)	0.99 (0.98-1)	0.32	0.41
Pooled	CAD4TB	41	0.98 (0.94-1)	0.36 (0.33-0.39)	0.13 (0.11-0.16)	0.99 (0.98-1)	0.33	0.41
Pooled	CAD4TB	42	0.98 (0.94-1)	0.36 (0.33-0.39)	0.13 (0.11-0.16)	0.99 (0.98-1)	0.33	0.42
Pooled	CAD4TB	43	0.98 (0.94-1)	0.37 (0.34-0.4)	0.13 (0.11-0.16)	1 (0.98-1)	0.34	0.42
Pooled	CAD4TB	44	0.98 (0.94-1)	0.41 (0.38-0.44)	0.14 (0.12-0.17)	1 (0.98-1)	0.37	0.46
Pooled	CAD4TB	45	0.98 (0.94-1)	0.47 (0.44-0.5)	0.16 (0.13-0.19)	1 (0.99-1)	0.43	0.51
Pooled	CAD4TB	46	0.98 (0.94-1)	0.51 (0.48-0.54)	0.17 (0.14-0.2)	1 (0.99-1)	0.47	0.56
Pooled	CAD4TB	47	0.97 (0.92-0.99)	0.57 (0.54-0.6)	0.19 (0.15-0.22)	1 (0.99-1)	0.52	0.61
Pooled	CAD4TB	48	0.97 (0.92-0.99)	0.61 (0.58-0.64)	0.2 (0.17-0.24)	1 (0.99-1)	0.56	0.64
Pooled	CAD4TB	49	0.96 (0.91-0.99)	0.64 (0.61-0.67)	0.21 (0.18-0.25)	0.99 (0.99-1)	0.59	0.67
Pooled	CAD4TB	50	0.96 (0.91-0.99)	0.67 (0.64-0.7)	0.23 (0.19-0.27)	0.99 (0.99-1)	0.61	0.7
Pooled	CAD4TB	51	0.96 (0.91-0.99)	0.7 (0.67-0.72)	0.24 (0.2-0.28)	0.99 (0.99-1)	0.64	0.72
Pooled	CAD4TB	52	0.96 (0.91-0.99)	0.71 (0.68-0.74)	0.25 (0.21-0.29)	0.99 (0.99-1)	0.65	0.73
Pooled	CAD4TB	53	0.96 (0.91-0.99)	0.73 (0.7-0.76)	0.26 (0.22-0.31)	0.99 (0.99-1)	0.67	0.75
Pooled	CAD4TB	54	0.95 (0.9-0.98)	0.74 (0.71-0.77)	0.27 (0.23-0.32)	0.99 (0.99-1)	0.68	0.76
Pooled	CAD4TB	55	0.95 (0.9-0.98)	0.77 (0.74-0.79)	0.29 (0.24-0.34)	0.99 (0.99-1)	0.7	0.78
Pooled	CAD4TB	56	0.95 (0.9-0.98)	0.78 (0.76-0.81)	0.31 (0.26-0.36)	0.99 (0.99-1)	0.72	0.8
Pooled	CAD4TB	57	0.95 (0.9-0.98)	0.8 (0.77-0.82)	0.32 (0.27-0.38)	0.99 (0.99-1)	0.73	0.81
Pooled	CAD4TB	58	0.94 (0.87-0.97)	0.81 (0.78-0.83)	0.33 (0.28-0.38)	0.99 (0.98-1)	0.74	0.82
Pooled	CAD4TB	59	0.94 (0.87-0.97)	0.82 (0.79-0.84)	0.34 (0.29-0.4)	0.99 (0.98-1)	0.75	0.83
Pooled	CAD4TB	60	0.92 (0.85-0.96)	0.82 (0.8-0.84)	0.34 (0.29-0.4)	0.99 (0.98-1)	0.75	0.83
Pooled	CAD4TB	61	0.92 (0.85-0.96)	0.83 (0.8-0.85)	0.35 (0.29-0.4)	0.99 (0.98-1)	0.76	0.83
Pooled	CAD4TB	62	0.92 (0.85-0.96)	0.83 (0.81-0.86)	0.36 (0.3-0.42)	0.99 (0.98-1)	0.77	0.84
Pooled	CAD4TB	63	0.91 (0.84-0.96)	0.84 (0.82-0.86)	0.36 (0.31-0.42)	0.99 (0.98-0.99)	0.77	0.85
Pooled	CAD4TB	64	0.9 (0.83-0.95)	0.84 (0.82-0.86)	0.37 (0.31-0.43)	0.99 (0.98-0.99)	0.78	0.85

Site	Deep Learning System	Abnormality Score	Sensitivity	Specificity	Positive Predictive Value	Negative Predictive Value	Xpert Saving	Accuracy
Pooled	CAD4TB	65	0.89 (0.82-0.94)	0.85 (0.83-0.87)	0.37 (0.31-0.43)	0.99 (0.98-0.99)	0.78	0.85
Pooled	CAD4TB	66	0.87 (0.79-0.93)	0.85 (0.83-0.87)	0.37 (0.31-0.43)	0.99 (0.98-0.99)	0.79	0.85
Pooled	CAD4TB	67	0.84 (0.76-0.91)	0.86 (0.83-0.88)	0.37 (0.31-0.43)	0.98 (0.97-0.99)	0.79	0.85
Pooled	CAD4TB	68	0.84 (0.76-0.91)	0.86 (0.84-0.88)	0.38 (0.32-0.45)	0.98 (0.97-0.99)	0.8	0.86
Pooled	CAD4TB	69	0.84 (0.76-0.91)	0.87 (0.85-0.89)	0.4 (0.34-0.47)	0.98 (0.97-0.99)	0.81	0.87
Pooled	CAD4TB	70	0.83 (0.75-0.9)	0.88 (0.86-0.9)	0.4 (0.34-0.47)	0.98 (0.97-0.99)	0.81	0.87
Pooled	CAD4TB	71	0.82 (0.73-0.88)	0.88 (0.86-0.9)	0.41 (0.34-0.47)	0.98 (0.97-0.99)	0.82	0.87
Pooled	CAD4TB	72	0.81 (0.72-0.88)	0.88 (0.86-0.9)	0.41 (0.34-0.48)	0.98 (0.97-0.99)	0.82	0.88
Pooled	CAD4TB	73	0.78 (0.69-0.85)	0.89 (0.87-0.91)	0.42 (0.35-0.49)	0.98 (0.96-0.98)	0.83	0.88
Pooled	CAD4TB	74	0.77 (0.68-0.85)	0.9 (0.88-0.92)	0.43 (0.36-0.51)	0.98 (0.96-0.98)	0.84	0.89
Pooled	CAD4TB	75	0.75 (0.66-0.83)	0.91 (0.89-0.92)	0.45 (0.37-0.52)	0.97 (0.96-0.98)	0.85	0.89
Pooled	CAD4TB	76	0.72 (0.63-0.81)	0.91 (0.89-0.93)	0.45 (0.38-0.53)	0.97 (0.96-0.98)	0.85	0.9
Pooled	CAD4TB	77	0.72 (0.63-0.81)	0.91 (0.9-0.93)	0.46 (0.38-0.54)	0.97 (0.96-0.98)	0.86	0.9
Pooled	CAD4TB	78	0.7 (0.6-0.78)	0.92 (0.9-0.93)	0.46 (0.38-0.54)	0.97 (0.96-0.98)	0.86	0.9
Pooled	CAD4TB	79	0.69 (0.59-0.77)	0.92 (0.9-0.94)	0.46 (0.38-0.54)	0.97 (0.95-0.98)	0.86	0.9
Pooled	CAD4TB	80	0.67 (0.57-0.76)	0.93 (0.91-0.94)	0.48 (0.4-0.56)	0.97 (0.95-0.98)	0.87	0.9
Pooled	CAD4TB	81	0.65 (0.55-0.74)	0.93 (0.92-0.95)	0.49 (0.41-0.57)	0.96 (0.95-0.97)	0.88	0.91
Pooled	CAD4TB	82	0.61 (0.52-0.71)	0.94 (0.92-0.95)	0.5 (0.42-0.59)	0.96 (0.95-0.97)	0.89	0.91
Pooled	CAD4TB	83	0.61 (0.52-0.71)	0.94 (0.93-0.96)	0.52 (0.43-0.6)	0.96 (0.95-0.97)	0.89	0.91
Pooled	CAD4TB	84	0.57 (0.47-0.66)	0.95 (0.93-0.96)	0.51 (0.42-0.6)	0.96 (0.94-0.97)	0.9	0.91
Pooled	CAD4TB	85	0.53 (0.43-0.63)	0.95 (0.93-0.96)	0.51 (0.42-0.61)	0.95 (0.94-0.96)	0.91	0.91
Pooled	CAD4TB	86	0.5 (0.41-0.6)	0.95 (0.94-0.97)	0.52 (0.42-0.62)	0.95 (0.94-0.96)	0.91	0.91
Pooled	CAD4TB	87	0.48 (0.38-0.57)	0.96 (0.94-0.97)	0.53 (0.42-0.63)	0.95 (0.93-0.96)	0.92	0.91
Pooled	CAD4TB	88	0.47 (0.37-0.57)	0.96 (0.95-0.97)	0.55 (0.45-0.66)	0.95 (0.93-0.96)	0.92	0.92
Pooled	CAD4TB	89	0.44 (0.35-0.54)	0.96 (0.95-0.97)	0.55 (0.44-0.66)	0.94 (0.93-0.96)	0.93	0.92
Pooled	CAD4TB	90	0.4 (0.31-0.5)	0.97 (0.96-0.98)	0.59 (0.47-0.7)	0.94 (0.93-0.95)	0.94	0.92
Pooled	CAD4TB	91	0.37 (0.28-0.46)	0.97 (0.96-0.98)	0.58 (0.45-0.7)	0.94 (0.92-0.95)	0.94	0.92
Pooled	CAD4TB	92	0.37 (0.28-0.46)	0.98 (0.96-0.98)	0.6 (0.47-0.72)	0.94 (0.92-0.95)	0.94	0.92
Pooled	CAD4TB	93	0.36 (0.27-0.46)	0.98 (0.97-0.99)	0.63 (0.5-0.75)	0.94 (0.92-0.95)	0.95	0.92
Pooled	CAD4TB	94	0.35 (0.26-0.45)	0.98 (0.97-0.99)	0.67 (0.53-0.79)	0.94 (0.92-0.95)	0.95	0.92
Pooled	CAD4TB	95	0.32 (0.23-0.42)	0.98 (0.98-0.99)	0.67 (0.53-0.8)	0.94 (0.92-0.95)	0.96	0.92
Pooled	CAD4TB	96	0.31 (0.23-0.41)	0.99 (0.98-0.99)	0.69 (0.55-0.82)	0.93 (0.92-0.95)	0.96	0.92

Site	Deep Learning System	Abnormality Score	Sensitivity	Specificity	Positive Predictive Value	Negative Predictive Value	Xpert Saving	Accuracy
Pooled	CAD4TB	97	0.28 (0.19-0.37)	0.99 (0.98-0.99)	0.7 (0.54-0.83)	0.93 (0.92-0.95)	0.96	0.92
Pooled	CAD4TB	98	0.28 (0.19-0.37)	0.99 (0.98-0.99)	0.71 (0.55-0.84)	0.93 (0.92-0.95)	0.96	0.92
Pooled	CAD4TB	99	0.28 (0.19-0.37)	0.99 (0.98-0.99)	0.71 (0.55-0.84)	0.93 (0.92-0.95)	0.96	0.92
Pooled	Lunit	0	1 (0.97-1)	0 (0-0)	0.09 (0.08-0.11)	NA	0	0.09
Pooled	Lunit	0.01	1 (0.97-1)	0.22 (0.19-0.24)	0.11 (0.09-0.14)	1 (0.98-1)	0.2	0.09
Pooled	Lunit	0.02	0.99 (0.95-1)	0.35 (0.32-0.38)	0.13 (0.11-0.16)	1 (0.99-1)	0.32	0.09
Pooled	Lunit	0.03	0.99 (0.95-1)	0.4 (0.37-0.43)	0.14 (0.12-0.17)	1 (0.99-1)	0.36	0.1
Pooled	Lunit	0.04	0.99 (0.95-1)	0.43 (0.4-0.46)	0.15 (0.12-0.18)	1 (0.99-1)	0.39	0.12
Pooled	Lunit	0.05	0.99 (0.95-1)	0.45 (0.42-0.48)	0.15 (0.13-0.18)	1 (0.99-1)	0.41	0.15
Pooled	Lunit	0.06	0.99 (0.95-1)	0.47 (0.44-0.5)	0.16 (0.13-0.19)	1 (0.99-1)	0.43	0.19
Pooled	Lunit	0.07	0.99 (0.95-1)	0.49 (0.46-0.52)	0.16 (0.13-0.19)	1 (0.99-1)	0.44	0.23
Pooled	Lunit	0.08	0.99 (0.95-1)	0.51 (0.48-0.54)	0.17 (0.14-0.2)	1 (0.99-1)	0.47	0.28
Pooled	Lunit	0.09	0.99 (0.95-1)	0.53 (0.5-0.56)	0.17 (0.15-0.21)	1 (0.99-1)	0.48	0.32
Pooled	Lunit	0.1	0.99 (0.95-1)	0.54 (0.51-0.57)	0.18 (0.15-0.21)	1 (0.99-1)	0.49	0.35
Pooled	Lunit	0.11	0.99 (0.95-1)	0.56 (0.53-0.59)	0.18 (0.15-0.22)	1 (0.99-1)	0.51	0.39
Pooled	Lunit	0.12	0.99 (0.95-1)	0.57 (0.54-0.6)	0.19 (0.16-0.22)	1 (0.99-1)	0.52	0.42
Pooled	Lunit	0.13	0.99 (0.95-1)	0.59 (0.56-0.62)	0.19 (0.16-0.23)	1 (0.99-1)	0.53	0.46
Pooled	Lunit	0.14	0.98 (0.94-1)	0.6 (0.57-0.63)	0.2 (0.16-0.23)	1 (0.99-1)	0.54	0.5
Pooled	Lunit	0.15	0.98 (0.94-1)	0.61 (0.58-0.63)	0.2 (0.17-0.24)	1 (0.99-1)	0.55	0.53
Pooled	Lunit	0.16	0.98 (0.94-1)	0.62 (0.59-0.65)	0.2 (0.17-0.24)	1 (0.99-1)	0.56	0.55
Pooled	Lunit	0.17	0.98 (0.94-1)	0.62 (0.59-0.65)	0.21 (0.17-0.24)	1 (0.99-1)	0.57	0.57
Pooled	Lunit	0.18	0.98 (0.94-1)	0.63 (0.6-0.66)	0.21 (0.18-0.25)	1 (0.99-1)	0.58	0.59
Pooled	Lunit	0.19	0.98 (0.94-1)	0.64 (0.61-0.67)	0.21 (0.18-0.25)	1 (0.99-1)	0.58	0.61
Pooled	Lunit	0.2	0.98 (0.94-1)	0.64 (0.61-0.67)	0.22 (0.18-0.25)	1 (0.99-1)	0.59	0.63
Pooled	Lunit	0.21	0.98 (0.94-1)	0.65 (0.62-0.68)	0.22 (0.18-0.26)	1 (0.99-1)	0.59	0.64
Pooled	Lunit	0.22	0.98 (0.94-1)	0.66 (0.63-0.69)	0.22 (0.19-0.26)	1 (0.99-1)	0.6	0.66
Pooled	Lunit	0.23	0.98 (0.94-1)	0.66 (0.64-0.69)	0.23 (0.19-0.27)	1 (0.99-1)	0.61	0.67
Pooled	Lunit	0.24	0.97 (0.92-0.99)	0.67 (0.64-0.7)	0.23 (0.19-0.27)	1 (0.99-1)	0.61	0.68
Pooled	Lunit	0.25	0.97 (0.92-0.99)	0.68 (0.65-0.7)	0.23 (0.19-0.27)	1 (0.99-1)	0.62	0.69
Pooled	Lunit	0.26	0.97 (0.92-0.99)	0.68 (0.65-0.71)	0.23 (0.2-0.28)	1 (0.99-1)	0.62	0.7
Pooled	Lunit	0.27	0.97 (0.92-0.99)	0.68 (0.66-0.71)	0.24 (0.2-0.28)	1 (0.99-1)	0.62	0.71
Pooled	Lunit	0.28	0.96 (0.91-0.99)	0.69 (0.66-0.71)	0.23 (0.2-0.28)	0.99 (0.99-1)	0.63	0.71

Site	Deep Learning System	Abnormality Score	Sensitivity	Specificity	Positive Predictive Value	Negative Predictive Value	Xpert Saving	Accuracy
Pooled	Lunit	0.29	0.96 (0.91-0.99)	0.69 (0.66-0.72)	0.24 (0.2-0.28)	0.99 (0.99-1)	0.63	0.72
Pooled	Lunit	0.3	0.96 (0.91-0.99)	0.69 (0.66-0.72)	0.24 (0.2-0.28)	0.99 (0.99-1)	0.63	0.72
Pooled	Lunit	0.31	0.96 (0.91-0.99)	0.69 (0.66-0.72)	0.24 (0.2-0.28)	0.99 (0.99-1)	0.63	0.73
Pooled	Lunit	0.32	0.96 (0.91-0.99)	0.69 (0.66-0.72)	0.24 (0.2-0.28)	0.99 (0.99-1)	0.63	0.74
Pooled	Lunit	0.33	0.96 (0.91-0.99)	0.7 (0.67-0.72)	0.24 (0.2-0.28)	0.99 (0.99-1)	0.64	0.75
Pooled	Lunit	0.34	0.96 (0.91-0.99)	0.7 (0.67-0.73)	0.24 (0.2-0.29)	0.99 (0.99-1)	0.64	0.76
Pooled	Lunit	0.35	0.95 (0.9-0.98)	0.7 (0.68-0.73)	0.24 (0.2-0.29)	0.99 (0.98-1)	0.64	0.77
Pooled	Lunit	0.36	0.95 (0.9-0.98)	0.71 (0.68-0.74)	0.25 (0.21-0.29)	0.99 (0.99-1)	0.65	0.78
Pooled	Lunit	0.37	0.95 (0.9-0.98)	0.71 (0.69-0.74)	0.25 (0.21-0.3)	0.99 (0.99-1)	0.65	0.78
Pooled	Lunit	0.38	0.95 (0.9-0.98)	0.72 (0.69-0.74)	0.25 (0.21-0.3)	0.99 (0.99-1)	0.65	0.79
Pooled	Lunit	0.39	0.95 (0.9-0.98)	0.72 (0.69-0.74)	0.25 (0.21-0.3)	0.99 (0.99-1)	0.66	0.79
Pooled	Lunit	0.4	0.95 (0.9-0.98)	0.72 (0.69-0.75)	0.25 (0.21-0.3)	0.99 (0.99-1)	0.66	0.8
Pooled	Lunit	0.41	0.95 (0.9-0.98)	0.72 (0.69-0.75)	0.25 (0.21-0.3)	0.99 (0.99-1)	0.66	0.8
Pooled	Lunit	0.42	0.95 (0.9-0.98)	0.72 (0.69-0.75)	0.26 (0.21-0.3)	0.99 (0.99-1)	0.66	0.8
Pooled	Lunit	0.43	0.95 (0.9-0.98)	0.72 (0.7-0.75)	0.26 (0.21-0.3)	0.99 (0.99-1)	0.66	0.81
Pooled	Lunit	0.44	0.95 (0.9-0.98)	0.73 (0.7-0.75)	0.26 (0.22-0.3)	0.99 (0.99-1)	0.66	0.81
Pooled	Lunit	0.45	0.95 (0.9-0.98)	0.73 (0.7-0.76)	0.26 (0.22-0.31)	0.99 (0.99-1)	0.67	0.82
Pooled	Lunit	0.46	0.95 (0.9-0.98)	0.74 (0.71-0.76)	0.27 (0.22-0.31)	0.99 (0.99-1)	0.67	0.82
Pooled	Lunit	0.47	0.95 (0.9-0.98)	0.74 (0.71-0.76)	0.27 (0.22-0.31)	0.99 (0.99-1)	0.67	0.83
Pooled	Lunit	0.48	0.95 (0.9-0.98)	0.74 (0.71-0.77)	0.27 (0.23-0.32)	0.99 (0.99-1)	0.68	0.83
Pooled	Lunit	0.49	0.95 (0.9-0.98)	0.74 (0.72-0.77)	0.27 (0.23-0.32)	0.99 (0.99-1)	0.68	0.83
Pooled	Lunit	0.5	0.95 (0.9-0.98)	0.75 (0.72-0.77)	0.27 (0.23-0.32)	0.99 (0.99-1)	0.68	0.83
Pooled	Lunit	0.51	0.95 (0.9-0.98)	0.75 (0.72-0.77)	0.28 (0.23-0.32)	0.99 (0.99-1)	0.68	0.84
Pooled	Lunit	0.52	0.95 (0.9-0.98)	0.75 (0.72-0.78)	0.28 (0.23-0.33)	0.99 (0.99-1)	0.69	0.84
Pooled	Lunit	0.53	0.95 (0.9-0.98)	0.75 (0.72-0.78)	0.28 (0.23-0.33)	0.99 (0.99-1)	0.69	0.84
Pooled	Lunit	0.54	0.95 (0.9-0.98)	0.76 (0.73-0.78)	0.28 (0.24-0.33)	0.99 (0.99-1)	0.69	0.85
Pooled	Lunit	0.55	0.95 (0.9-0.98)	0.76 (0.73-0.78)	0.28 (0.24-0.33)	0.99 (0.99-1)	0.69	0.85
Pooled	Lunit	0.56	0.94 (0.88-0.98)	0.76 (0.73-0.79)	0.28 (0.24-0.33)	0.99 (0.98-1)	0.7	0.85
Pooled	Lunit	0.57	0.94 (0.88-0.98)	0.76 (0.74-0.79)	0.28 (0.24-0.33)	0.99 (0.98-1)	0.7	0.85
Pooled	Lunit	0.58	0.94 (0.88-0.98)	0.77 (0.74-0.79)	0.29 (0.24-0.34)	0.99 (0.98-1)	0.7	0.86
Pooled	Lunit	0.59	0.94 (0.88-0.98)	0.77 (0.74-0.79)	0.29 (0.24-0.34)	0.99 (0.98-1)	0.7	0.86
Pooled	Lunit	0.6	0.94 (0.88-0.98)	0.77 (0.74-0.8)	0.29 (0.25-0.34)	0.99 (0.98-1)	0.71	0.86

Site	Deep Learning System	Abnormality Score	Sensitivity	Specificity	Positive Predictive Value	Negative Predictive Value	Xpert Saving	Accuracy
Pooled	Lunit	0.61	0.94 (0.88-0.98)	0.77 (0.75-0.8)	0.29 (0.25-0.34)	0.99 (0.98-1)	0.71	0.87
Pooled	Lunit	0.62	0.94 (0.88-0.98)	0.77 (0.75-0.8)	0.3 (0.25-0.35)	0.99 (0.98-1)	0.71	0.87
Pooled	Lunit	0.63	0.94 (0.88-0.98)	0.78 (0.75-0.8)	0.3 (0.25-0.35)	0.99 (0.98-1)	0.71	0.87
Pooled	Lunit	0.64	0.94 (0.88-0.98)	0.78 (0.75-0.8)	0.3 (0.25-0.35)	0.99 (0.98-1)	0.71	0.88
Pooled	Lunit	0.65	0.94 (0.88-0.98)	0.78 (0.75-0.8)	0.3 (0.25-0.35)	0.99 (0.98-1)	0.71	0.89
Pooled	Lunit	0.66	0.94 (0.88-0.98)	0.78 (0.75-0.8)	0.3 (0.25-0.35)	0.99 (0.98-1)	0.71	0.89
Pooled	Lunit	0.67	0.94 (0.88-0.98)	0.78 (0.76-0.81)	0.3 (0.26-0.36)	0.99 (0.98-1)	0.72	0.89
Pooled	Lunit	0.68	0.94 (0.88-0.98)	0.78 (0.76-0.81)	0.3 (0.26-0.36)	0.99 (0.98-1)	0.72	0.89
Pooled	Lunit	0.69	0.94 (0.87-0.97)	0.79 (0.76-0.81)	0.3 (0.26-0.36)	0.99 (0.98-1)	0.72	0.9
Pooled	Lunit	0.7	0.93 (0.86-0.97)	0.79 (0.76-0.81)	0.31 (0.26-0.36)	0.99 (0.98-1)	0.72	0.9
Pooled	Lunit	0.71	0.93 (0.86-0.97)	0.79 (0.77-0.81)	0.31 (0.26-0.36)	0.99 (0.98-1)	0.73	0.9
Pooled	Lunit	0.72	0.93 (0.86-0.97)	0.8 (0.77-0.82)	0.31 (0.26-0.37)	0.99 (0.98-1)	0.73	0.9
Pooled	Lunit	0.73	0.93 (0.86-0.97)	0.8 (0.77-0.82)	0.32 (0.27-0.37)	0.99 (0.98-1)	0.73	0.91
Pooled	Lunit	0.74	0.93 (0.86-0.97)	0.8 (0.78-0.82)	0.32 (0.27-0.37)	0.99 (0.98-1)	0.73	0.91
Pooled	Lunit	0.75	0.93 (0.86-0.97)	0.8 (0.78-0.83)	0.32 (0.27-0.37)	0.99 (0.98-1)	0.74	0.91
Pooled	Lunit	0.76	0.93 (0.86-0.97)	0.8 (0.78-0.83)	0.32 (0.27-0.38)	0.99 (0.98-1)	0.74	0.92
Pooled	Lunit	0.77	0.93 (0.86-0.97)	0.81 (0.78-0.83)	0.33 (0.27-0.38)	0.99 (0.98-1)	0.74	0.92
Pooled	Lunit	0.78	0.93 (0.86-0.97)	0.81 (0.79-0.84)	0.33 (0.28-0.39)	0.99 (0.98-1)	0.74	0.92
Pooled	Lunit	0.79	0.93 (0.86-0.97)	0.81 (0.79-0.84)	0.33 (0.28-0.39)	0.99 (0.98-1)	0.75	0.92
Pooled	Lunit	0.8	0.93 (0.86-0.97)	0.82 (0.79-0.84)	0.34 (0.28-0.39)	0.99 (0.98-1)	0.75	0.92
Pooled	Lunit	0.81	0.92 (0.85-0.96)	0.83 (0.8-0.85)	0.35 (0.29-0.4)	0.99 (0.98-1)	0.76	0.93
Pooled	Lunit	0.82	0.92 (0.85-0.96)	0.83 (0.8-0.85)	0.35 (0.29-0.41)	0.99 (0.98-1)	0.76	0.93
Pooled	Lunit	0.83	0.92 (0.85-0.96)	0.83 (0.81-0.86)	0.36 (0.3-0.41)	0.99 (0.98-1)	0.77	0.94
Pooled	Lunit	0.84	0.92 (0.85-0.96)	0.84 (0.82-0.86)	0.36 (0.31-0.42)	0.99 (0.98-1)	0.77	0.94
Pooled	Lunit	0.85	0.92 (0.85-0.96)	0.84 (0.82-0.86)	0.37 (0.31-0.43)	0.99 (0.98-1)	0.77	0.94
Pooled	Lunit	0.86	0.91 (0.84-0.96)	0.85 (0.82-0.87)	0.37 (0.32-0.43)	0.99 (0.98-0.99)	0.78	0.93
Pooled	Lunit	0.87	0.9 (0.83-0.95)	0.85 (0.83-0.87)	0.38 (0.32-0.44)	0.99 (0.98-0.99)	0.78	0.94
Pooled	Lunit	0.88	0.89 (0.82-0.94)	0.86 (0.84-0.88)	0.39 (0.33-0.45)	0.99 (0.98-0.99)	0.79	0.94
Pooled	Lunit	0.89	0.89 (0.82-0.94)	0.86 (0.84-0.88)	0.39 (0.33-0.45)	0.99 (0.98-0.99)	0.79	0.94
Pooled	Lunit	0.9	0.89 (0.82-0.94)	0.87 (0.85-0.89)	0.4 (0.34-0.47)	0.99 (0.98-0.99)	0.8	0.94
Pooled	Lunit	0.91	0.87 (0.79-0.93)	0.87 (0.85-0.89)	0.41 (0.35-0.48)	0.99 (0.98-0.99)	0.81	0.93
Pooled	Lunit	0.92	0.87 (0.79-0.93)	0.89 (0.87-0.91)	0.44 (0.37-0.5)	0.99 (0.98-0.99)	0.82	0.93

Site	Deep Learning System	Abnormality Score	Sensitivity	Specificity	Positive Predictive Value	Negative Predictive Value	Xpert Saving	Accuracy
Pooled	Lunit	0.93	0.85 (0.77-0.91)	0.89 (0.87-0.91)	0.44 (0.37-0.51)	0.98 (0.97-0.99)	0.82	0.92
Pooled	Lunit	0.94	0.82 (0.73-0.88)	0.91 (0.89-0.92)	0.47 (0.4-0.54)	0.98 (0.97-0.99)	0.84	0.92
Pooled	Lunit	0.95	0.82 (0.73-0.88)	0.92 (0.91-0.94)	0.51 (0.44-0.59)	0.98 (0.97-0.99)	0.86	0.92
Pooled	Lunit	0.96	0.78 (0.69-0.85)	0.94 (0.92-0.95)	0.55 (0.47-0.63)	0.98 (0.97-0.99)	0.87	0.91
Pooled	Lunit	0.97	0.69 (0.59-0.77)	0.95 (0.94-0.97)	0.6 (0.51-0.69)	0.97 (0.96-0.98)	0.9	0.91
Pooled	Lunit	0.98	0.58 (0.48-0.67)	0.97 (0.96-0.98)	0.69 (0.59-0.78)	0.96 (0.94-0.97)	0.92	0.91
Pooled	Lunit	0.99	0.31 (0.23-0.41)	0.99 (0.98-1)	0.77 (0.62-0.89)	0.93 (0.92-0.95)	0.96	0.91
Pooled	Lunit	1	0 (0-0.03)	1 (1-1)	NA	0.91 (0.89-0.92)	1	0.09
Pooled	qXR	0.01	1 (0.97-1)	0 (0-0)	0.09 (0.08-0.11)	NA	0	0.29
Pooled	qXR	0.02	1 (0.97-1)	0 (0-0)	0.09 (0.08-0.11)	NA	0	0.41
Pooled	qXR	0.03	1 (0.97-1)	0 (0-0.01)	0.09 (0.08-0.11)	1 (0.16-1)	0	0.45
Pooled	qXR	0.04	1 (0.97-1)	0.01 (0.01-0.02)	0.09 (0.08-0.11)	1 (0.72-1)	0.01	0.48
Pooled	qXR	0.05	1 (0.97-1)	0.03 (0.02-0.04)	0.09 (0.08-0.11)	1 (0.9-1)	0.03	0.5
Pooled	qXR	0.06	1 (0.97-1)	0.06 (0.05-0.08)	0.1 (0.08-0.12)	1 (0.95-1)	0.06	0.52
Pooled	qXR	0.07	1 (0.97-1)	0.11 (0.09-0.13)	0.1 (0.08-0.12)	1 (0.97-1)	0.1	0.53
Pooled	qXR	0.08	1 (0.97-1)	0.16 (0.14-0.18)	0.11 (0.09-0.13)	1 (0.98-1)	0.14	0.56
Pooled	qXR	0.09	1 (0.97-1)	0.21 (0.19-0.24)	0.11 (0.09-0.13)	1 (0.98-1)	0.19	0.57
Pooled	qXR	0.1	1 (0.97-1)	0.25 (0.23-0.28)	0.12 (0.1-0.14)	1 (0.99-1)	0.23	0.58
Pooled	qXR	0.11	1 (0.97-1)	0.29 (0.26-0.31)	0.12 (0.1-0.15)	1 (0.99-1)	0.26	0.6
Pooled	qXR	0.12	1 (0.97-1)	0.32 (0.3-0.35)	0.13 (0.11-0.15)	1 (0.99-1)	0.3	0.61
Pooled	qXR	0.13	0.98 (0.94-1)	0.37 (0.34-0.39)	0.13 (0.11-0.16)	0.99 (0.98-1)	0.33	0.62
Pooled	qXR	0.14	0.98 (0.94-1)	0.41 (0.38-0.44)	0.14 (0.12-0.17)	1 (0.98-1)	0.38	0.63
Pooled	qXR	0.15	0.98 (0.94-1)	0.45 (0.42-0.48)	0.15 (0.13-0.18)	1 (0.99-1)	0.41	0.64
Pooled	qXR	0.16	0.97 (0.92-0.99)	0.48 (0.45-0.51)	0.16 (0.13-0.19)	0.99 (0.98-1)	0.44	0.65
Pooled	qXR	0.17	0.97 (0.92-0.99)	0.5 (0.47-0.53)	0.16 (0.14-0.19)	0.99 (0.98-1)	0.46	0.66
Pooled	qXR	0.18	0.97 (0.92-0.99)	0.53 (0.5-0.56)	0.17 (0.14-0.2)	0.99 (0.98-1)	0.48	0.66
Pooled	qXR	0.19	0.97 (0.92-0.99)	0.55 (0.52-0.58)	0.18 (0.15-0.21)	1 (0.99-1)	0.51	0.67
Pooled	qXR	0.2	0.97 (0.92-0.99)	0.58 (0.55-0.61)	0.19 (0.16-0.22)	1 (0.99-1)	0.53	0.67
Pooled	qXR	0.21	0.97 (0.92-0.99)	0.59 (0.56-0.62)	0.19 (0.16-0.23)	1 (0.99-1)	0.54	0.68
Pooled	qXR	0.22	0.97 (0.92-0.99)	0.61 (0.58-0.64)	0.2 (0.17-0.24)	1 (0.99-1)	0.56	0.69
Pooled	qXR	0.23	0.97 (0.92-0.99)	0.63 (0.6-0.65)	0.21 (0.17-0.24)	1 (0.99-1)	0.57	0.69
Pooled	qXR	0.24	0.97 (0.92-0.99)	0.64 (0.61-0.67)	0.21 (0.18-0.25)	1 (0.99-1)	0.59	0.7

Site	Deep Learning System	Abnormality Score	Sensitivity	Specificity	Positive Predictive Value	Negative Predictive Value	Xpert Saving	Accuracy
Pooled	qXR	0.25	0.97 (0.92-0.99)	0.65 (0.62-0.68)	0.22 (0.18-0.26)	1 (0.99-1)	0.6	0.7
Pooled	qXR	0.26	0.96 (0.91-0.99)	0.66 (0.63-0.69)	0.22 (0.19-0.26)	0.99 (0.99-1)	0.61	0.71
Pooled	qXR	0.27	0.96 (0.91-0.99)	0.67 (0.64-0.7)	0.23 (0.19-0.27)	0.99 (0.99-1)	0.61	0.71
Pooled	qXR	0.28	0.96 (0.91-0.99)	0.68 (0.65-0.71)	0.23 (0.19-0.27)	0.99 (0.99-1)	0.62	0.71
Pooled	qXR	0.29	0.96 (0.91-0.99)	0.69 (0.66-0.71)	0.24 (0.2-0.28)	0.99 (0.99-1)	0.63	0.71
Pooled	qXR	0.3	0.96 (0.91-0.99)	0.69 (0.67-0.72)	0.24 (0.2-0.28)	0.99 (0.99-1)	0.63	0.71
Pooled	qXR	0.31	0.96 (0.91-0.99)	0.7 (0.67-0.73)	0.24 (0.2-0.29)	0.99 (0.99-1)	0.64	0.71
Pooled	qXR	0.32	0.96 (0.91-0.99)	0.71 (0.68-0.74)	0.25 (0.21-0.29)	0.99 (0.99-1)	0.65	0.72
Pooled	qXR	0.33	0.96 (0.91-0.99)	0.72 (0.69-0.75)	0.26 (0.22-0.3)	0.99 (0.99-1)	0.66	0.72
Pooled	qXR	0.34	0.96 (0.91-0.99)	0.73 (0.7-0.76)	0.26 (0.22-0.31)	0.99 (0.99-1)	0.67	0.72
Pooled	qXR	0.35	0.96 (0.91-0.99)	0.74 (0.71-0.76)	0.27 (0.23-0.32)	1 (0.99-1)	0.67	0.73
Pooled	qXR	0.36	0.96 (0.91-0.99)	0.75 (0.72-0.77)	0.28 (0.23-0.33)	1 (0.99-1)	0.68	0.73
Pooled	qXR	0.37	0.96 (0.91-0.99)	0.76 (0.73-0.78)	0.29 (0.24-0.33)	1 (0.99-1)	0.69	0.74
Pooled	qXR	0.38	0.96 (0.91-0.99)	0.76 (0.74-0.79)	0.29 (0.24-0.34)	1 (0.99-1)	0.7	0.74
Pooled	qXR	0.39	0.96 (0.91-0.99)	0.77 (0.74-0.79)	0.3 (0.25-0.35)	1 (0.99-1)	0.7	0.74
Pooled	qXR	0.4	0.96 (0.91-0.99)	0.77 (0.75-0.8)	0.3 (0.25-0.35)	1 (0.99-1)	0.71	0.74
Pooled	qXR	0.41	0.96 (0.91-0.99)	0.78 (0.76-0.81)	0.31 (0.26-0.36)	1 (0.99-1)	0.71	0.74
Pooled	qXR	0.42	0.96 (0.91-0.99)	0.79 (0.76-0.81)	0.31 (0.26-0.36)	1 (0.99-1)	0.72	0.74
Pooled	qXR	0.43	0.95 (0.9-0.98)	0.79 (0.76-0.81)	0.31 (0.26-0.37)	0.99 (0.99-1)	0.72	0.74
Pooled	qXR	0.44	0.95 (0.9-0.98)	0.79 (0.77-0.82)	0.32 (0.27-0.37)	0.99 (0.99-1)	0.73	0.75
Pooled	qXR	0.45	0.95 (0.9-0.98)	0.8 (0.77-0.82)	0.32 (0.27-0.38)	0.99 (0.99-1)	0.73	0.75
Pooled	qXR	0.46	0.95 (0.9-0.98)	0.8 (0.78-0.83)	0.33 (0.27-0.38)	0.99 (0.99-1)	0.73	0.76
Pooled	qXR	0.47	0.95 (0.9-0.98)	0.81 (0.78-0.83)	0.33 (0.28-0.39)	0.99 (0.99-1)	0.74	0.76
Pooled	qXR	0.48	0.95 (0.9-0.98)	0.81 (0.79-0.84)	0.34 (0.29-0.39)	0.99 (0.99-1)	0.74	0.76
Pooled	qXR	0.49	0.95 (0.9-0.98)	0.82 (0.79-0.84)	0.34 (0.29-0.4)	0.99 (0.99-1)	0.74	0.76
Pooled	qXR	0.5	0.93 (0.86-0.97)	0.82 (0.8-0.84)	0.34 (0.29-0.4)	0.99 (0.98-1)	0.75	0.77
Pooled	qXR	0.51	0.93 (0.86-0.97)	0.82 (0.8-0.85)	0.34 (0.29-0.4)	0.99 (0.98-1)	0.76	0.77
Pooled	qXR	0.52	0.93 (0.86-0.97)	0.83 (0.8-0.85)	0.35 (0.29-0.41)	0.99 (0.98-1)	0.76	0.77
Pooled	qXR	0.53	0.92 (0.85-0.96)	0.83 (0.81-0.85)	0.35 (0.29-0.41)	0.99 (0.98-1)	0.76	0.77
Pooled	qXR	0.54	0.92 (0.85-0.96)	0.83 (0.81-0.86)	0.36 (0.3-0.41)	0.99 (0.98-1)	0.77	0.77
Pooled	qXR	0.55	0.92 (0.85-0.96)	0.84 (0.82-0.86)	0.36 (0.31-0.42)	0.99 (0.98-1)	0.77	0.77
Pooled	qXR	0.56	0.92 (0.85-0.96)	0.84 (0.82-0.86)	0.36 (0.31-0.43)	0.99 (0.98-1)	0.77	0.78

Site	Deep Learning System	Abnormality Score	Sensitivity	Specificity	Positive Predictive Value	Negative Predictive Value	Xpert Saving	Accuracy
Pooled	qXR	0.57	0.92 (0.85-0.96)	0.84 (0.82-0.86)	0.37 (0.31-0.43)	0.99 (0.98-1)	0.77	0.78
Pooled	qXR	0.58	0.92 (0.85-0.96)	0.85 (0.82-0.87)	0.37 (0.32-0.44)	0.99 (0.98-1)	0.78	0.78
Pooled	qXR	0.59	0.92 (0.85-0.96)	0.85 (0.83-0.87)	0.38 (0.33-0.45)	0.99 (0.98-1)	0.78	0.78
Pooled	qXR	0.6	0.92 (0.85-0.96)	0.86 (0.83-0.88)	0.39 (0.33-0.45)	0.99 (0.98-1)	0.79	0.79
Pooled	qXR	0.61	0.91 (0.84-0.96)	0.86 (0.84-0.88)	0.39 (0.33-0.45)	0.99 (0.98-0.99)	0.79	0.79
Pooled	qXR	0.62	0.91 (0.84-0.96)	0.86 (0.84-0.88)	0.4 (0.34-0.46)	0.99 (0.98-0.99)	0.79	0.79
Pooled	qXR	0.63	0.91 (0.84-0.96)	0.87 (0.84-0.89)	0.41 (0.34-0.47)	0.99 (0.98-0.99)	0.8	0.79
Pooled	qXR	0.64	0.9 (0.83-0.95)	0.87 (0.85-0.89)	0.41 (0.35-0.48)	0.99 (0.98-0.99)	0.8	0.79
Pooled	qXR	0.65	0.9 (0.83-0.95)	0.88 (0.86-0.9)	0.43 (0.36-0.49)	0.99 (0.98-0.99)	0.81	0.79
Pooled	qXR	0.66	0.9 (0.83-0.95)	0.89 (0.87-0.91)	0.44 (0.38-0.51)	0.99 (0.98-0.99)	0.82	0.8
Pooled	qXR	0.67	0.88 (0.8-0.93)	0.89 (0.87-0.91)	0.44 (0.38-0.51)	0.99 (0.98-0.99)	0.82	0.8
Pooled	qXR	0.68	0.88 (0.8-0.93)	0.89 (0.87-0.91)	0.45 (0.38-0.52)	0.99 (0.98-0.99)	0.82	0.8
Pooled	qXR	0.69	0.88 (0.8-0.93)	0.89 (0.87-0.91)	0.45 (0.39-0.52)	0.99 (0.98-0.99)	0.82	0.8
Pooled	qXR	0.7	0.87 (0.79-0.93)	0.9 (0.88-0.92)	0.47 (0.4-0.54)	0.99 (0.98-0.99)	0.83	0.8
Pooled	qXR	0.71	0.86 (0.78-0.92)	0.9 (0.88-0.92)	0.47 (0.4-0.54)	0.98 (0.98-0.99)	0.83	0.8
Pooled	qXR	0.72	0.84 (0.76-0.91)	0.91 (0.89-0.93)	0.48 (0.41-0.56)	0.98 (0.97-0.99)	0.84	0.81
Pooled	qXR	0.73	0.82 (0.73-0.88)	0.91 (0.89-0.93)	0.48 (0.41-0.56)	0.98 (0.97-0.99)	0.85	0.81
Pooled	qXR	0.74	0.82 (0.73-0.88)	0.92 (0.9-0.93)	0.49 (0.42-0.57)	0.98 (0.97-0.99)	0.85	0.81
Pooled	qXR	0.75	0.82 (0.73-0.88)	0.92 (0.9-0.94)	0.51 (0.43-0.58)	0.98 (0.97-0.99)	0.85	0.81
Pooled	qXR	0.76	0.82 (0.73-0.88)	0.92 (0.91-0.94)	0.52 (0.44-0.6)	0.98 (0.97-0.99)	0.86	0.82
Pooled	qXR	0.77	0.8 (0.71-0.87)	0.93 (0.91-0.94)	0.53 (0.45-0.61)	0.98 (0.97-0.99)	0.86	0.82
Pooled	qXR	0.78	0.78 (0.69-0.85)	0.93 (0.92-0.95)	0.54 (0.46-0.62)	0.98 (0.97-0.99)	0.87	0.82
Pooled	qXR	0.79	0.77 (0.68-0.85)	0.93 (0.92-0.95)	0.54 (0.46-0.62)	0.98 (0.96-0.98)	0.87	0.82
Pooled	qXR	0.8	0.75 (0.66-0.83)	0.94 (0.92-0.95)	0.54 (0.46-0.62)	0.97 (0.96-0.98)	0.87	0.83
Pooled	qXR	0.81	0.75 (0.66-0.83)	0.94 (0.93-0.95)	0.56 (0.48-0.64)	0.97 (0.96-0.98)	0.88	0.83
Pooled	qXR	0.82	0.74 (0.65-0.82)	0.95 (0.93-0.96)	0.59 (0.5-0.67)	0.97 (0.96-0.98)	0.89	0.84
Pooled	qXR	0.83	0.72 (0.63-0.81)	0.95 (0.94-0.96)	0.6 (0.51-0.69)	0.97 (0.96-0.98)	0.89	0.84
Pooled	qXR	0.84	0.71 (0.61-0.79)	0.96 (0.94-0.97)	0.63 (0.54-0.72)	0.97 (0.96-0.98)	0.9	0.85
Pooled	qXR	0.85	0.67 (0.57-0.76)	0.96 (0.95-0.97)	0.64 (0.55-0.73)	0.97 (0.95-0.98)	0.9	0.85
Pooled	qXR	0.86	0.65 (0.55-0.74)	0.97 (0.95-0.98)	0.65 (0.55-0.74)	0.97 (0.95-0.98)	0.91	0.85
Pooled	qXR	0.87	0.61 (0.51-0.7)	0.97 (0.96-0.98)	0.65 (0.55-0.75)	0.96 (0.95-0.97)	0.92	0.85
Pooled	qXR	0.88	0.61 (0.51-0.7)	0.97 (0.96-0.98)	0.66 (0.56-0.75)	0.96 (0.95-0.97)	0.92	0.86

Site	Deep Learning System	Abnormality Score	Sensitivity	Specificity	Positive Predictive Value	Negative Predictive Value	Xpert Saving	Accuracy
Pooled	qXR	0.89	0.57 (0.47-0.66)	0.97 (0.96-0.98)	0.68 (0.58-0.78)	0.96 (0.94-0.97)	0.92	0.86
Pooled	qXR	0.9	0.52 (0.43-0.62)	0.98 (0.97-0.99)	0.71 (0.6-0.81)	0.95 (0.94-0.97)	0.93	0.87
Pooled	qXR	0.91	0.47 (0.37-0.57)	0.98 (0.98-0.99)	0.75 (0.63-0.85)	0.95 (0.93-0.96)	0.94	0.87
Pooled	qXR	0.92	0.4 (0.31-0.5)	0.98 (0.98-0.99)	0.72 (0.59-0.83)	0.94 (0.93-0.96)	0.95	0.89
Pooled	qXR	0.93	0.33 (0.24-0.43)	0.99 (0.98-0.99)	0.77 (0.62-0.88)	0.94 (0.92-0.95)	0.96	0.89
Pooled	qXR	0.94	0.23 (0.15-0.32)	0.99 (0.99-1)	0.78 (0.6-0.91)	0.93 (0.91-0.94)	0.97	0.9
Pooled	qXR	0.95	0.13 (0.07-0.21)	0.99 (0.99-1)	0.7 (0.46-0.88)	0.92 (0.9-0.93)	0.98	0.91
Pooled	qXR	0.96	0.08 (0.04-0.15)	1 (0.99-1)	0.9 (0.55-1)	0.92 (0.9-0.93)	0.99	0.92
Pooled	qXR	0.97	0.03 (0.01-0.08)	1 (0.99-1)	0.75 (0.19-0.99)	0.91 (0.89-0.93)	1	0.93
Pooled	qXR	0.98	0 (0-0.03)	1 (1-1)	NA	0.91 (0.89-0.92)	1	0.94
Pooled	qXR	0.99	0 (0-0.03)	1 (1-1)	NA	0.91 (0.89-0.92)	1	0.93
Pooled	qXR	1	0 (0-0.03)	1 (1-1)	NA	0.91 (0.89-0.92)	1	0.91
Nepal	CAD4TB	1	1 (0.96-1)	0 (0-0.01)	0.18 (0.15-0.22)	NA	0	0.18
Nepal	CAD4TB	2	1 (0.96-1)	0.01 (0-0.02)	0.18 (0.15-0.22)	1 (0.29-1)	0.01	0.19
Nepal	CAD4TB	3	1 (0.96-1)	0.02 (0.01-0.04)	0.19 (0.15-0.22)	1 (0.63-1)	0.02	0.2
Nepal	CAD4TB	4	1 (0.96-1)	0.04 (0.02-0.06)	0.19 (0.15-0.23)	1 (0.78-1)	0.03	0.21
Nepal	CAD4TB	5	1 (0.96-1)	0.06 (0.04-0.08)	0.19 (0.16-0.23)	1 (0.86-1)	0.05	0.23
Nepal	CAD4TB	6	1 (0.96-1)	0.08 (0.05-0.11)	0.2 (0.16-0.23)	1 (0.89-1)	0.06	0.25
Nepal	CAD4TB	7	1 (0.96-1)	0.1 (0.07-0.13)	0.2 (0.16-0.24)	1 (0.91-1)	0.08	0.26
Nepal	CAD4TB	8	1 (0.96-1)	0.12 (0.09-0.15)	0.2 (0.17-0.24)	1 (0.93-1)	0.1	0.28
Nepal	CAD4TB	9	1 (0.96-1)	0.13 (0.1-0.16)	0.2 (0.17-0.24)	1 (0.93-1)	0.1	0.29
Nepal	CAD4TB	10	0.99 (0.94-1)	0.13 (0.1-0.17)	0.2 (0.17-0.24)	0.98 (0.91-1)	0.11	0.29
Nepal	CAD4TB	11	0.99 (0.94-1)	0.15 (0.11-0.18)	0.21 (0.17-0.25)	0.98 (0.91-1)	0.12	0.3
Nepal	CAD4TB	12	0.99 (0.94-1)	0.16 (0.13-0.2)	0.21 (0.17-0.25)	0.99 (0.92-1)	0.13	0.31
Nepal	CAD4TB	13	0.99 (0.94-1)	0.17 (0.13-0.21)	0.21 (0.17-0.25)	0.99 (0.93-1)	0.14	0.32
Nepal	CAD4TB	14	0.99 (0.94-1)	0.18 (0.14-0.22)	0.21 (0.17-0.25)	0.99 (0.93-1)	0.15	0.32
Nepal	CAD4TB	15	0.99 (0.94-1)	0.19 (0.15-0.23)	0.21 (0.18-0.25)	0.99 (0.93-1)	0.15	0.33
Nepal	CAD4TB	16	0.99 (0.94-1)	0.2 (0.16-0.24)	0.22 (0.18-0.26)	0.99 (0.94-1)	0.16	0.34
Nepal	CAD4TB	17	0.99 (0.94-1)	0.2 (0.16-0.24)	0.22 (0.18-0.26)	0.99 (0.94-1)	0.17	0.35
Nepal	CAD4TB	18	0.99 (0.94-1)	0.21 (0.17-0.25)	0.22 (0.18-0.26)	0.99 (0.94-1)	0.17	0.35
Nepal	CAD4TB	19	0.99 (0.94-1)	0.22 (0.18-0.26)	0.22 (0.18-0.26)	0.99 (0.94-1)	0.18	0.36
Nepal	CAD4TB	20	0.99 (0.94-1)	0.23 (0.19-0.27)	0.22 (0.18-0.26)	0.99 (0.94-1)	0.19	0.37

Site	Deep Learning System	Abnormality Score	Sensitivity	Specificity	Positive Predictive Value	Negative Predictive Value	Xpert Saving	Accuracy
Nepal	CAD4TB	21	0.99 (0.94-1)	0.23 (0.19-0.27)	0.22 (0.18-0.26)	0.99 (0.94-1)	0.19	0.37
Nepal	CAD4TB	22	0.99 (0.94-1)	0.23 (0.19-0.27)	0.22 (0.18-0.27)	0.99 (0.94-1)	0.19	0.37
Nepal	CAD4TB	23	0.99 (0.94-1)	0.24 (0.2-0.28)	0.23 (0.19-0.27)	0.99 (0.95-1)	0.2	0.38
Nepal	CAD4TB	24	0.99 (0.94-1)	0.25 (0.21-0.29)	0.23 (0.19-0.27)	0.99 (0.95-1)	0.2	0.38
Nepal	CAD4TB	25	0.99 (0.94-1)	0.25 (0.21-0.29)	0.23 (0.19-0.27)	0.99 (0.95-1)	0.21	0.38
Nepal	CAD4TB	26	0.99 (0.94-1)	0.25 (0.21-0.3)	0.23 (0.19-0.27)	0.99 (0.95-1)	0.21	0.39
Nepal	CAD4TB	27	0.99 (0.94-1)	0.26 (0.22-0.3)	0.23 (0.19-0.27)	0.99 (0.95-1)	0.21	0.39
Nepal	CAD4TB	28	0.99 (0.94-1)	0.26 (0.22-0.3)	0.23 (0.19-0.27)	0.99 (0.95-1)	0.21	0.39
Nepal	CAD4TB	29	0.99 (0.94-1)	0.27 (0.23-0.31)	0.23 (0.19-0.28)	0.99 (0.95-1)	0.22	0.4
Nepal	CAD4TB	30	0.99 (0.94-1)	0.27 (0.23-0.31)	0.23 (0.19-0.28)	0.99 (0.95-1)	0.22	0.4
Nepal	CAD4TB	31	0.99 (0.94-1)	0.27 (0.23-0.32)	0.23 (0.19-0.28)	0.99 (0.95-1)	0.22	0.4
Nepal	CAD4TB	32	0.99 (0.94-1)	0.28 (0.23-0.32)	0.23 (0.19-0.28)	0.99 (0.95-1)	0.23	0.41
Nepal	CAD4TB	33	0.99 (0.94-1)	0.28 (0.24-0.32)	0.23 (0.19-0.28)	0.99 (0.95-1)	0.23	0.41
Nepal	CAD4TB	34	0.99 (0.94-1)	0.28 (0.24-0.32)	0.23 (0.19-0.28)	0.99 (0.95-1)	0.23	0.41
Nepal	CAD4TB	35	0.99 (0.94-1)	0.28 (0.24-0.33)	0.23 (0.19-0.28)	0.99 (0.95-1)	0.23	0.41
Nepal	CAD4TB	36	0.99 (0.94-1)	0.29 (0.24-0.33)	0.24 (0.19-0.28)	0.99 (0.95-1)	0.23	0.41
Nepal	CAD4TB	37	0.99 (0.94-1)	0.29 (0.25-0.34)	0.24 (0.2-0.28)	0.99 (0.96-1)	0.24	0.42
Nepal	CAD4TB	38	0.99 (0.94-1)	0.29 (0.25-0.34)	0.24 (0.2-0.28)	0.99 (0.96-1)	0.24	0.42
Nepal	CAD4TB	39	0.99 (0.94-1)	0.29 (0.25-0.34)	0.24 (0.2-0.28)	0.99 (0.96-1)	0.24	0.42
Nepal	CAD4TB	40	0.99 (0.94-1)	0.3 (0.25-0.34)	0.24 (0.2-0.28)	0.99 (0.96-1)	0.24	0.42
Nepal	CAD4TB	41	0.99 (0.94-1)	0.3 (0.26-0.35)	0.24 (0.2-0.29)	0.99 (0.96-1)	0.25	0.43
Nepal	CAD4TB	42	0.99 (0.94-1)	0.31 (0.26-0.36)	0.24 (0.2-0.29)	0.99 (0.96-1)	0.25	0.43
Nepal	CAD4TB	43	0.99 (0.94-1)	0.31 (0.27-0.36)	0.24 (0.2-0.29)	0.99 (0.96-1)	0.26	0.43
Nepal	CAD4TB	44	0.99 (0.94-1)	0.33 (0.29-0.38)	0.25 (0.21-0.3)	0.99 (0.96-1)	0.28	0.45
Nepal	CAD4TB	45	0.99 (0.94-1)	0.36 (0.32-0.41)	0.26 (0.21-0.31)	0.99 (0.96-1)	0.3	0.48
Nepal	CAD4TB	46	0.99 (0.94-1)	0.4 (0.35-0.45)	0.27 (0.22-0.32)	0.99 (0.97-1)	0.33	0.5
Nepal	CAD4TB	47	0.98 (0.93-1)	0.45 (0.4-0.5)	0.28 (0.23-0.34)	0.99 (0.96-1)	0.37	0.54
Nepal	CAD4TB	48	0.98 (0.93-1)	0.48 (0.43-0.53)	0.29 (0.24-0.35)	0.99 (0.96-1)	0.39	0.57
Nepal	CAD4TB	49	0.98 (0.93-1)	0.5 (0.45-0.55)	0.3 (0.25-0.36)	0.99 (0.97-1)	0.41	0.59
Nepal	CAD4TB	50	0.98 (0.93-1)	0.53 (0.48-0.58)	0.32 (0.26-0.37)	0.99 (0.97-1)	0.43	0.61
Nepal	CAD4TB	51	0.98 (0.93-1)	0.54 (0.5-0.59)	0.32 (0.27-0.38)	0.99 (0.97-1)	0.45	0.62
Nepal	CAD4TB	52	0.98 (0.93-1)	0.56 (0.51-0.61)	0.33 (0.28-0.39)	0.99 (0.97-1)	0.46	0.63

Site	Deep Learning System	Abnormality Score	Sensitivity	Specificity	Positive Predictive Value	Negative Predictive Value	Xpert Saving	Accuracy
Nepal	CAD4TB	53	0.98 (0.93-1)	0.58 (0.53-0.63)	0.34 (0.29-0.4)	0.99 (0.97-1)	0.48	0.65
Nepal	CAD4TB	54	0.98 (0.93-1)	0.59 (0.54-0.64)	0.35 (0.29-0.41)	0.99 (0.97-1)	0.49	0.66
Nepal	CAD4TB	55	0.98 (0.93-1)	0.61 (0.56-0.66)	0.36 (0.3-0.42)	0.99 (0.97-1)	0.5	0.68
Nepal	CAD4TB	56	0.98 (0.93-1)	0.62 (0.58-0.67)	0.37 (0.31-0.43)	0.99 (0.97-1)	0.51	0.69
Nepal	CAD4TB	57	0.98 (0.93-1)	0.64 (0.59-0.69)	0.38 (0.32-0.44)	0.99 (0.97-1)	0.53	0.7
Nepal	CAD4TB	58	0.97 (0.91-0.99)	0.65 (0.6-0.69)	0.38 (0.32-0.45)	0.99 (0.97-1)	0.54	0.71
Nepal	CAD4TB	59	0.97 (0.91-0.99)	0.66 (0.61-0.7)	0.39 (0.32-0.45)	0.99 (0.97-1)	0.54	0.71
Nepal	CAD4TB	60	0.96 (0.89-0.99)	0.67 (0.62-0.71)	0.39 (0.33-0.46)	0.99 (0.96-1)	0.55	0.72
Nepal	CAD4TB	61	0.96 (0.89-0.99)	0.67 (0.62-0.71)	0.39 (0.33-0.46)	0.99 (0.96-1)	0.56	0.72
Nepal	CAD4TB	62	0.96 (0.89-0.99)	0.69 (0.64-0.73)	0.41 (0.34-0.47)	0.99 (0.97-1)	0.57	0.74
Nepal	CAD4TB	63	0.95 (0.88-0.98)	0.69 (0.65-0.74)	0.41 (0.34-0.48)	0.98 (0.96-0.99)	0.58	0.74
Nepal	CAD4TB	64	0.94 (0.87-0.98)	0.7 (0.65-0.74)	0.41 (0.34-0.48)	0.98 (0.96-0.99)	0.58	0.74
Nepal	CAD4TB	65	0.93 (0.85-0.97)	0.71 (0.66-0.75)	0.41 (0.35-0.48)	0.98 (0.95-0.99)	0.59	0.75
Nepal	CAD4TB	66	0.9 (0.83-0.96)	0.71 (0.67-0.76)	0.41 (0.34-0.48)	0.97 (0.95-0.99)	0.6	0.75
Nepal	CAD4TB	67	0.88 (0.8-0.94)	0.71 (0.67-0.76)	0.41 (0.34-0.48)	0.96 (0.94-0.98)	0.61	0.75
Nepal	CAD4TB	68	0.88 (0.8-0.94)	0.73 (0.68-0.77)	0.42 (0.35-0.49)	0.97 (0.94-0.98)	0.62	0.76
Nepal	CAD4TB	69	0.88 (0.8-0.94)	0.75 (0.7-0.79)	0.44 (0.37-0.51)	0.97 (0.94-0.98)	0.63	0.77
Nepal	CAD4TB	70	0.87 (0.79-0.93)	0.75 (0.71-0.79)	0.44 (0.37-0.52)	0.96 (0.94-0.98)	0.64	0.77
Nepal	CAD4TB	71	0.85 (0.76-0.92)	0.76 (0.72-0.8)	0.44 (0.37-0.52)	0.96 (0.93-0.98)	0.65	0.78
Nepal	CAD4TB	72	0.84 (0.75-0.91)	0.77 (0.73-0.81)	0.45 (0.37-0.53)	0.96 (0.93-0.98)	0.66	0.78
Nepal	CAD4TB	73	0.81 (0.71-0.88)	0.78 (0.73-0.82)	0.45 (0.37-0.53)	0.95 (0.92-0.97)	0.67	0.78
Nepal	CAD4TB	74	0.8 (0.7-0.87)	0.79 (0.74-0.82)	0.45 (0.38-0.53)	0.95 (0.92-0.97)	0.68	0.79
Nepal	CAD4TB	75	0.78 (0.68-0.86)	0.81 (0.76-0.84)	0.47 (0.39-0.55)	0.94 (0.91-0.96)	0.7	0.8
Nepal	CAD4TB	76	0.76 (0.66-0.84)	0.81 (0.77-0.85)	0.48 (0.39-0.56)	0.94 (0.91-0.96)	0.71	0.8
Nepal	CAD4TB	77	0.76 (0.66-0.84)	0.82 (0.78-0.86)	0.48 (0.4-0.57)	0.94 (0.91-0.96)	0.71	0.81
Nepal	CAD4TB	78	0.73 (0.63-0.82)	0.83 (0.79-0.86)	0.49 (0.4-0.57)	0.93 (0.9-0.96)	0.72	0.81
Nepal	CAD4TB	79	0.72 (0.62-0.81)	0.83 (0.79-0.87)	0.49 (0.4-0.58)	0.93 (0.9-0.95)	0.73	0.81
Nepal	CAD4TB	80	0.7 (0.6-0.79)	0.85 (0.81-0.88)	0.51 (0.42-0.6)	0.93 (0.9-0.95)	0.75	0.82
Nepal	CAD4TB	81	0.68 (0.58-0.77)	0.86 (0.82-0.89)	0.51 (0.42-0.6)	0.92 (0.89-0.95)	0.76	0.82
Nepal	CAD4TB	82	0.64 (0.53-0.73)	0.87 (0.83-0.9)	0.52 (0.43-0.62)	0.92 (0.88-0.94)	0.78	0.83
Nepal	CAD4TB	83	0.64 (0.53-0.73)	0.87 (0.84-0.9)	0.53 (0.43-0.62)	0.92 (0.88-0.94)	0.78	0.83
Nepal	CAD4TB	84	0.59 (0.48-0.69)	0.88 (0.84-0.91)	0.51 (0.42-0.61)	0.9 (0.87-0.93)	0.79	0.82

Site	Deep Learning System	Abnormality Score	Sensitivity	Specificity	Positive Predictive Value	Negative Predictive Value	Xpert Saving	Accuracy
Nepal	CAD4TB	85	0.55 (0.45-0.66)	0.88 (0.85-0.91)	0.51 (0.41-0.61)	0.9 (0.87-0.93)	0.8	0.82
Nepal	CAD4TB	86	0.52 (0.42-0.63)	0.89 (0.86-0.92)	0.52 (0.41-0.62)	0.89 (0.86-0.92)	0.82	0.82
Nepal	CAD4TB	87	0.49 (0.38-0.59)	0.9 (0.86-0.92)	0.51 (0.4-0.62)	0.89 (0.85-0.92)	0.83	0.82
Nepal	CAD4TB	88	0.48 (0.37-0.58)	0.91 (0.88-0.94)	0.54 (0.43-0.65)	0.89 (0.85-0.91)	0.84	0.83
Nepal	CAD4TB	89	0.45 (0.34-0.55)	0.91 (0.88-0.94)	0.54 (0.42-0.65)	0.88 (0.85-0.91)	0.85	0.83
Nepal	CAD4TB	90	0.4 (0.3-0.51)	0.93 (0.9-0.95)	0.56 (0.43-0.68)	0.87 (0.84-0.9)	0.87	0.83
Nepal	CAD4TB	91	0.36 (0.27-0.47)	0.93 (0.91-0.96)	0.55 (0.42-0.68)	0.87 (0.83-0.9)	0.88	0.83
Nepal	CAD4TB	92	0.36 (0.27-0.47)	0.94 (0.91-0.96)	0.57 (0.43-0.69)	0.87 (0.83-0.9)	0.88	0.83
Nepal	CAD4TB	93	0.35 (0.26-0.46)	0.95 (0.92-0.97)	0.6 (0.46-0.73)	0.87 (0.83-0.9)	0.89	0.84
Nepal	CAD4TB	94	0.35 (0.26-0.46)	0.96 (0.93-0.97)	0.65 (0.5-0.78)	0.87 (0.83-0.9)	0.9	0.85
Nepal	CAD4TB	95	0.32 (0.23-0.42)	0.96 (0.94-0.98)	0.65 (0.5-0.79)	0.86 (0.83-0.89)	0.91	0.84
Nepal	CAD4TB	96	0.31 (0.22-0.41)	0.97 (0.94-0.98)	0.67 (0.51-0.81)	0.86 (0.83-0.89)	0.92	0.85
Nepal	CAD4TB	97	0.27 (0.18-0.37)	0.97 (0.95-0.99)	0.68 (0.5-0.82)	0.86 (0.82-0.89)	0.93	0.84
Nepal	CAD4TB	98	0.27 (0.18-0.37)	0.97 (0.95-0.99)	0.69 (0.52-0.84)	0.86 (0.82-0.89)	0.93	0.84
Nepal	CAD4TB	99	0.27 (0.18-0.37)	0.97 (0.95-0.99)	0.69 (0.52-0.84)	0.86 (0.82-0.89)	0.93	0.84
Nepal	Lunit	0	1 (0.96-1)	0 (0-0.01)	0.18 (0.15-0.22)	NA	0	0.18
Nepal	Lunit	0.01	1 (0.96-1)	0.11 (0.09-0.15)	0.2 (0.17-0.24)	1 (0.93-1)	0.09	0.18
Nepal	Lunit	0.02	1 (0.96-1)	0.21 (0.17-0.25)	0.22 (0.18-0.26)	1 (0.96-1)	0.17	0.18
Nepal	Lunit	0.03	1 (0.96-1)	0.24 (0.2-0.28)	0.23 (0.19-0.27)	1 (0.96-1)	0.19	0.19
Nepal	Lunit	0.04	1 (0.96-1)	0.25 (0.21-0.29)	0.23 (0.19-0.27)	1 (0.97-1)	0.2	0.21
Nepal	Lunit	0.05	1 (0.96-1)	0.25 (0.21-0.3)	0.23 (0.19-0.27)	1 (0.97-1)	0.21	0.23
Nepal	Lunit	0.06	1 (0.96-1)	0.27 (0.22-0.31)	0.23 (0.19-0.28)	1 (0.97-1)	0.22	0.28
Nepal	Lunit	0.07	1 (0.96-1)	0.28 (0.24-0.33)	0.24 (0.2-0.28)	1 (0.97-1)	0.23	0.3
Nepal	Lunit	0.08	1 (0.96-1)	0.3 (0.26-0.35)	0.24 (0.2-0.29)	1 (0.97-1)	0.24	0.34
Nepal	Lunit	0.09	1 (0.96-1)	0.31 (0.27-0.36)	0.25 (0.2-0.29)	1 (0.97-1)	0.26	0.36
Nepal	Lunit	0.1	1 (0.96-1)	0.33 (0.28-0.37)	0.25 (0.21-0.3)	1 (0.97-1)	0.27	0.37
Nepal	Lunit	0.11	1 (0.96-1)	0.34 (0.3-0.39)	0.25 (0.21-0.3)	1 (0.97-1)	0.28	0.39
Nepal	Lunit	0.12	1 (0.96-1)	0.35 (0.3-0.4)	0.26 (0.21-0.3)	1 (0.98-1)	0.29	0.41
Nepal	Lunit	0.13	1 (0.96-1)	0.37 (0.32-0.42)	0.26 (0.22-0.31)	1 (0.98-1)	0.3	0.43
Nepal	Lunit	0.14	0.99 (0.94-1)	0.38 (0.33-0.43)	0.26 (0.22-0.31)	0.99 (0.97-1)	0.31	0.45
Nepal	Lunit	0.15	0.99 (0.94-1)	0.38 (0.34-0.43)	0.26 (0.22-0.31)	0.99 (0.97-1)	0.32	0.47
Nepal	Lunit	0.16	0.99 (0.94-1)	0.39 (0.35-0.44)	0.27 (0.22-0.32)	0.99 (0.97-1)	0.32	0.48

Site	Deep Learning System	Abnormality Score	Sensitivity	Specificity	Positive Predictive Value	Negative Predictive Value	Xpert Saving	Accuracy
Nepal	Lunit	0.17	0.99 (0.94-1)	0.4 (0.35-0.45)	0.27 (0.22-0.32)	0.99 (0.97-1)	0.33	0.48
Nepal	Lunit	0.18	0.99 (0.94-1)	0.41 (0.37-0.46)	0.27 (0.23-0.32)	0.99 (0.97-1)	0.34	0.49
Nepal	Lunit	0.19	0.99 (0.94-1)	0.42 (0.38-0.47)	0.28 (0.23-0.33)	0.99 (0.97-1)	0.35	0.5
Nepal	Lunit	0.2	0.99 (0.94-1)	0.43 (0.38-0.47)	0.28 (0.23-0.33)	0.99 (0.97-1)	0.35	0.51
Nepal	Lunit	0.21	0.99 (0.94-1)	0.43 (0.39-0.48)	0.28 (0.23-0.33)	0.99 (0.97-1)	0.36	0.52
Nepal	Lunit	0.22	0.99 (0.94-1)	0.44 (0.39-0.49)	0.28 (0.24-0.34)	0.99 (0.97-1)	0.36	0.53
Nepal	Lunit	0.23	0.99 (0.94-1)	0.44 (0.4-0.49)	0.28 (0.24-0.34)	0.99 (0.97-1)	0.37	0.54
Nepal	Lunit	0.24	0.98 (0.93-1)	0.45 (0.4-0.5)	0.28 (0.24-0.34)	0.99 (0.96-1)	0.37	0.55
Nepal	Lunit	0.25	0.98 (0.93-1)	0.46 (0.41-0.5)	0.29 (0.24-0.34)	0.99 (0.96-1)	0.38	0.56
Nepal	Lunit	0.26	0.98 (0.93-1)	0.47 (0.42-0.51)	0.29 (0.24-0.34)	0.99 (0.96-1)	0.38	0.57
Nepal	Lunit	0.27	0.98 (0.93-1)	0.47 (0.42-0.52)	0.29 (0.24-0.35)	0.99 (0.96-1)	0.39	0.58
Nepal	Lunit	0.28	0.97 (0.91-0.99)	0.47 (0.42-0.52)	0.29 (0.24-0.34)	0.99 (0.96-1)	0.39	0.58
Nepal	Lunit	0.29	0.97 (0.91-0.99)	0.48 (0.43-0.52)	0.29 (0.24-0.35)	0.99 (0.96-1)	0.39	0.59
Nepal	Lunit	0.3	0.97 (0.91-0.99)	0.48 (0.43-0.53)	0.29 (0.24-0.35)	0.99 (0.96-1)	0.4	0.59
Nepal	Lunit	0.31	0.97 (0.91-0.99)	0.48 (0.43-0.53)	0.29 (0.24-0.35)	0.99 (0.96-1)	0.4	0.6
Nepal	Lunit	0.32	0.97 (0.91-0.99)	0.48 (0.43-0.53)	0.29 (0.24-0.35)	0.99 (0.96-1)	0.4	0.6
Nepal	Lunit	0.33	0.97 (0.91-0.99)	0.48 (0.43-0.53)	0.29 (0.24-0.35)	0.99 (0.96-1)	0.4	0.61
Nepal	Lunit	0.34	0.97 (0.91-0.99)	0.48 (0.43-0.53)	0.29 (0.24-0.35)	0.99 (0.96-1)	0.4	0.62
Nepal	Lunit	0.35	0.96 (0.89-0.99)	0.49 (0.44-0.54)	0.3 (0.24-0.35)	0.98 (0.95-0.99)	0.41	0.63
Nepal	Lunit	0.36	0.96 (0.89-0.99)	0.49 (0.44-0.54)	0.3 (0.25-0.35)	0.98 (0.95-0.99)	0.41	0.64
Nepal	Lunit	0.37	0.96 (0.89-0.99)	0.5 (0.45-0.55)	0.3 (0.25-0.35)	0.98 (0.95-0.99)	0.42	0.64
Nepal	Lunit	0.38	0.96 (0.89-0.99)	0.5 (0.45-0.55)	0.3 (0.25-0.36)	0.98 (0.95-0.99)	0.42	0.65
Nepal	Lunit	0.39	0.96 (0.89-0.99)	0.5 (0.45-0.55)	0.3 (0.25-0.36)	0.98 (0.95-0.99)	0.42	0.65
Nepal	Lunit	0.4	0.96 (0.89-0.99)	0.5 (0.45-0.55)	0.3 (0.25-0.36)	0.98 (0.95-0.99)	0.42	0.66
Nepal	Lunit	0.41	0.96 (0.89-0.99)	0.51 (0.46-0.55)	0.3 (0.25-0.36)	0.98 (0.95-0.99)	0.42	0.67
Nepal	Lunit	0.42	0.96 (0.89-0.99)	0.51 (0.46-0.56)	0.3 (0.25-0.36)	0.98 (0.95-1)	0.43	0.66
Nepal	Lunit	0.43	0.96 (0.89-0.99)	0.51 (0.46-0.56)	0.3 (0.25-0.36)	0.98 (0.95-1)	0.43	0.67
Nepal	Lunit	0.44	0.96 (0.89-0.99)	0.51 (0.46-0.56)	0.3 (0.25-0.36)	0.98 (0.95-1)	0.43	0.68
Nepal	Lunit	0.45	0.96 (0.89-0.99)	0.52 (0.47-0.57)	0.31 (0.26-0.37)	0.98 (0.95-1)	0.43	0.68
Nepal	Lunit	0.46	0.96 (0.89-0.99)	0.52 (0.48-0.57)	0.31 (0.26-0.37)	0.98 (0.96-1)	0.44	0.7
Nepal	Lunit	0.47	0.96 (0.89-0.99)	0.53 (0.48-0.58)	0.31 (0.26-0.37)	0.98 (0.96-1)	0.44	0.7
Nepal	Lunit	0.48	0.96 (0.89-0.99)	0.53 (0.48-0.58)	0.31 (0.26-0.37)	0.98 (0.96-1)	0.44	0.7

Site	Deep Learning System	Abnormality Score	Sensitivity	Specificity	Positive Predictive Value	Negative Predictive Value	Xpert Saving	Accuracy
Nepal	Lunit	0.49	0.96 (0.89-0.99)	0.53 (0.49-0.58)	0.31 (0.26-0.37)	0.98 (0.96-1)	0.44	0.7
Nepal	Lunit	0.5	0.96 (0.89-0.99)	0.54 (0.49-0.59)	0.32 (0.26-0.38)	0.98 (0.96-1)	0.45	0.71
Nepal	Lunit	0.51	0.96 (0.89-0.99)	0.54 (0.49-0.59)	0.32 (0.26-0.38)	0.98 (0.96-1)	0.45	0.72
Nepal	Lunit	0.52	0.96 (0.89-0.99)	0.55 (0.5-0.6)	0.32 (0.27-0.38)	0.98 (0.96-1)	0.46	0.72
Nepal	Lunit	0.53	0.96 (0.89-0.99)	0.55 (0.5-0.6)	0.32 (0.27-0.38)	0.98 (0.96-1)	0.46	0.72
Nepal	Lunit	0.54	0.96 (0.89-0.99)	0.55 (0.5-0.6)	0.32 (0.27-0.38)	0.98 (0.96-1)	0.46	0.73
Nepal	Lunit	0.55	0.96 (0.89-0.99)	0.56 (0.51-0.6)	0.32 (0.27-0.38)	0.98 (0.96-1)	0.46	0.73
Nepal	Lunit	0.56	0.96 (0.89-0.99)	0.56 (0.51-0.61)	0.33 (0.27-0.39)	0.98 (0.96-1)	0.47	0.73
Nepal	Lunit	0.57	0.96 (0.89-0.99)	0.57 (0.52-0.61)	0.33 (0.27-0.39)	0.98 (0.96-1)	0.47	0.74
Nepal	Lunit	0.58	0.96 (0.89-0.99)	0.57 (0.52-0.62)	0.33 (0.28-0.39)	0.98 (0.96-1)	0.47	0.75
Nepal	Lunit	0.59	0.96 (0.89-0.99)	0.57 (0.52-0.62)	0.33 (0.28-0.39)	0.98 (0.96-1)	0.47	0.75
Nepal	Lunit	0.6	0.96 (0.89-0.99)	0.57 (0.53-0.62)	0.33 (0.28-0.39)	0.98 (0.96-1)	0.48	0.76
Nepal	Lunit	0.61	0.96 (0.89-0.99)	0.58 (0.53-0.62)	0.34 (0.28-0.4)	0.98 (0.96-1)	0.48	0.76
Nepal	Lunit	0.62	0.96 (0.89-0.99)	0.58 (0.53-0.63)	0.34 (0.28-0.4)	0.98 (0.96-1)	0.48	0.76
Nepal	Lunit	0.63	0.96 (0.89-0.99)	0.59 (0.54-0.63)	0.34 (0.28-0.4)	0.98 (0.96-1)	0.49	0.77
Nepal	Lunit	0.64	0.96 (0.89-0.99)	0.59 (0.54-0.63)	0.34 (0.28-0.4)	0.98 (0.96-1)	0.49	0.78
Nepal	Lunit	0.65	0.96 (0.89-0.99)	0.59 (0.54-0.63)	0.34 (0.28-0.4)	0.98 (0.96-1)	0.49	0.8
Nepal	Lunit	0.66	0.96 (0.89-0.99)	0.59 (0.54-0.64)	0.34 (0.29-0.4)	0.98 (0.96-1)	0.49	0.8
Nepal	Lunit	0.67	0.96 (0.89-0.99)	0.6 (0.55-0.64)	0.35 (0.29-0.41)	0.98 (0.96-1)	0.5	0.8
Nepal	Lunit	0.68	0.96 (0.89-0.99)	0.6 (0.55-0.65)	0.35 (0.29-0.41)	0.98 (0.96-1)	0.5	0.81
Nepal	Lunit	0.69	0.96 (0.89-0.99)	0.6 (0.55-0.65)	0.35 (0.29-0.41)	0.98 (0.96-1)	0.5	0.81
Nepal	Lunit	0.7	0.95 (0.88-0.98)	0.61 (0.56-0.66)	0.35 (0.29-0.41)	0.98 (0.96-0.99)	0.51	0.81
Nepal	Lunit	0.71	0.95 (0.88-0.98)	0.62 (0.57-0.66)	0.36 (0.3-0.42)	0.98 (0.96-0.99)	0.51	0.82
Nepal	Lunit	0.72	0.95 (0.88-0.98)	0.63 (0.58-0.67)	0.36 (0.3-0.43)	0.98 (0.96-0.99)	0.52	0.82
Nepal	Lunit	0.73	0.95 (0.88-0.98)	0.63 (0.58-0.68)	0.36 (0.3-0.43)	0.98 (0.96-0.99)	0.52	0.82
Nepal	Lunit	0.74	0.95 (0.88-0.98)	0.63 (0.59-0.68)	0.37 (0.31-0.43)	0.98 (0.96-0.99)	0.53	0.83
Nepal	Lunit	0.75	0.95 (0.88-0.98)	0.63 (0.59-0.68)	0.37 (0.31-0.43)	0.98 (0.96-0.99)	0.53	0.84
Nepal	Lunit	0.76	0.95 (0.88-0.98)	0.64 (0.59-0.68)	0.37 (0.31-0.43)	0.98 (0.96-0.99)	0.53	0.84
Nepal	Lunit	0.77	0.95 (0.88-0.98)	0.65 (0.6-0.69)	0.37 (0.31-0.44)	0.98 (0.96-0.99)	0.54	0.84
Nepal	Lunit	0.78	0.95 (0.88-0.98)	0.65 (0.61-0.7)	0.38 (0.32-0.44)	0.98 (0.96-0.99)	0.54	0.85
Nepal	Lunit	0.79	0.95 (0.88-0.98)	0.66 (0.61-0.7)	0.38 (0.32-0.45)	0.98 (0.96-0.99)	0.55	0.84
Nepal	Lunit	0.8	0.95 (0.88-0.98)	0.66 (0.61-0.7)	0.38 (0.32-0.45)	0.98 (0.96-0.99)	0.55	0.85

Site	Deep Learning System	Abnormality Score	Sensitivity	Specificity	Positive Predictive Value	Negative Predictive Value	Xpert Saving	Accuracy
Nepal	Lunit	0.81	0.94 (0.87-0.98)	0.67 (0.62-0.71)	0.39 (0.32-0.45)	0.98 (0.96-0.99)	0.56	0.86
Nepal	Lunit	0.82	0.94 (0.87-0.98)	0.67 (0.62-0.71)	0.39 (0.32-0.45)	0.98 (0.96-0.99)	0.56	0.87
Nepal	Lunit	0.83	0.94 (0.87-0.98)	0.68 (0.63-0.72)	0.39 (0.33-0.46)	0.98 (0.96-0.99)	0.57	0.87
Nepal	Lunit	0.84	0.94 (0.87-0.98)	0.69 (0.64-0.73)	0.4 (0.34-0.47)	0.98 (0.96-0.99)	0.57	0.87
Nepal	Lunit	0.85	0.94 (0.87-0.98)	0.7 (0.65-0.74)	0.41 (0.34-0.48)	0.98 (0.96-0.99)	0.58	0.87
Nepal	Lunit	0.86	0.93 (0.85-0.97)	0.7 (0.66-0.75)	0.41 (0.34-0.48)	0.98 (0.95-0.99)	0.59	0.87
Nepal	Lunit	0.87	0.91 (0.84-0.96)	0.71 (0.66-0.75)	0.41 (0.35-0.48)	0.97 (0.95-0.99)	0.6	0.88
Nepal	Lunit	0.88	0.91 (0.84-0.96)	0.73 (0.68-0.77)	0.43 (0.36-0.5)	0.97 (0.95-0.99)	0.61	0.88
Nepal	Lunit	0.89	0.91 (0.84-0.96)	0.73 (0.69-0.77)	0.43 (0.36-0.5)	0.97 (0.95-0.99)	0.61	0.88
Nepal	Lunit	0.9	0.91 (0.84-0.96)	0.75 (0.7-0.79)	0.45 (0.37-0.52)	0.98 (0.95-0.99)	0.63	0.88
Nepal	Lunit	0.91	0.89 (0.81-0.95)	0.76 (0.71-0.8)	0.45 (0.38-0.52)	0.97 (0.94-0.99)	0.64	0.87
Nepal	Lunit	0.92	0.89 (0.81-0.95)	0.77 (0.73-0.81)	0.47 (0.39-0.55)	0.97 (0.95-0.99)	0.65	0.87
Nepal	Lunit	0.93	0.87 (0.79-0.93)	0.78 (0.73-0.82)	0.47 (0.39-0.54)	0.96 (0.94-0.98)	0.66	0.85
Nepal	Lunit	0.94	0.84 (0.75-0.91)	0.81 (0.77-0.84)	0.49 (0.41-0.57)	0.96 (0.93-0.98)	0.69	0.83
Nepal	Lunit	0.95	0.84 (0.75-0.91)	0.84 (0.8-0.87)	0.54 (0.46-0.62)	0.96 (0.93-0.98)	0.72	0.83
Nepal	Lunit	0.96	0.81 (0.71-0.88)	0.86 (0.83-0.9)	0.57 (0.48-0.66)	0.95 (0.93-0.97)	0.74	0.82
Nepal	Lunit	0.97	0.73 (0.63-0.82)	0.9 (0.87-0.93)	0.62 (0.52-0.71)	0.94 (0.91-0.96)	0.78	0.82
Nepal	Lunit	0.98	0.63 (0.52-0.73)	0.94 (0.92-0.96)	0.71 (0.6-0.81)	0.92 (0.89-0.94)	0.84	0.82
Nepal	Lunit	0.99	0.34 (0.25-0.45)	0.98 (0.96-0.99)	0.76 (0.61-0.88)	0.87 (0.84-0.9)	0.92	0.82
Nepal	Lunit	1	0 (0-0.04)	1 (0.99-1)	NA	0.82 (0.78-0.85)	1	0.18
Nepal	qXR	0.01	1 (0.96-1)	0 (0-0.01)	0.18 (0.15-0.22)	NA	0	0.28
Nepal	qXR	0.02	1 (0.96-1)	0 (0-0.01)	0.18 (0.15-0.22)	NA	0	0.35
Nepal	qXR	0.03	1 (0.96-1)	0 (0-0.01)	0.18 (0.15-0.22)	NA	0	0.38
Nepal	qXR	0.04	1 (0.96-1)	0 (0-0.02)	0.18 (0.15-0.22)	1 (0.16-1)	0	0.39
Nepal	qXR	0.05	1 (0.96-1)	0.03 (0.01-0.05)	0.19 (0.15-0.22)	1 (0.74-1)	0.02	0.39
Nepal	qXR	0.06	1 (0.96-1)	0.05 (0.03-0.08)	0.19 (0.16-0.23)	1 (0.85-1)	0.04	0.4
Nepal	qXR	0.07	1 (0.96-1)	0.11 (0.09-0.15)	0.2 (0.17-0.24)	1 (0.93-1)	0.09	0.41
Nepal	qXR	0.08	1 (0.96-1)	0.15 (0.11-0.18)	0.21 (0.17-0.25)	1 (0.94-1)	0.12	0.43
Nepal	qXR	0.09	1 (0.96-1)	0.19 (0.15-0.23)	0.22 (0.18-0.26)	1 (0.95-1)	0.15	0.44
Nepal	qXR	0.1	1 (0.96-1)	0.22 (0.18-0.26)	0.22 (0.18-0.26)	1 (0.96-1)	0.18	0.45
Nepal	qXR	0.11	1 (0.96-1)	0.24 (0.2-0.28)	0.23 (0.19-0.27)	1 (0.96-1)	0.19	0.46
Nepal	qXR	0.12	1 (0.96-1)	0.26 (0.22-0.3)	0.23 (0.19-0.28)	1 (0.97-1)	0.21	0.47

Site	Deep Learning System	Abnormality Score	Sensitivity	Specificity	Positive Predictive Value	Negative Predictive Value	Xpert Saving	Accuracy
Nepal	qXR	0.13	0.99 (0.94-1)	0.28 (0.24-0.33)	0.23 (0.19-0.28)	0.99 (0.95-1)	0.23	0.48
Nepal	qXR	0.14	0.99 (0.94-1)	0.31 (0.27-0.36)	0.24 (0.2-0.29)	0.99 (0.96-1)	0.26	0.49
Nepal	qXR	0.15	0.99 (0.94-1)	0.33 (0.28-0.37)	0.25 (0.2-0.29)	0.99 (0.96-1)	0.27	0.5
Nepal	qXR	0.16	0.98 (0.93-1)	0.35 (0.31-0.4)	0.25 (0.21-0.3)	0.99 (0.95-1)	0.29	0.5
Nepal	qXR	0.17	0.98 (0.93-1)	0.36 (0.32-0.41)	0.26 (0.21-0.3)	0.99 (0.95-1)	0.3	0.51
Nepal	qXR	0.18	0.98 (0.93-1)	0.37 (0.32-0.42)	0.26 (0.21-0.31)	0.99 (0.96-1)	0.31	0.52
Nepal	qXR	0.19	0.98 (0.93-1)	0.38 (0.34-0.43)	0.26 (0.22-0.31)	0.99 (0.96-1)	0.32	0.53
Nepal	qXR	0.2	0.98 (0.93-1)	0.4 (0.35-0.45)	0.27 (0.22-0.32)	0.99 (0.96-1)	0.33	0.53
Nepal	qXR	0.21	0.98 (0.93-1)	0.4 (0.36-0.45)	0.27 (0.22-0.32)	0.99 (0.96-1)	0.33	0.54
Nepal	qXR	0.22	0.98 (0.93-1)	0.42 (0.37-0.47)	0.27 (0.23-0.32)	0.99 (0.96-1)	0.35	0.54
Nepal	qXR	0.23	0.98 (0.93-1)	0.43 (0.38-0.48)	0.28 (0.23-0.33)	0.99 (0.96-1)	0.36	0.54
Nepal	qXR	0.24	0.98 (0.93-1)	0.44 (0.39-0.49)	0.28 (0.23-0.33)	0.99 (0.96-1)	0.37	0.55
Nepal	qXR	0.25	0.98 (0.93-1)	0.46 (0.41-0.5)	0.29 (0.24-0.34)	0.99 (0.96-1)	0.38	0.55
Nepal	qXR	0.26	0.98 (0.93-1)	0.47 (0.42-0.51)	0.29 (0.24-0.34)	0.99 (0.96-1)	0.38	0.56
Nepal	qXR	0.27	0.98 (0.93-1)	0.48 (0.43-0.52)	0.29 (0.24-0.35)	0.99 (0.96-1)	0.39	0.56
Nepal	qXR	0.28	0.98 (0.93-1)	0.49 (0.44-0.54)	0.3 (0.25-0.35)	0.99 (0.97-1)	0.4	0.56
Nepal	qXR	0.29	0.98 (0.93-1)	0.49 (0.45-0.54)	0.3 (0.25-0.36)	0.99 (0.97-1)	0.41	0.57
Nepal	qXR	0.3	0.98 (0.93-1)	0.51 (0.46-0.55)	0.31 (0.25-0.36)	0.99 (0.97-1)	0.42	0.57
Nepal	qXR	0.31	0.98 (0.93-1)	0.51 (0.46-0.56)	0.31 (0.26-0.36)	0.99 (0.97-1)	0.42	0.57
Nepal	qXR	0.32	0.98 (0.93-1)	0.51 (0.46-0.56)	0.31 (0.26-0.37)	0.99 (0.97-1)	0.42	0.57
Nepal	qXR	0.33	0.98 (0.93-1)	0.52 (0.47-0.57)	0.31 (0.26-0.37)	0.99 (0.97-1)	0.43	0.57
Nepal	qXR	0.34	0.98 (0.93-1)	0.53 (0.48-0.58)	0.32 (0.27-0.38)	0.99 (0.97-1)	0.44	0.57
Nepal	qXR	0.35	0.98 (0.93-1)	0.54 (0.49-0.59)	0.32 (0.27-0.38)	0.99 (0.97-1)	0.45	0.57
Nepal	qXR	0.36	0.98 (0.93-1)	0.55 (0.5-0.6)	0.33 (0.27-0.38)	0.99 (0.97-1)	0.45	0.58
Nepal	qXR	0.37	0.98 (0.93-1)	0.56 (0.51-0.61)	0.33 (0.28-0.39)	0.99 (0.97-1)	0.46	0.58
Nepal	qXR	0.38	0.98 (0.93-1)	0.57 (0.52-0.62)	0.34 (0.28-0.4)	0.99 (0.97-1)	0.47	0.58
Nepal	qXR	0.39	0.98 (0.93-1)	0.58 (0.53-0.62)	0.34 (0.28-0.4)	0.99 (0.97-1)	0.48	0.59
Nepal	qXR	0.4	0.98 (0.93-1)	0.58 (0.53-0.63)	0.34 (0.29-0.4)	0.99 (0.97-1)	0.48	0.59
Nepal	qXR	0.41	0.98 (0.93-1)	0.59 (0.54-0.64)	0.35 (0.29-0.41)	0.99 (0.97-1)	0.49	0.59
Nepal	qXR	0.42	0.98 (0.93-1)	0.6 (0.55-0.64)	0.35 (0.29-0.41)	0.99 (0.97-1)	0.49	0.59
Nepal	qXR	0.43	0.97 (0.91-0.99)	0.6 (0.55-0.64)	0.35 (0.29-0.41)	0.99 (0.97-1)	0.49	0.59
Nepal	qXR	0.44	0.97 (0.91-0.99)	0.61 (0.56-0.65)	0.35 (0.3-0.42)	0.99 (0.97-1)	0.5	0.59

Site	Deep Learning System	Abnormality Score	Sensitivity	Specificity	Positive Predictive Value	Negative Predictive Value	Xpert Saving	Accuracy
Nepal	qXR	0.45	0.97 (0.91-0.99)	0.61 (0.56-0.66)	0.36 (0.3-0.42)	0.99 (0.97-1)	0.51	0.6
Nepal	qXR	0.46	0.97 (0.91-0.99)	0.62 (0.57-0.67)	0.36 (0.3-0.43)	0.99 (0.97-1)	0.51	0.6
Nepal	qXR	0.47	0.97 (0.91-0.99)	0.63 (0.59-0.68)	0.37 (0.31-0.44)	0.99 (0.97-1)	0.52	0.61
Nepal	qXR	0.48	0.97 (0.91-0.99)	0.64 (0.6-0.69)	0.38 (0.32-0.44)	0.99 (0.97-1)	0.53	0.61
Nepal	qXR	0.49	0.97 (0.91-0.99)	0.65 (0.6-0.69)	0.38 (0.32-0.44)	0.99 (0.97-1)	0.53	0.61
Nepal	qXR	0.5	0.95 (0.88-0.98)	0.65 (0.6-0.7)	0.38 (0.32-0.44)	0.98 (0.96-0.99)	0.54	0.62
Nepal	qXR	0.51	0.95 (0.88-0.98)	0.66 (0.61-0.71)	0.38 (0.32-0.45)	0.98 (0.96-0.99)	0.55	0.62
Nepal	qXR	0.52	0.95 (0.88-0.98)	0.67 (0.62-0.71)	0.39 (0.32-0.45)	0.98 (0.96-0.99)	0.55	0.63
Nepal	qXR	0.53	0.94 (0.87-0.98)	0.67 (0.62-0.71)	0.39 (0.32-0.45)	0.98 (0.96-0.99)	0.56	0.63
Nepal	qXR	0.54	0.94 (0.87-0.98)	0.67 (0.63-0.72)	0.39 (0.33-0.46)	0.98 (0.96-0.99)	0.56	0.63
Nepal	qXR	0.55	0.94 (0.87-0.98)	0.68 (0.64-0.73)	0.4 (0.33-0.47)	0.98 (0.96-0.99)	0.57	0.63
Nepal	qXR	0.56	0.94 (0.87-0.98)	0.69 (0.64-0.73)	0.4 (0.33-0.47)	0.98 (0.96-0.99)	0.57	0.63
Nepal	qXR	0.57	0.94 (0.87-0.98)	0.69 (0.64-0.73)	0.4 (0.34-0.47)	0.98 (0.96-0.99)	0.57	0.64
Nepal	qXR	0.58	0.94 (0.87-0.98)	0.69 (0.65-0.74)	0.41 (0.34-0.47)	0.98 (0.96-0.99)	0.58	0.64
Nepal	qXR	0.59	0.94 (0.87-0.98)	0.71 (0.66-0.75)	0.42 (0.35-0.49)	0.98 (0.96-0.99)	0.59	0.64
Nepal	qXR	0.6	0.94 (0.87-0.98)	0.71 (0.67-0.76)	0.42 (0.35-0.49)	0.98 (0.96-0.99)	0.59	0.64
Nepal	qXR	0.61	0.94 (0.87-0.98)	0.71 (0.67-0.76)	0.42 (0.36-0.49)	0.98 (0.96-0.99)	0.6	0.65
Nepal	qXR	0.62	0.94 (0.87-0.98)	0.72 (0.67-0.76)	0.43 (0.36-0.5)	0.98 (0.96-0.99)	0.6	0.65
Nepal	qXR	0.63	0.94 (0.87-0.98)	0.72 (0.68-0.77)	0.43 (0.36-0.5)	0.98 (0.96-0.99)	0.6	0.65
Nepal	qXR	0.64	0.93 (0.85-0.97)	0.74 (0.69-0.78)	0.44 (0.37-0.51)	0.98 (0.96-0.99)	0.62	0.65
Nepal	qXR	0.65	0.93 (0.85-0.97)	0.75 (0.71-0.79)	0.45 (0.38-0.53)	0.98 (0.96-0.99)	0.63	0.65
Nepal	qXR	0.66	0.93 (0.85-0.97)	0.77 (0.73-0.81)	0.48 (0.4-0.55)	0.98 (0.96-0.99)	0.64	0.66
Nepal	qXR	0.67	0.91 (0.84-0.96)	0.77 (0.73-0.81)	0.48 (0.4-0.55)	0.98 (0.95-0.99)	0.65	0.66
Nepal	qXR	0.68	0.91 (0.84-0.96)	0.78 (0.73-0.82)	0.48 (0.4-0.55)	0.98 (0.95-0.99)	0.65	0.66
Nepal	qXR	0.69	0.91 (0.84-0.96)	0.78 (0.74-0.82)	0.49 (0.41-0.56)	0.98 (0.95-0.99)	0.66	0.67
Nepal	qXR	0.7	0.9 (0.83-0.96)	0.79 (0.75-0.83)	0.49 (0.42-0.57)	0.97 (0.95-0.99)	0.67	0.67
Nepal	qXR	0.71	0.89 (0.81-0.95)	0.8 (0.75-0.83)	0.49 (0.42-0.57)	0.97 (0.95-0.99)	0.67	0.68
Nepal	qXR	0.72	0.87 (0.79-0.93)	0.81 (0.76-0.84)	0.5 (0.42-0.58)	0.97 (0.94-0.98)	0.68	0.69
Nepal	qXR	0.73	0.86 (0.78-0.92)	0.81 (0.77-0.84)	0.5 (0.42-0.58)	0.96 (0.94-0.98)	0.69	0.69
Nepal	qXR	0.74	0.86 (0.78-0.92)	0.81 (0.77-0.85)	0.51 (0.43-0.59)	0.96 (0.94-0.98)	0.69	0.69
Nepal	qXR	0.75	0.86 (0.78-0.92)	0.82 (0.78-0.86)	0.52 (0.44-0.6)	0.96 (0.94-0.98)	0.7	0.69
Nepal	qXR	0.76	0.86 (0.78-0.92)	0.83 (0.79-0.87)	0.54 (0.45-0.62)	0.96 (0.94-0.98)	0.71	0.7

Site	Deep Learning System	Abnormality Score	Sensitivity	Specificity	Positive Predictive Value	Negative Predictive Value	Xpert Saving	Accuracy
Nepal	qXR	0.77	0.84 (0.75-0.91)	0.84 (0.8-0.87)	0.54 (0.46-0.62)	0.96 (0.93-0.98)	0.72	0.7
Nepal	qXR	0.78	0.82 (0.73-0.89)	0.85 (0.81-0.88)	0.55 (0.46-0.63)	0.95 (0.93-0.97)	0.73	0.71
Nepal	qXR	0.79	0.82 (0.73-0.89)	0.85 (0.82-0.89)	0.55 (0.47-0.64)	0.95 (0.93-0.97)	0.73	0.71
Nepal	qXR	0.8	0.8 (0.7-0.87)	0.85 (0.82-0.89)	0.55 (0.46-0.63)	0.95 (0.92-0.97)	0.73	0.71
Nepal	qXR	0.81	0.8 (0.7-0.87)	0.86 (0.83-0.9)	0.57 (0.48-0.65)	0.95 (0.92-0.97)	0.74	0.72
Nepal	qXR	0.82	0.79 (0.69-0.86)	0.88 (0.84-0.91)	0.59 (0.5-0.67)	0.95 (0.92-0.97)	0.76	0.72
Nepal	qXR	0.83	0.78 (0.68-0.86)	0.89 (0.85-0.91)	0.6 (0.51-0.69)	0.95 (0.92-0.97)	0.77	0.72
Nepal	qXR	0.84	0.76 (0.66-0.84)	0.9 (0.86-0.93)	0.62 (0.53-0.71)	0.94 (0.92-0.96)	0.78	0.73
Nepal	qXR	0.85	0.72 (0.62-0.81)	0.9 (0.87-0.93)	0.63 (0.53-0.72)	0.94 (0.91-0.96)	0.79	0.74
Nepal	qXR	0.86	0.7 (0.6-0.79)	0.91 (0.88-0.94)	0.64 (0.54-0.73)	0.93 (0.9-0.95)	0.8	0.74
Nepal	qXR	0.87	0.67 (0.57-0.76)	0.92 (0.89-0.94)	0.65 (0.55-0.74)	0.93 (0.9-0.95)	0.81	0.75
Nepal	qXR	0.88	0.67 (0.57-0.76)	0.92 (0.89-0.95)	0.66 (0.55-0.75)	0.93 (0.9-0.95)	0.81	0.76
Nepal	qXR	0.89	0.64 (0.53-0.73)	0.93 (0.9-0.95)	0.67 (0.57-0.77)	0.92 (0.89-0.94)	0.83	0.77
Nepal	qXR	0.9	0.6 (0.49-0.7)	0.95 (0.92-0.97)	0.71 (0.6-0.81)	0.91 (0.88-0.94)	0.85	0.78
Nepal	qXR	0.91	0.53 (0.43-0.64)	0.96 (0.94-0.98)	0.75 (0.63-0.84)	0.9 (0.87-0.93)	0.87	0.78
Nepal	qXR	0.92	0.47 (0.36-0.57)	0.96 (0.94-0.98)	0.72 (0.59-0.83)	0.89 (0.86-0.92)	0.88	0.8
Nepal	qXR	0.93	0.38 (0.28-0.49)	0.97 (0.95-0.99)	0.77 (0.62-0.88)	0.88 (0.84-0.9)	0.91	0.79
Nepal	qXR	0.94	0.27 (0.18-0.37)	0.98 (0.97-0.99)	0.78 (0.6-0.91)	0.86 (0.82-0.89)	0.94	0.81
Nepal	qXR	0.95	0.15 (0.08-0.24)	0.99 (0.97-0.99)	0.7 (0.46-0.88)	0.84 (0.8-0.87)	0.96	0.84
Nepal	qXR	0.96	0.1 (0.04-0.17)	1 (0.99-1)	0.9 (0.55-1)	0.83 (0.8-0.86)	0.98	0.85
Nepal	qXR	0.97	0.03 (0.01-0.09)	1 (0.99-1)	0.75 (0.19-0.99)	0.82 (0.79-0.85)	0.99	0.87
Nepal	qXR	0.98	0 (0-0.04)	1 (0.99-1)	NA	0.82 (0.78-0.85)	1	0.89
Nepal	qXR	0.99	0 (0-0.04)	1 (0.99-1)	NA	0.82 (0.78-0.85)	1	0.86
Nepal	qXR	1	0 (0-0.04)	1 (0.99-1)	NA	0.82 (0.78-0.85)	1	0.82
Cameroon	CAD4TB	1	1 (0.78-1)	0 (0-0.01)	0.02 (0.01-0.04)	NA	0	0.02
Cameroon	CAD4TB	2	1 (0.78-1)	0 (0-0.01)	0.02 (0.01-0.04)	1 (0.03-1)	0	0.02
Cameroon	CAD4TB	3	1 (0.78-1)	0 (0-0.01)	0.02 (0.01-0.04)	1 (0.16-1)	0	0.02
Cameroon	CAD4TB	4	1 (0.78-1)	0.01 (0-0.02)	0.02 (0.01-0.04)	1 (0.59-1)	0.01	0.03
Cameroon	CAD4TB	5	1 (0.78-1)	0.02 (0.01-0.03)	0.02 (0.01-0.04)	1 (0.75-1)	0.02	0.04
Cameroon	CAD4TB	6	1 (0.78-1)	0.03 (0.01-0.04)	0.02 (0.01-0.04)	1 (0.8-1)	0.02	0.05
Cameroon	CAD4TB	7	1 (0.78-1)	0.03 (0.02-0.05)	0.02 (0.01-0.04)	1 (0.85-1)	0.03	0.06
Cameroon	CAD4TB	8	1 (0.78-1)	0.05 (0.04-0.07)	0.02 (0.01-0.04)	1 (0.9-1)	0.05	0.07

Site	Deep Learning System	Abnormality Score	Sensitivity	Specificity	Positive Predictive Value	Negative Predictive Value	Xpert Saving	Accuracy
Cameroon	CAD4TB	9	1 (0.78-1)	0.07 (0.05-0.09)	0.02 (0.01-0.04)	1 (0.92-1)	0.06	0.09
Cameroon	CAD4TB	10	1 (0.78-1)	0.08 (0.06-0.1)	0.02 (0.01-0.04)	1 (0.93-1)	0.08	0.1
Cameroon	CAD4TB	11	1 (0.78-1)	0.09 (0.07-0.11)	0.02 (0.01-0.04)	1 (0.94-1)	0.09	0.11
Cameroon	CAD4TB	12	1 (0.78-1)	0.1 (0.08-0.13)	0.02 (0.01-0.04)	1 (0.95-1)	0.1	0.12
Cameroon	CAD4TB	13	1 (0.78-1)	0.11 (0.09-0.14)	0.02 (0.01-0.04)	1 (0.95-1)	0.11	0.13
Cameroon	CAD4TB	14	1 (0.78-1)	0.12 (0.1-0.15)	0.02 (0.01-0.04)	1 (0.95-1)	0.12	0.14
Cameroon	CAD4TB	15	1 (0.78-1)	0.14 (0.11-0.16)	0.03 (0.01-0.04)	1 (0.96-1)	0.13	0.15
Cameroon	CAD4TB	16	1 (0.78-1)	0.15 (0.12-0.17)	0.03 (0.01-0.04)	1 (0.96-1)	0.14	0.16
Cameroon	CAD4TB	17	1 (0.78-1)	0.16 (0.13-0.19)	0.03 (0.01-0.04)	1 (0.97-1)	0.15	0.17
Cameroon	CAD4TB	18	1 (0.78-1)	0.18 (0.15-0.21)	0.03 (0.01-0.04)	1 (0.97-1)	0.17	0.19
Cameroon	CAD4TB	19	0.93 (0.68-1)	0.19 (0.16-0.22)	0.03 (0.01-0.04)	0.99 (0.96-1)	0.18	0.2
Cameroon	CAD4TB	20	0.93 (0.68-1)	0.2 (0.17-0.23)	0.03 (0.01-0.04)	0.99 (0.96-1)	0.19	0.21
Cameroon	CAD4TB	21	0.93 (0.68-1)	0.21 (0.18-0.25)	0.03 (0.01-0.04)	0.99 (0.96-1)	0.21	0.23
Cameroon	CAD4TB	22	0.93 (0.68-1)	0.21 (0.18-0.25)	0.03 (0.01-0.04)	0.99 (0.96-1)	0.21	0.23
Cameroon	CAD4TB	23	0.93 (0.68-1)	0.23 (0.2-0.26)	0.03 (0.01-0.04)	0.99 (0.96-1)	0.23	0.25
Cameroon	CAD4TB	24	0.93 (0.68-1)	0.24 (0.21-0.28)	0.03 (0.01-0.04)	0.99 (0.97-1)	0.24	0.26
Cameroon	CAD4TB	25	0.93 (0.68-1)	0.25 (0.22-0.28)	0.03 (0.01-0.05)	0.99 (0.97-1)	0.25	0.26
Cameroon	CAD4TB	26	0.93 (0.68-1)	0.26 (0.23-0.29)	0.03 (0.02-0.05)	0.99 (0.97-1)	0.25	0.27
Cameroon	CAD4TB	27	0.93 (0.68-1)	0.27 (0.24-0.31)	0.03 (0.02-0.05)	0.99 (0.97-1)	0.27	0.28
Cameroon	CAD4TB	28	0.93 (0.68-1)	0.28 (0.24-0.31)	0.03 (0.02-0.05)	0.99 (0.97-1)	0.27	0.29
Cameroon	CAD4TB	29	0.93 (0.68-1)	0.29 (0.25-0.32)	0.03 (0.02-0.05)	0.99 (0.97-1)	0.28	0.3
Cameroon	CAD4TB	30	0.93 (0.68-1)	0.3 (0.26-0.33)	0.03 (0.02-0.05)	0.99 (0.97-1)	0.29	0.31
Cameroon	CAD4TB	31	0.93 (0.68-1)	0.3 (0.27-0.34)	0.03 (0.02-0.05)	1 (0.97-1)	0.3	0.32
Cameroon	CAD4TB	32	0.93 (0.68-1)	0.32 (0.28-0.35)	0.03 (0.02-0.05)	1 (0.97-1)	0.31	0.33
Cameroon	CAD4TB	33	0.93 (0.68-1)	0.32 (0.29-0.36)	0.03 (0.02-0.05)	1 (0.97-1)	0.32	0.34
Cameroon	CAD4TB	34	0.93 (0.68-1)	0.33 (0.3-0.37)	0.03 (0.02-0.05)	1 (0.98-1)	0.33	0.35
Cameroon	CAD4TB	35	0.93 (0.68-1)	0.35 (0.31-0.39)	0.03 (0.02-0.05)	1 (0.98-1)	0.34	0.36
Cameroon	CAD4TB	36	0.93 (0.68-1)	0.35 (0.32-0.39)	0.03 (0.02-0.05)	1 (0.98-1)	0.35	0.36
Cameroon	CAD4TB	37	0.93 (0.68-1)	0.36 (0.32-0.4)	0.03 (0.02-0.05)	1 (0.98-1)	0.35	0.37
Cameroon	CAD4TB	38	0.93 (0.68-1)	0.36 (0.32-0.4)	0.03 (0.02-0.05)	1 (0.98-1)	0.35	0.37
Cameroon	CAD4TB	39	0.93 (0.68-1)	0.37 (0.33-0.41)	0.03 (0.02-0.05)	1 (0.98-1)	0.36	0.38
Cameroon	CAD4TB	40	0.93 (0.68-1)	0.38 (0.34-0.42)	0.03 (0.02-0.05)	1 (0.98-1)	0.37	0.39

Site	Deep Learning System	Abnormality Score	Sensitivity	Specificity	Positive Predictive Value	Negative Predictive Value	Xpert Saving	Accuracy
Cameroon	CAD4TB	41	0.93 (0.68-1)	0.39 (0.35-0.43)	0.03 (0.02-0.06)	1 (0.98-1)	0.38	0.4
Cameroon	CAD4TB	42	0.93 (0.68-1)	0.4 (0.36-0.44)	0.03 (0.02-0.06)	1 (0.98-1)	0.39	0.41
Cameroon	CAD4TB	43	0.93 (0.68-1)	0.41 (0.37-0.44)	0.03 (0.02-0.06)	1 (0.98-1)	0.4	0.42
Cameroon	CAD4TB	44	0.93 (0.68-1)	0.45 (0.42-0.49)	0.04 (0.02-0.06)	1 (0.98-1)	0.44	0.46
Cameroon	CAD4TB	45	0.93 (0.68-1)	0.53 (0.5-0.57)	0.04 (0.02-0.07)	1 (0.98-1)	0.52	0.54
Cameroon	CAD4TB	46	0.93 (0.68-1)	0.59 (0.55-0.63)	0.05 (0.03-0.08)	1 (0.99-1)	0.58	0.6
Cameroon	CAD4TB	47	0.93 (0.68-1)	0.65 (0.61-0.69)	0.06 (0.03-0.09)	1 (0.99-1)	0.64	0.66
Cameroon	CAD4TB	48	0.93 (0.68-1)	0.7 (0.66-0.73)	0.06 (0.04-0.11)	1 (0.99-1)	0.68	0.7
Cameroon	CAD4TB	49	0.87 (0.6-0.98)	0.73 (0.7-0.77)	0.07 (0.04-0.11)	1 (0.99-1)	0.72	0.74
Cameroon	CAD4TB	50	0.87 (0.6-0.98)	0.76 (0.73-0.79)	0.08 (0.04-0.13)	1 (0.99-1)	0.75	0.77
Cameroon	CAD4TB	51	0.87 (0.6-0.98)	0.79 (0.76-0.82)	0.09 (0.05-0.14)	1 (0.99-1)	0.78	0.79
Cameroon	CAD4TB	52	0.87 (0.6-0.98)	0.81 (0.78-0.84)	0.09 (0.05-0.15)	1 (0.99-1)	0.79	0.81
Cameroon	CAD4TB	53	0.87 (0.6-0.98)	0.82 (0.79-0.85)	0.1 (0.05-0.16)	1 (0.99-1)	0.81	0.83
Cameroon	CAD4TB	54	0.8 (0.52-0.96)	0.84 (0.81-0.87)	0.1 (0.05-0.17)	0.99 (0.98-1)	0.82	0.84
Cameroon	CAD4TB	55	0.8 (0.52-0.96)	0.86 (0.83-0.89)	0.12 (0.06-0.19)	0.99 (0.98-1)	0.85	0.86
Cameroon	CAD4TB	56	0.8 (0.52-0.96)	0.89 (0.86-0.91)	0.14 (0.07-0.23)	0.99 (0.99-1)	0.87	0.88
Cameroon	CAD4TB	57	0.8 (0.52-0.96)	0.9 (0.87-0.92)	0.15 (0.08-0.25)	1 (0.99-1)	0.88	0.9
Cameroon	CAD4TB	58	0.73 (0.45-0.92)	0.91 (0.88-0.93)	0.15 (0.08-0.25)	0.99 (0.98-1)	0.89	0.9
Cameroon	CAD4TB	59	0.73 (0.45-0.92)	0.92 (0.89-0.94)	0.17 (0.09-0.28)	0.99 (0.98-1)	0.9	0.91
Cameroon	CAD4TB	60	0.67 (0.38-0.88)	0.92 (0.9-0.94)	0.16 (0.08-0.27)	0.99 (0.98-1)	0.91	0.91
Cameroon	CAD4TB	61	0.67 (0.38-0.88)	0.92 (0.9-0.94)	0.17 (0.08-0.29)	0.99 (0.98-1)	0.91	0.92
Cameroon	CAD4TB	62	0.67 (0.38-0.88)	0.93 (0.91-0.95)	0.17 (0.09-0.29)	0.99 (0.98-1)	0.91	0.92
Cameroon	CAD4TB	63	0.67 (0.38-0.88)	0.93 (0.91-0.95)	0.18 (0.09-0.31)	0.99 (0.98-1)	0.92	0.93
Cameroon	CAD4TB	64	0.67 (0.38-0.88)	0.94 (0.91-0.95)	0.19 (0.09-0.32)	0.99 (0.98-1)	0.92	0.93
Cameroon	CAD4TB	65	0.67 (0.38-0.88)	0.94 (0.92-0.96)	0.2 (0.1-0.33)	0.99 (0.98-1)	0.93	0.93
Cameroon	CAD4TB	66	0.67 (0.38-0.88)	0.94 (0.92-0.96)	0.2 (0.1-0.34)	0.99 (0.98-1)	0.93	0.93
Cameroon	CAD4TB	67	0.6 (0.32-0.84)	0.94 (0.92-0.96)	0.2 (0.09-0.34)	0.99 (0.98-1)	0.93	0.94
Cameroon	CAD4TB	68	0.6 (0.32-0.84)	0.95 (0.93-0.96)	0.21 (0.1-0.36)	0.99 (0.98-1)	0.94	0.94
Cameroon	CAD4TB	69	0.6 (0.32-0.84)	0.95 (0.93-0.97)	0.22 (0.11-0.38)	0.99 (0.98-1)	0.94	0.95
Cameroon	CAD4TB	70	0.6 (0.32-0.84)	0.95 (0.94-0.97)	0.23 (0.11-0.39)	0.99 (0.98-1)	0.94	0.95
Cameroon	CAD4TB	71	0.6 (0.32-0.84)	0.96 (0.94-0.97)	0.24 (0.11-0.4)	0.99 (0.98-1)	0.94	0.95
Cameroon	CAD4TB	72	0.6 (0.32-0.84)	0.96 (0.94-0.97)	0.24 (0.11-0.4)	0.99 (0.98-1)	0.94	0.95

Site	Deep Learning System	Abnormality Score	Sensitivity	Specificity	Positive Predictive Value	Negative Predictive Value	Xpert Saving	Accuracy
Cameroon	CAD4TB	73	0.6 (0.32-0.84)	0.96 (0.95-0.98)	0.26 (0.13-0.44)	0.99 (0.98-1)	0.95	0.95
Cameroon	CAD4TB	74	0.6 (0.32-0.84)	0.97 (0.95-0.98)	0.31 (0.15-0.51)	0.99 (0.98-1)	0.96	0.96
Cameroon	CAD4TB	75	0.6 (0.32-0.84)	0.97 (0.96-0.98)	0.32 (0.16-0.52)	0.99 (0.98-1)	0.96	0.96
Cameroon	CAD4TB	76	0.53 (0.27-0.79)	0.97 (0.96-0.99)	0.32 (0.15-0.54)	0.99 (0.98-1)	0.96	0.96
Cameroon	CAD4TB	77	0.53 (0.27-0.79)	0.97 (0.96-0.99)	0.32 (0.15-0.54)	0.99 (0.98-1)	0.96	0.96
Cameroon	CAD4TB	78	0.47 (0.21-0.73)	0.97 (0.96-0.99)	0.29 (0.13-0.51)	0.99 (0.98-0.99)	0.96	0.96
Cameroon	CAD4TB	79	0.47 (0.21-0.73)	0.98 (0.96-0.99)	0.3 (0.13-0.53)	0.99 (0.98-0.99)	0.97	0.96
Cameroon	CAD4TB	80	0.47 (0.21-0.73)	0.98 (0.96-0.99)	0.3 (0.13-0.53)	0.99 (0.98-0.99)	0.97	0.96
Cameroon	CAD4TB	81	0.47 (0.21-0.73)	0.98 (0.97-0.99)	0.35 (0.15-0.59)	0.99 (0.98-0.99)	0.97	0.97
Cameroon	CAD4TB	82	0.47 (0.21-0.73)	0.98 (0.97-0.99)	0.39 (0.17-0.64)	0.99 (0.98-0.99)	0.97	0.97
Cameroon	CAD4TB	83	0.47 (0.21-0.73)	0.99 (0.97-0.99)	0.44 (0.2-0.7)	0.99 (0.98-0.99)	0.98	0.98
Cameroon	CAD4TB	84	0.47 (0.21-0.73)	0.99 (0.98-1)	0.5 (0.23-0.77)	0.99 (0.98-0.99)	0.98	0.98
Cameroon	CAD4TB	85	0.4 (0.16-0.68)	0.99 (0.98-1)	0.55 (0.23-0.83)	0.99 (0.97-0.99)	0.98	0.98
Cameroon	CAD4TB	86	0.4 (0.16-0.68)	0.99 (0.98-1)	0.6 (0.26-0.88)	0.99 (0.97-0.99)	0.99	0.98
Cameroon	CAD4TB	87	0.4 (0.16-0.68)	1 (0.99-1)	0.67 (0.3-0.93)	0.99 (0.97-0.99)	0.99	0.98
Cameroon	CAD4TB	88	0.4 (0.16-0.68)	1 (0.99-1)	0.67 (0.3-0.93)	0.99 (0.97-0.99)	0.99	0.98
Cameroon	CAD4TB	89	0.4 (0.16-0.68)	1 (0.99-1)	0.67 (0.3-0.93)	0.99 (0.97-0.99)	0.99	0.98
Cameroon	CAD4TB	90	0.4 (0.16-0.68)	1 (0.99-1)	0.86 (0.42-1)	0.99 (0.97-0.99)	0.99	0.99
Cameroon	CAD4TB	91	0.4 (0.16-0.68)	1 (0.99-1)	0.86 (0.42-1)	0.99 (0.97-0.99)	0.99	0.99
Cameroon	CAD4TB	92	0.4 (0.16-0.68)	1 (0.99-1)	0.86 (0.42-1)	0.99 (0.97-0.99)	0.99	0.99
Cameroon	CAD4TB	93	0.4 (0.16-0.68)	1 (0.99-1)	0.86 (0.42-1)	0.99 (0.97-0.99)	0.99	0.99
Cameroon	CAD4TB	94	0.33 (0.12-0.62)	1 (0.99-1)	0.83 (0.36-1)	0.99 (0.97-0.99)	0.99	0.98
Cameroon	CAD4TB	95	0.33 (0.12-0.62)	1 (0.99-1)	0.83 (0.36-1)	0.99 (0.97-0.99)	0.99	0.98
Cameroon	CAD4TB	96	0.33 (0.12-0.62)	1 (0.99-1)	0.83 (0.36-1)	0.99 (0.97-0.99)	0.99	0.98
Cameroon	CAD4TB	97	0.33 (0.12-0.62)	1 (0.99-1)	0.83 (0.36-1)	0.99 (0.97-0.99)	0.99	0.98
Cameroon	CAD4TB	98	0.33 (0.12-0.62)	1 (0.99-1)	0.83 (0.36-1)	0.99 (0.97-0.99)	0.99	0.98
Cameroon	CAD4TB	99	0.33 (0.12-0.62)	1 (0.99-1)	0.83 (0.36-1)	0.99 (0.97-0.99)	0.99	0.98
Cameroon	Lunit	0	1 (0.78-1)	0 (0-0.01)	0.02 (0.01-0.04)	NA	0	0.02
Cameroon	Lunit	0.01	1 (0.78-1)	0.28 (0.25-0.32)	0.03 (0.02-0.05)	1 (0.98-1)	0.27	0.02
Cameroon	Lunit	0.02	0.93 (0.68-1)	0.44 (0.4-0.48)	0.04 (0.02-0.06)	1 (0.98-1)	0.43	0.02
Cameroon	Lunit	0.03	0.93 (0.68-1)	0.5 (0.46-0.53)	0.04 (0.02-0.07)	1 (0.98-1)	0.49	0.04
Cameroon	Lunit	0.04	0.93 (0.68-1)	0.54 (0.5-0.58)	0.04 (0.02-0.07)	1 (0.98-1)	0.53	0.06

Site	Deep Learning System	Abnormality Score	Sensitivity	Specificity	Positive Predictive Value	Negative Predictive Value	Xpert Saving	Accuracy
Cameroon	Lunit	0.05	0.93 (0.68-1)	0.58 (0.54-0.61)	0.05 (0.03-0.08)	1 (0.99-1)	0.56	0.09
Cameroon	Lunit	0.06	0.93 (0.68-1)	0.59 (0.56-0.63)	0.05 (0.03-0.08)	1 (0.99-1)	0.58	0.13
Cameroon	Lunit	0.07	0.93 (0.68-1)	0.62 (0.58-0.65)	0.05 (0.03-0.09)	1 (0.99-1)	0.6	0.18
Cameroon	Lunit	0.08	0.93 (0.68-1)	0.65 (0.61-0.68)	0.06 (0.03-0.09)	1 (0.99-1)	0.63	0.24
Cameroon	Lunit	0.09	0.93 (0.68-1)	0.67 (0.63-0.7)	0.06 (0.03-0.1)	1 (0.99-1)	0.65	0.29
Cameroon	Lunit	0.1	0.93 (0.68-1)	0.68 (0.64-0.71)	0.06 (0.03-0.1)	1 (0.99-1)	0.67	0.33
Cameroon	Lunit	0.11	0.93 (0.68-1)	0.7 (0.66-0.73)	0.06 (0.04-0.11)	1 (0.99-1)	0.68	0.38
Cameroon	Lunit	0.12	0.93 (0.68-1)	0.71 (0.67-0.74)	0.07 (0.04-0.11)	1 (0.99-1)	0.69	0.43
Cameroon	Lunit	0.13	0.93 (0.68-1)	0.72 (0.69-0.76)	0.07 (0.04-0.12)	1 (0.99-1)	0.71	0.48
Cameroon	Lunit	0.14	0.93 (0.68-1)	0.73 (0.7-0.77)	0.07 (0.04-0.12)	1 (0.99-1)	0.72	0.54
Cameroon	Lunit	0.15	0.93 (0.68-1)	0.74 (0.71-0.78)	0.08 (0.04-0.12)	1 (0.99-1)	0.73	0.58
Cameroon	Lunit	0.16	0.93 (0.68-1)	0.76 (0.72-0.79)	0.08 (0.04-0.13)	1 (0.99-1)	0.74	0.6
Cameroon	Lunit	0.17	0.93 (0.68-1)	0.76 (0.73-0.79)	0.08 (0.05-0.13)	1 (0.99-1)	0.75	0.63
Cameroon	Lunit	0.18	0.93 (0.68-1)	0.77 (0.74-0.8)	0.08 (0.05-0.14)	1 (0.99-1)	0.75	0.67
Cameroon	Lunit	0.19	0.93 (0.68-1)	0.77 (0.74-0.8)	0.08 (0.05-0.14)	1 (0.99-1)	0.76	0.7
Cameroon	Lunit	0.2	0.93 (0.68-1)	0.78 (0.75-0.81)	0.09 (0.05-0.14)	1 (0.99-1)	0.76	0.72
Cameroon	Lunit	0.21	0.93 (0.68-1)	0.79 (0.76-0.82)	0.09 (0.05-0.15)	1 (0.99-1)	0.77	0.74
Cameroon	Lunit	0.22	0.93 (0.68-1)	0.8 (0.76-0.83)	0.09 (0.05-0.15)	1 (0.99-1)	0.78	0.75
Cameroon	Lunit	0.23	0.93 (0.68-1)	0.8 (0.77-0.83)	0.1 (0.05-0.16)	1 (0.99-1)	0.79	0.77
Cameroon	Lunit	0.24	0.93 (0.68-1)	0.8 (0.77-0.83)	0.1 (0.05-0.16)	1 (0.99-1)	0.79	0.78
Cameroon	Lunit	0.25	0.93 (0.68-1)	0.81 (0.78-0.84)	0.1 (0.06-0.16)	1 (0.99-1)	0.8	0.79
Cameroon	Lunit	0.26	0.93 (0.68-1)	0.82 (0.79-0.85)	0.1 (0.06-0.17)	1 (0.99-1)	0.8	0.8
Cameroon	Lunit	0.27	0.93 (0.68-1)	0.82 (0.79-0.85)	0.1 (0.06-0.17)	1 (0.99-1)	0.8	0.8
Cameroon	Lunit	0.28	0.93 (0.68-1)	0.82 (0.79-0.85)	0.11 (0.06-0.17)	1 (0.99-1)	0.8	0.81
Cameroon	Lunit	0.29	0.93 (0.68-1)	0.82 (0.79-0.85)	0.11 (0.06-0.17)	1 (0.99-1)	0.81	0.81
Cameroon	Lunit	0.3	0.93 (0.68-1)	0.82 (0.79-0.85)	0.11 (0.06-0.17)	1 (0.99-1)	0.81	0.82
Cameroon	Lunit	0.31	0.93 (0.68-1)	0.82 (0.79-0.85)	0.11 (0.06-0.17)	1 (0.99-1)	0.81	0.84
Cameroon	Lunit	0.32	0.93 (0.68-1)	0.83 (0.8-0.86)	0.11 (0.06-0.18)	1 (0.99-1)	0.81	0.85
Cameroon	Lunit	0.33	0.93 (0.68-1)	0.83 (0.8-0.86)	0.11 (0.06-0.18)	1 (0.99-1)	0.81	0.85
Cameroon	Lunit	0.34	0.93 (0.68-1)	0.83 (0.8-0.86)	0.11 (0.06-0.18)	1 (0.99-1)	0.82	0.86
Cameroon	Lunit	0.35	0.93 (0.68-1)	0.84 (0.81-0.87)	0.12 (0.07-0.19)	1 (0.99-1)	0.82	0.88
Cameroon	Lunit	0.36	0.93 (0.68-1)	0.85 (0.82-0.87)	0.12 (0.07-0.19)	1 (0.99-1)	0.83	0.88

Site	Deep Learning System	Abnormality Score	Sensitivity	Specificity	Positive Predictive Value	Negative Predictive Value	Xpert Saving	Accuracy
Cameroon	Lunit	0.37	0.93 (0.68-1)	0.85 (0.82-0.88)	0.12 (0.07-0.2)	1 (0.99-1)	0.83	0.89
Cameroon	Lunit	0.38	0.93 (0.68-1)	0.85 (0.82-0.88)	0.12 (0.07-0.2)	1 (0.99-1)	0.83	0.89
Cameroon	Lunit	0.39	0.93 (0.68-1)	0.85 (0.82-0.88)	0.12 (0.07-0.2)	1 (0.99-1)	0.83	0.89
Cameroon	Lunit	0.4	0.93 (0.68-1)	0.85 (0.83-0.88)	0.13 (0.07-0.2)	1 (0.99-1)	0.84	0.9
Cameroon	Lunit	0.41	0.93 (0.68-1)	0.86 (0.83-0.88)	0.13 (0.07-0.2)	1 (0.99-1)	0.84	0.9
Cameroon	Lunit	0.42	0.93 (0.68-1)	0.86 (0.83-0.88)	0.13 (0.07-0.2)	1 (0.99-1)	0.84	0.91
Cameroon	Lunit	0.43	0.93 (0.68-1)	0.86 (0.83-0.88)	0.13 (0.07-0.21)	1 (0.99-1)	0.84	0.91
Cameroon	Lunit	0.44	0.93 (0.68-1)	0.86 (0.83-0.89)	0.13 (0.07-0.21)	1 (0.99-1)	0.84	0.91
Cameroon	Lunit	0.45	0.93 (0.68-1)	0.86 (0.83-0.89)	0.13 (0.07-0.21)	1 (0.99-1)	0.85	0.92
Cameroon	Lunit	0.46	0.93 (0.68-1)	0.87 (0.84-0.89)	0.14 (0.08-0.22)	1 (0.99-1)	0.85	0.92
Cameroon	Lunit	0.47	0.93 (0.68-1)	0.87 (0.84-0.9)	0.14 (0.08-0.22)	1 (0.99-1)	0.85	0.92
Cameroon	Lunit	0.48	0.93 (0.68-1)	0.87 (0.84-0.9)	0.14 (0.08-0.23)	1 (0.99-1)	0.85	0.92
Cameroon	Lunit	0.49	0.93 (0.68-1)	0.87 (0.85-0.9)	0.14 (0.08-0.23)	1 (0.99-1)	0.86	0.92
Cameroon	Lunit	0.5	0.93 (0.68-1)	0.88 (0.85-0.9)	0.14 (0.08-0.23)	1 (0.99-1)	0.86	0.92
Cameroon	Lunit	0.51	0.93 (0.68-1)	0.88 (0.85-0.9)	0.15 (0.08-0.23)	1 (0.99-1)	0.86	0.93
Cameroon	Lunit	0.52	0.93 (0.68-1)	0.88 (0.85-0.9)	0.15 (0.08-0.23)	1 (0.99-1)	0.86	0.93
Cameroon	Lunit	0.53	0.93 (0.68-1)	0.88 (0.85-0.9)	0.15 (0.08-0.23)	1 (0.99-1)	0.86	0.93
Cameroon	Lunit	0.54	0.93 (0.68-1)	0.88 (0.86-0.91)	0.15 (0.09-0.24)	1 (0.99-1)	0.86	0.93
Cameroon	Lunit	0.55	0.93 (0.68-1)	0.88 (0.86-0.91)	0.15 (0.09-0.24)	1 (0.99-1)	0.86	0.93
Cameroon	Lunit	0.56	0.87 (0.6-0.98)	0.88 (0.86-0.91)	0.14 (0.08-0.23)	1 (0.99-1)	0.87	0.94
Cameroon	Lunit	0.57	0.87 (0.6-0.98)	0.89 (0.86-0.91)	0.15 (0.08-0.24)	1 (0.99-1)	0.87	0.94
Cameroon	Lunit	0.58	0.87 (0.6-0.98)	0.89 (0.86-0.91)	0.15 (0.08-0.24)	1 (0.99-1)	0.87	0.94
Cameroon	Lunit	0.59	0.87 (0.6-0.98)	0.89 (0.87-0.92)	0.15 (0.09-0.25)	1 (0.99-1)	0.88	0.94
Cameroon	Lunit	0.6	0.87 (0.6-0.98)	0.89 (0.87-0.92)	0.16 (0.09-0.25)	1 (0.99-1)	0.88	0.94
Cameroon	Lunit	0.61	0.87 (0.6-0.98)	0.89 (0.87-0.92)	0.16 (0.09-0.25)	1 (0.99-1)	0.88	0.95
Cameroon	Lunit	0.62	0.87 (0.6-0.98)	0.9 (0.87-0.92)	0.16 (0.09-0.26)	1 (0.99-1)	0.88	0.95
Cameroon	Lunit	0.63	0.87 (0.6-0.98)	0.9 (0.87-0.92)	0.16 (0.09-0.26)	1 (0.99-1)	0.88	0.95
Cameroon	Lunit	0.64	0.87 (0.6-0.98)	0.9 (0.87-0.92)	0.16 (0.09-0.26)	1 (0.99-1)	0.88	0.95
Cameroon	Lunit	0.65	0.87 (0.6-0.98)	0.9 (0.87-0.92)	0.16 (0.09-0.26)	1 (0.99-1)	0.88	0.95
Cameroon	Lunit	0.66	0.87 (0.6-0.98)	0.9 (0.87-0.92)	0.16 (0.09-0.26)	1 (0.99-1)	0.88	0.95
Cameroon	Lunit	0.67	0.87 (0.6-0.98)	0.9 (0.88-0.92)	0.16 (0.09-0.26)	1 (0.99-1)	0.88	0.96
Cameroon	Lunit	0.68	0.87 (0.6-0.98)	0.9 (0.88-0.92)	0.16 (0.09-0.26)	1 (0.99-1)	0.88	0.96

Site	Deep Learning System	Abnormality Score	Sensitivity	Specificity	Positive Predictive Value	Negative Predictive Value	Xpert Saving	Accuracy
Cameroon	Lunit	0.69	0.8 (0.52-0.96)	0.9 (0.88-0.92)	0.15 (0.08-0.25)	1 (0.99-1)	0.89	0.96
Cameroon	Lunit	0.7	0.8 (0.52-0.96)	0.9 (0.88-0.92)	0.15 (0.08-0.25)	1 (0.99-1)	0.89	0.96
Cameroon	Lunit	0.71	0.8 (0.52-0.96)	0.9 (0.88-0.92)	0.15 (0.08-0.25)	1 (0.99-1)	0.89	0.97
Cameroon	Lunit	0.72	0.8 (0.52-0.96)	0.9 (0.88-0.92)	0.16 (0.08-0.26)	1 (0.99-1)	0.89	0.97
Cameroon	Lunit	0.73	0.8 (0.52-0.96)	0.91 (0.88-0.93)	0.16 (0.09-0.26)	1 (0.99-1)	0.89	0.97
Cameroon	Lunit	0.74	0.8 (0.52-0.96)	0.91 (0.88-0.93)	0.16 (0.09-0.27)	1 (0.99-1)	0.89	0.97
Cameroon	Lunit	0.75	0.8 (0.52-0.96)	0.91 (0.88-0.93)	0.16 (0.09-0.27)	1 (0.99-1)	0.89	0.97
Cameroon	Lunit	0.76	0.8 (0.52-0.96)	0.91 (0.88-0.93)	0.16 (0.09-0.27)	1 (0.99-1)	0.89	0.98
Cameroon	Lunit	0.77	0.8 (0.52-0.96)	0.91 (0.89-0.93)	0.17 (0.09-0.27)	1 (0.99-1)	0.89	0.98
Cameroon	Lunit	0.78	0.8 (0.52-0.96)	0.91 (0.89-0.93)	0.17 (0.09-0.28)	1 (0.99-1)	0.9	0.98
Cameroon	Lunit	0.79	0.8 (0.52-0.96)	0.91 (0.89-0.93)	0.17 (0.09-0.28)	1 (0.99-1)	0.9	0.98
Cameroon	Lunit	0.8	0.8 (0.52-0.96)	0.92 (0.9-0.94)	0.18 (0.1-0.3)	1 (0.99-1)	0.9	0.98
Cameroon	Lunit	0.81	0.8 (0.52-0.96)	0.92 (0.9-0.94)	0.19 (0.1-0.31)	1 (0.99-1)	0.91	0.98
Cameroon	Lunit	0.82	0.8 (0.52-0.96)	0.93 (0.91-0.95)	0.2 (0.11-0.32)	1 (0.99-1)	0.91	0.98
Cameroon	Lunit	0.83	0.8 (0.52-0.96)	0.93 (0.91-0.95)	0.21 (0.11-0.34)	1 (0.99-1)	0.92	0.98
Cameroon	Lunit	0.84	0.8 (0.52-0.96)	0.93 (0.91-0.95)	0.21 (0.12-0.34)	1 (0.99-1)	0.92	0.98
Cameroon	Lunit	0.85	0.8 (0.52-0.96)	0.93 (0.91-0.95)	0.21 (0.12-0.34)	1 (0.99-1)	0.92	0.98
Cameroon	Lunit	0.86	0.8 (0.52-0.96)	0.94 (0.92-0.96)	0.23 (0.12-0.36)	1 (0.99-1)	0.92	0.98
Cameroon	Lunit	0.87	0.8 (0.52-0.96)	0.94 (0.92-0.96)	0.23 (0.12-0.36)	1 (0.99-1)	0.92	0.98
Cameroon	Lunit	0.88	0.73 (0.45-0.92)	0.94 (0.92-0.96)	0.22 (0.12-0.36)	0.99 (0.98-1)	0.93	0.98
Cameroon	Lunit	0.89	0.73 (0.45-0.92)	0.94 (0.92-0.96)	0.22 (0.12-0.37)	0.99 (0.98-1)	0.93	0.98
Cameroon	Lunit	0.9	0.73 (0.45-0.92)	0.94 (0.92-0.96)	0.23 (0.12-0.37)	0.99 (0.98-1)	0.93	0.98
Cameroon	Lunit	0.91	0.73 (0.45-0.92)	0.95 (0.93-0.97)	0.25 (0.13-0.4)	0.99 (0.98-1)	0.94	0.98
Cameroon	Lunit	0.92	0.73 (0.45-0.92)	0.96 (0.94-0.97)	0.28 (0.15-0.45)	0.99 (0.98-1)	0.94	0.98
Cameroon	Lunit	0.93	0.73 (0.45-0.92)	0.96 (0.95-0.98)	0.31 (0.16-0.48)	0.99 (0.98-1)	0.95	0.98
Cameroon	Lunit	0.94	0.67 (0.38-0.88)	0.97 (0.95-0.98)	0.33 (0.17-0.53)	0.99 (0.98-1)	0.96	0.98
Cameroon	Lunit	0.95	0.67 (0.38-0.88)	0.97 (0.96-0.99)	0.37 (0.19-0.58)	0.99 (0.98-1)	0.96	0.98
Cameroon	Lunit	0.96	0.6 (0.32-0.84)	0.98 (0.97-0.99)	0.41 (0.21-0.64)	0.99 (0.98-1)	0.97	0.98
Cameroon	Lunit	0.97	0.4 (0.16-0.68)	0.99 (0.98-0.99)	0.43 (0.18-0.71)	0.99 (0.97-0.99)	0.98	0.98
Cameroon	Lunit	0.98	0.27 (0.08-0.55)	0.99 (0.98-1)	0.5 (0.16-0.84)	0.98 (0.97-0.99)	0.99	0.98
Cameroon	Lunit	0.99	0.13 (0.02-0.4)	1 (0.99-1)	1 (0.16-1)	0.98 (0.97-0.99)	1	0.98
Cameroon	Lunit	1	0 (0-0.22)	1 (0.99-1)	NA	0.98 (0.96-0.99)	1	0.02

Site	Deep Learning System	Abnormality Score	Sensitivity	Specificity	Positive Predictive Value	Negative Predictive Value	Xpert Saving	Accuracy
Cameroon	qXR	0.01	1 (0.78-1)	0 (0-0.01)	0.02 (0.01-0.04)	NA	0	0.3
Cameroon	qXR	0.02	1 (0.78-1)	0 (0-0.01)	0.02 (0.01-0.04)	NA	0	0.45
Cameroon	qXR	0.03	1 (0.78-1)	0 (0-0.01)	0.02 (0.01-0.04)	1 (0.16-1)	0	0.51
Cameroon	qXR	0.04	1 (0.78-1)	0.01 (0.01-0.03)	0.02 (0.01-0.04)	1 (0.66-1)	0.01	0.55
Cameroon	qXR	0.05	1 (0.78-1)	0.03 (0.02-0.05)	0.02 (0.01-0.04)	1 (0.85-1)	0.03	0.58
Cameroon	qXR	0.06	1 (0.78-1)	0.07 (0.05-0.09)	0.02 (0.01-0.04)	1 (0.92-1)	0.07	0.6
Cameroon	qXR	0.07	1 (0.78-1)	0.11 (0.09-0.13)	0.02 (0.01-0.04)	1 (0.95-1)	0.11	0.62
Cameroon	qXR	0.08	1 (0.78-1)	0.17 (0.14-0.2)	0.03 (0.01-0.04)	1 (0.97-1)	0.16	0.65
Cameroon	qXR	0.09	1 (0.78-1)	0.23 (0.19-0.26)	0.03 (0.02-0.05)	1 (0.98-1)	0.22	0.67
Cameroon	qXR	0.1	1 (0.78-1)	0.28 (0.24-0.31)	0.03 (0.02-0.05)	1 (0.98-1)	0.27	0.68
Cameroon	qXR	0.11	1 (0.78-1)	0.32 (0.28-0.35)	0.03 (0.02-0.05)	1 (0.98-1)	0.31	0.7
Cameroon	qXR	0.12	1 (0.78-1)	0.37 (0.33-0.41)	0.03 (0.02-0.06)	1 (0.99-1)	0.36	0.71
Cameroon	qXR	0.13	0.93 (0.68-1)	0.42 (0.38-0.46)	0.03 (0.02-0.06)	1 (0.98-1)	0.41	0.73
Cameroon	qXR	0.14	0.93 (0.68-1)	0.47 (0.44-0.51)	0.04 (0.02-0.06)	1 (0.98-1)	0.47	0.74
Cameroon	qXR	0.15	0.93 (0.68-1)	0.53 (0.49-0.57)	0.04 (0.02-0.07)	1 (0.98-1)	0.52	0.75
Cameroon	qXR	0.16	0.93 (0.68-1)	0.57 (0.53-0.61)	0.05 (0.03-0.08)	1 (0.99-1)	0.56	0.76
Cameroon	qXR	0.17	0.93 (0.68-1)	0.59 (0.55-0.63)	0.05 (0.03-0.08)	1 (0.99-1)	0.58	0.77
Cameroon	qXR	0.18	0.93 (0.68-1)	0.62 (0.59-0.66)	0.05 (0.03-0.09)	1 (0.99-1)	0.61	0.77
Cameroon	qXR	0.19	0.93 (0.68-1)	0.66 (0.62-0.7)	0.06 (0.03-0.1)	1 (0.99-1)	0.65	0.78
Cameroon	qXR	0.2	0.93 (0.68-1)	0.69 (0.66-0.73)	0.06 (0.04-0.11)	1 (0.99-1)	0.68	0.78
Cameroon	qXR	0.21	0.93 (0.68-1)	0.71 (0.68-0.75)	0.07 (0.04-0.11)	1 (0.99-1)	0.7	0.79
Cameroon	qXR	0.22	0.93 (0.68-1)	0.73 (0.7-0.76)	0.07 (0.04-0.12)	1 (0.99-1)	0.72	0.8
Cameroon	qXR	0.23	0.93 (0.68-1)	0.75 (0.71-0.78)	0.08 (0.04-0.13)	1 (0.99-1)	0.73	0.81
Cameroon	qXR	0.24	0.93 (0.68-1)	0.77 (0.74-0.8)	0.08 (0.05-0.14)	1 (0.99-1)	0.75	0.81
Cameroon	qXR	0.25	0.93 (0.68-1)	0.78 (0.74-0.81)	0.09 (0.05-0.14)	1 (0.99-1)	0.76	0.82
Cameroon	qXR	0.26	0.87 (0.6-0.98)	0.79 (0.76-0.82)	0.08 (0.05-0.14)	1 (0.99-1)	0.77	0.82
Cameroon	qXR	0.27	0.87 (0.6-0.98)	0.8 (0.76-0.83)	0.09 (0.05-0.15)	1 (0.99-1)	0.78	0.82
Cameroon	qXR	0.28	0.87 (0.6-0.98)	0.8 (0.77-0.83)	0.09 (0.05-0.15)	1 (0.99-1)	0.79	0.82
Cameroon	qXR	0.29	0.87 (0.6-0.98)	0.81 (0.78-0.84)	0.09 (0.05-0.15)	1 (0.99-1)	0.79	0.83
Cameroon	qXR	0.3	0.87 (0.6-0.98)	0.81 (0.78-0.84)	0.09 (0.05-0.16)	1 (0.99-1)	0.8	0.83
Cameroon	qXR	0.31	0.87 (0.6-0.98)	0.82 (0.79-0.85)	0.1 (0.05-0.16)	1 (0.99-1)	0.81	0.83
Cameroon	qXR	0.32	0.87 (0.6-0.98)	0.83 (0.8-0.86)	0.11 (0.06-0.17)	1 (0.99-1)	0.82	0.83

Site	Deep Learning System	Abnormality Score	Sensitivity	Specificity	Positive Predictive Value	Negative Predictive Value	Xpert Saving	Accuracy
Cameroon	qXR	0.33	0.87 (0.6-0.98)	0.85 (0.82-0.87)	0.11 (0.06-0.19)	1 (0.99-1)	0.83	0.83
Cameroon	qXR	0.34	0.87 (0.6-0.98)	0.85 (0.83-0.88)	0.12 (0.06-0.19)	1 (0.99-1)	0.84	0.84
Cameroon	qXR	0.35	0.87 (0.6-0.98)	0.86 (0.83-0.89)	0.12 (0.07-0.2)	1 (0.99-1)	0.85	0.84
Cameroon	qXR	0.36	0.87 (0.6-0.98)	0.88 (0.85-0.9)	0.14 (0.07-0.22)	1 (0.99-1)	0.86	0.85
Cameroon	qXR	0.37	0.87 (0.6-0.98)	0.88 (0.86-0.91)	0.14 (0.08-0.23)	1 (0.99-1)	0.87	0.85
Cameroon	qXR	0.38	0.87 (0.6-0.98)	0.89 (0.86-0.91)	0.15 (0.08-0.24)	1 (0.99-1)	0.87	0.85
Cameroon	qXR	0.39	0.87 (0.6-0.98)	0.89 (0.87-0.91)	0.15 (0.08-0.25)	1 (0.99-1)	0.88	0.85
Cameroon	qXR	0.4	0.87 (0.6-0.98)	0.89 (0.87-0.92)	0.16 (0.09-0.25)	1 (0.99-1)	0.88	0.86
Cameroon	qXR	0.41	0.87 (0.6-0.98)	0.9 (0.88-0.92)	0.17 (0.09-0.27)	1 (0.99-1)	0.89	0.86
Cameroon	qXR	0.42	0.87 (0.6-0.98)	0.91 (0.88-0.93)	0.17 (0.09-0.27)	1 (0.99-1)	0.89	0.86
Cameroon	qXR	0.43	0.87 (0.6-0.98)	0.91 (0.89-0.93)	0.18 (0.1-0.29)	1 (0.99-1)	0.89	0.86
Cameroon	qXR	0.44	0.87 (0.6-0.98)	0.91 (0.89-0.93)	0.19 (0.1-0.3)	1 (0.99-1)	0.9	0.86
Cameroon	qXR	0.45	0.87 (0.6-0.98)	0.92 (0.89-0.94)	0.19 (0.1-0.3)	1 (0.99-1)	0.9	0.86
Cameroon	qXR	0.46	0.87 (0.6-0.98)	0.92 (0.89-0.94)	0.19 (0.11-0.3)	1 (0.99-1)	0.9	0.87
Cameroon	qXR	0.47	0.87 (0.6-0.98)	0.92 (0.89-0.94)	0.19 (0.11-0.3)	1 (0.99-1)	0.9	0.87
Cameroon	qXR	0.48	0.87 (0.6-0.98)	0.92 (0.9-0.94)	0.19 (0.11-0.31)	1 (0.99-1)	0.9	0.87
Cameroon	qXR	0.49	0.87 (0.6-0.98)	0.92 (0.9-0.94)	0.2 (0.11-0.32)	1 (0.99-1)	0.9	0.88
Cameroon	qXR	0.5	0.8 (0.52-0.96)	0.93 (0.9-0.95)	0.2 (0.11-0.32)	1 (0.99-1)	0.91	0.88
Cameroon	qXR	0.51	0.8 (0.52-0.96)	0.93 (0.9-0.95)	0.2 (0.11-0.32)	1 (0.99-1)	0.91	0.88
Cameroon	qXR	0.52	0.8 (0.52-0.96)	0.93 (0.91-0.95)	0.2 (0.11-0.33)	1 (0.99-1)	0.91	0.88
Cameroon	qXR	0.53	0.8 (0.52-0.96)	0.93 (0.91-0.95)	0.21 (0.11-0.33)	1 (0.99-1)	0.91	0.88
Cameroon	qXR	0.54	0.8 (0.52-0.96)	0.93 (0.91-0.95)	0.21 (0.12-0.34)	1 (0.99-1)	0.92	0.88
Cameroon	qXR	0.55	0.8 (0.52-0.96)	0.94 (0.92-0.95)	0.22 (0.12-0.36)	1 (0.99-1)	0.92	0.88
Cameroon	qXR	0.56	0.8 (0.52-0.96)	0.94 (0.92-0.95)	0.22 (0.12-0.36)	1 (0.99-1)	0.92	0.88
Cameroon	qXR	0.57	0.8 (0.52-0.96)	0.94 (0.92-0.96)	0.23 (0.12-0.36)	1 (0.99-1)	0.92	0.89
Cameroon	qXR	0.58	0.8 (0.52-0.96)	0.94 (0.92-0.96)	0.24 (0.13-0.38)	1 (0.99-1)	0.93	0.89
Cameroon	qXR	0.59	0.8 (0.52-0.96)	0.94 (0.92-0.96)	0.24 (0.13-0.39)	1 (0.99-1)	0.93	0.89
Cameroon	qXR	0.6	0.8 (0.52-0.96)	0.95 (0.93-0.96)	0.26 (0.14-0.4)	1 (0.99-1)	0.93	0.89
Cameroon	qXR	0.61	0.73 (0.45-0.92)	0.95 (0.93-0.96)	0.24 (0.13-0.4)	0.99 (0.98-1)	0.93	0.89
Cameroon	qXR	0.62	0.73 (0.45-0.92)	0.95 (0.93-0.97)	0.26 (0.14-0.41)	0.99 (0.98-1)	0.94	0.9
Cameroon	qXR	0.63	0.73 (0.45-0.92)	0.96 (0.94-0.97)	0.28 (0.15-0.44)	0.99 (0.98-1)	0.94	0.9
Cameroon	qXR	0.64	0.73 (0.45-0.92)	0.96 (0.94-0.97)	0.28 (0.15-0.44)	0.99 (0.98-1)	0.94	0.9

Site	Deep Learning System	Abnormality Score	Sensitivity	Specificity	Positive Predictive Value	Negative Predictive Value	Xpert Saving	Accuracy
Cameroon	qXR	0.65	0.73 (0.45-0.92)	0.96 (0.94-0.97)	0.29 (0.15-0.46)	0.99 (0.98-1)	0.94	0.9
Cameroon	qXR	0.66	0.73 (0.45-0.92)	0.96 (0.94-0.97)	0.29 (0.15-0.46)	0.99 (0.98-1)	0.94	0.9
Cameroon	qXR	0.67	0.67 (0.38-0.88)	0.96 (0.94-0.97)	0.28 (0.14-0.45)	0.99 (0.98-1)	0.95	0.9
Cameroon	qXR	0.68	0.67 (0.38-0.88)	0.96 (0.95-0.98)	0.29 (0.15-0.47)	0.99 (0.98-1)	0.95	0.9
Cameroon	qXR	0.69	0.67 (0.38-0.88)	0.96 (0.95-0.98)	0.29 (0.15-0.47)	0.99 (0.98-1)	0.95	0.9
Cameroon	qXR	0.7	0.67 (0.38-0.88)	0.97 (0.95-0.98)	0.32 (0.17-0.51)	0.99 (0.98-1)	0.95	0.9
Cameroon	qXR	0.71	0.67 (0.38-0.88)	0.97 (0.95-0.98)	0.33 (0.17-0.53)	0.99 (0.98-1)	0.96	0.9
Cameroon	qXR	0.72	0.67 (0.38-0.88)	0.98 (0.96-0.99)	0.38 (0.2-0.59)	0.99 (0.98-1)	0.96	0.9
Cameroon	qXR	0.73	0.53 (0.27-0.79)	0.98 (0.96-0.99)	0.36 (0.17-0.59)	0.99 (0.98-1)	0.97	0.9
Cameroon	qXR	0.74	0.53 (0.27-0.79)	0.98 (0.96-0.99)	0.36 (0.17-0.59)	0.99 (0.98-1)	0.97	0.9
Cameroon	qXR	0.75	0.53 (0.27-0.79)	0.98 (0.97-0.99)	0.4 (0.19-0.64)	0.99 (0.98-1)	0.97	0.91
Cameroon	qXR	0.76	0.53 (0.27-0.79)	0.98 (0.97-0.99)	0.4 (0.19-0.64)	0.99 (0.98-1)	0.97	0.91
Cameroon	qXR	0.77	0.53 (0.27-0.79)	0.99 (0.97-0.99)	0.47 (0.23-0.72)	0.99 (0.98-1)	0.98	0.91
Cameroon	qXR	0.78	0.53 (0.27-0.79)	0.99 (0.97-0.99)	0.47 (0.23-0.72)	0.99 (0.98-1)	0.98	0.91
Cameroon	qXR	0.79	0.47 (0.21-0.73)	0.99 (0.97-0.99)	0.44 (0.2-0.7)	0.99 (0.98-0.99)	0.98	0.91
Cameroon	qXR	0.8	0.47 (0.21-0.73)	0.99 (0.98-0.99)	0.47 (0.21-0.73)	0.99 (0.98-0.99)	0.98	0.92
Cameroon	qXR	0.81	0.47 (0.21-0.73)	0.99 (0.98-1)	0.5 (0.23-0.77)	0.99 (0.98-0.99)	0.98	0.92
Cameroon	qXR	0.82	0.47 (0.21-0.73)	0.99 (0.98-1)	0.64 (0.31-0.89)	0.99 (0.98-0.99)	0.98	0.93
Cameroon	qXR	0.83	0.4 (0.16-0.68)	0.99 (0.98-1)	0.6 (0.26-0.88)	0.99 (0.97-0.99)	0.99	0.93
Cameroon	qXR	0.84	0.4 (0.16-0.68)	1 (0.99-1)	0.75 (0.35-0.97)	0.99 (0.97-0.99)	0.99	0.93
Cameroon	qXR	0.85	0.33 (0.12-0.62)	1 (0.99-1)	0.83 (0.36-1)	0.99 (0.97-0.99)	0.99	0.93
Cameroon	qXR	0.86	0.33 (0.12-0.62)	1 (0.99-1)	0.83 (0.36-1)	0.99 (0.97-0.99)	0.99	0.94
Cameroon	qXR	0.87	0.2 (0.04-0.48)	1 (0.99-1)	0.75 (0.19-0.99)	0.98 (0.97-0.99)	0.99	0.94
Cameroon	qXR	0.88	0.2 (0.04-0.48)	1 (0.99-1)	0.75 (0.19-0.99)	0.98 (0.97-0.99)	0.99	0.94
Cameroon	qXR	0.89	0.13 (0.02-0.4)	1 (0.99-1)	1 (0.16-1)	0.98 (0.97-0.99)	1	0.94
Cameroon	qXR	0.9	0.07 (0-0.32)	1 (0.99-1)	1 (0.03-1)	0.98 (0.97-0.99)	1	0.94
Cameroon	qXR	0.91	0.07 (0-0.32)	1 (0.99-1)	1 (0.03-1)	0.98 (0.97-0.99)	1	0.95
Cameroon	qXR	0.92	0 (0-0.22)	1 (0.99-1)	NA	0.98 (0.96-0.99)	1	0.95
Cameroon	qXR	0.93	0 (0-0.22)	1 (0.99-1)	NA	0.98 (0.96-0.99)	1	0.96
Cameroon	qXR	0.94	0 (0-0.22)	1 (0.99-1)	NA	0.98 (0.96-0.99)	1	0.96
Cameroon	qXR	0.95	0 (0-0.22)	1 (0.99-1)	NA	0.98 (0.96-0.99)	1	0.97
Cameroon	qXR	0.96	0 (0-0.22)	1 (0.99-1)	NA	0.98 (0.96-0.99)	1	0.97

Site	Deep Learning System	Abnormality Score	Sensitivity	Specificity	Positive Predictive Value	Negative Predictive Value	Xpert Saving	Accuracy
Cameroon	qXR	0.97	0 (0-0.22)	1 (0.99-1)	NA	0.98 (0.96-0.99)	1	0.98
Cameroon	qXR	0.98	0 (0-0.22)	1 (0.99-1)	NA	0.98 (0.96-0.99)	1	0.98
Cameroon	qXR	0.99	0 (0-0.22)	1 (0.99-1)	NA	0.98 (0.96-0.99)	1	0.98
Cameroon	qXR	1	0 (0-0.22)	1 (0.99-1)	NA	0.98 (0.96-0.99)	1	0.98
